# Widespread selection of antibiotic resistance within hospital sewers

**DOI:** 10.1038/s41467-026-78836-8

**Published:** 2026-10-29

**Authors:** Declan A. Gray, Nguyen Thi Nhung, Roelof Dirk Coertze, Mutshiene Deogratias Ekwanzala, Noel Waters, Josef D. Järhult, Michaela Wenzel, Christina Åhrén, Jerker Fick, Carl-Fredrik Flach, D. G. Joakim Larsson

**Affiliations:** 1 Centre for Antibiotic Resistance Research in Gothenburg (CARe), University of Gothenburg, Gothenburg, Sweden; 2 Department of Infectious Diseases, Institute of Biomedicine, The Sahlgrenska Academy, University of Gothenburg, Gothenburg, Sweden; 3 Department of Medical Sciences, Zoonosis Science Center, Uppsala University, Uppsala, Sweden; 4 Department of Life Sciences, Division of Molecular Bioscience, Chalmers University of Technology, Gothenburg, Sweden; 5 Swedish Strategic Program Against Antimicrobial Resistance (Strama), Region Västra Götaland, Gothenburg, Sweden; 6 Department of Chemistry, Umeå University, Umeå, Sweden

**Keywords:** Bacterial infection, Bacterial evolution, Metagenomics, Water microbiology

## Abstract

Environmental microbiomes are sources of diverse antibiotic resistance determinants that can be mobilized and transferred to human pathogens under a selection pressure from antibiotics. Hospital sewers contain more antibiotics than most environments and are thus increasingly recognised as potential arenas for resistance evolution. However, it is unknown if resistance selection occurs there and what the drivers are. Here we show selection of antibiotic-resistant bacteria within Swedish hospital sewers. Evidence include; consistent selection of multiple resistances in *Escherichia coli* exposed to filtered wastewater from ten hospitals, but not to corresponding municipal wastewaters; in-situ enrichment of resistant over sensitive *E. coli* from wastewater to sewer biofilms at hospitals, but not at municipal sites; and higher resistance in bacteria collected from hospital sewers than from municipal sites as assessed by culturing and metagenomics. Evidence for drivers includes complete rescue of sensitive *E. coli* by addition of resistance genes to fluoroquinolones and β-lactams, and cellular effects typical for β-lactams. Measured concentrations by liquid-chromatography-tandem-mass-spectrometry compared to selective thresholds identify ciprofloxacin and cefotaxime as principal selective agents. Antibiotic concentrations and use data allow extrapolation of risks beyond Sweden. Given the absence of detectable selection at municipal sites, mitigations of risks in hospital sewers should be prioritized.

## Abstract (web summary)

Resistance selection in wastewater is poorly understood. In this study, the authors show that E. coli are selected for in Swedish hospital sewers, but not in municipal treatment plants and identify ciprofloxacin and cefotaxime as drivers that enable targeted measures to manage risks for resistance evolution.

## Introduction

Antibiotic resistance (AR) is a pressing global public health issue that caused at least 1.27 million deaths worldwide in 2019, with resistant *Escherichia coli* and *Klebsiella pneumoniae* being responsible for the highest number of attributed mortalities^1^. Resistant strains can be strongly promoted within microbiomes of patients and animals treated with antibiotics^2,3^. In addition, a growing body of evidence indicates the possibility for resistance selection by much lower environmental levels of antibiotics^4–8^. The concern that antibiotic pollution can lead to the mobilization, transfer and irreversible establishment of novel resistance factors from diverse environmental communities into human pathogens^9,10^ has emphasized the need for a multisectoral approach^11,12^.

While exceptionally high levels of antibiotics released from manufacturing undoubtedly select for AR^13–15^, there is much less conclusive evidence for resistance selection in environments contaminated by lower levels of excreted antibiotics^16,17^. Just over a third of Swedish in-patients are undergoing antibiotic therapy at any given time point^18^, similar to the situation in European hospitals in general^19^. The corresponding number for the general population in Sweden is about one in 300 people^18^. This explains the much higher levels typically found in hospital wastewater (HWW; up to hundreds of µg/L for some antibiotics) compared to municipal wastewater (typically up to a few µg/L)^20–22^. Several studies report a higher prevalence of resistant bacteria in HWW^23–25^. This observation alone is an indication but not strong evidence of resistance selection by HWW, as one can reasonably assume that patients, on average, carry more resistant faecal bacteria than the general population. Such differences in sources of bacteria may also explain the higher abundances or expression of antibiotic resistance genes (ARGs) found in hospital versus municipal sewers, while different taxonomic compositions of the sewer communities can further complicate interpretations of such data^26,27^. Comparisons of measured concentrations to effect concentrations derived from laboratory experiments are the classical way to assess environmental risks for any chemical. However, this approach relies not only on accurate determination of trace contaminants in often highly complex matrixes, but also on the ability to accurately reproduce all relevant biotic and abiotic field conditions, including mixture exposures, in the laboratory experiments.

Recently, we showed that sterile-filtered wastewater from one large Swedish hospital strongly selected for multi-resistant *E. coli*, as demonstrated by laboratory assays using both single strains and complex communities, while there was only a small tendency of selection by the corresponding municipal wastewater^28^. However, it remains unknown whether wastewater from other hospitals can also promote resistance, if similar selection occurs in situ under conditions present within the hospital sewers, and what selective agents are responsible. One aim of this study was therefore to assess evidence of in situ selection of resistance within the sewers. This was done by investigating the enrichment of resistant *E. coli* from wastewater to sewer biofilms. We also aimed to investigate how widespread AR selection is in hospital sewers as compared to corresponding municipal sites. A third aim was to identify drivers of AR selection in hospital wastewater, using different lines of evidence. Parallel generation of detailed usage data of antibiotics at studied hospitals, accompanied by measured wastewater concentrations, not only guided the identification of drivers but also served the purpose of allowing predictions of similar selective effects by wastewaters from other hospitals across the world.

In this work, we demonstrate widespread selection of AR in Swedish hospital sewers but not at municipal sites. The identification of ciprofloxacin and cefotaxime as drivers, together with detailed use data and measured concentrations, suggests selection in hospital sewers far beyond Sweden. Understanding drivers open possibilities to develop targeted measures both upstream and downstream with the intent to reduce risks for environmental selection, evolution and transmission of AR.

## Results

### Antibiotic resistance selection by hospital wastewaters is widespread

**Fig. 1:**
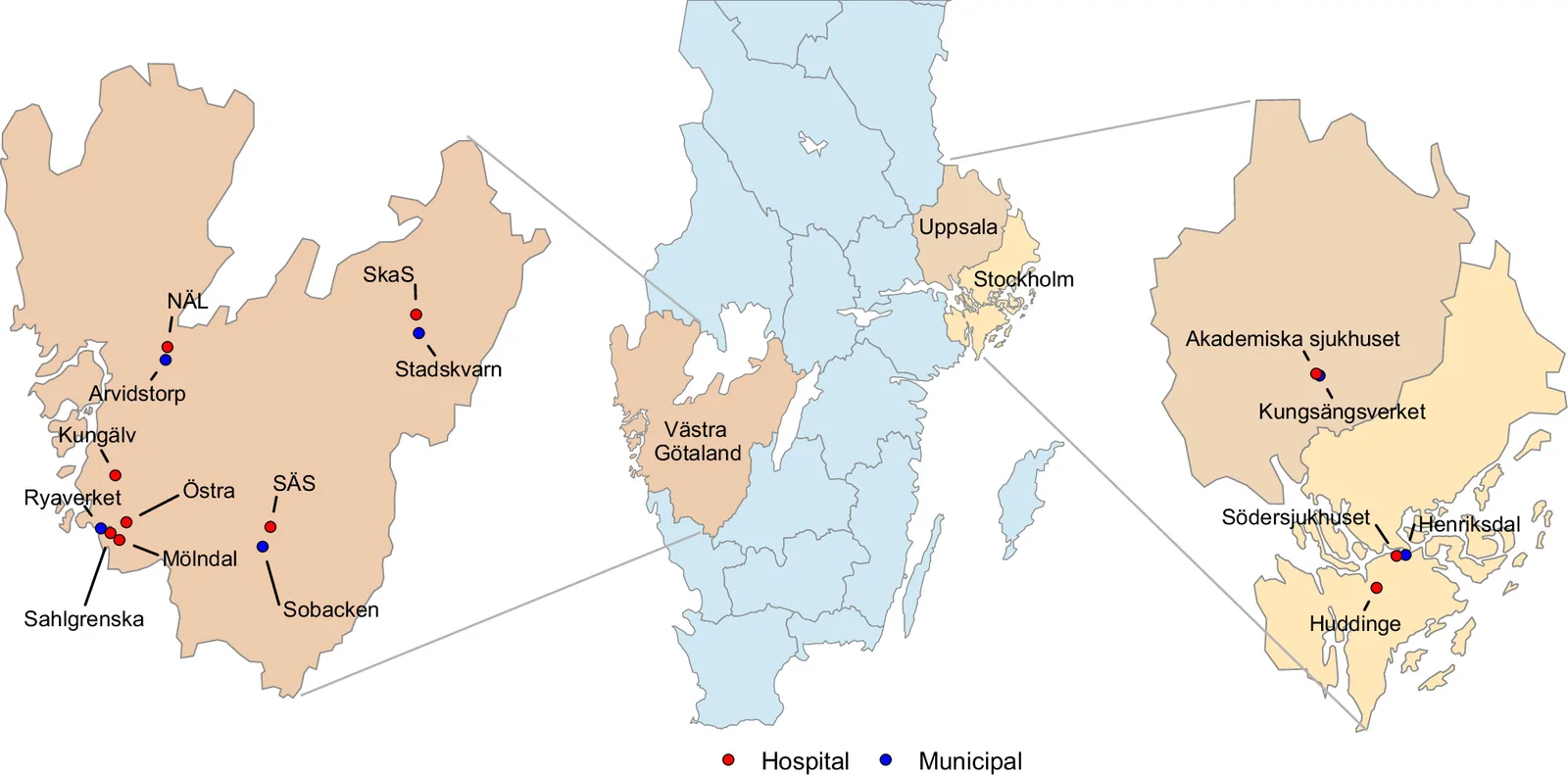
A map of wastewater sampling sites. All hospital (red) and municipal (blue) wastewater sampling sites are depicted. Source data are provided as a Source Data file. SÄS Södra Älvsborgs Sjukhus, NÄL Norra Älvsborgs Sjukhus, SkaS Skaraborgs Sjukhus.

In Sep–Nov 2024, wastewater was collected from the seven largest hospitals in the Västra Götaland Region (Sweden) and their corresponding municipal wastewater treatment facilities (Fig. 1). From each location, at least four 24 h composite samples were collected approximately 6 days apart and frozen at −20 °C. Similar samples were also taken at Uppsala’s main hospital and municipal treatment plant. Additional samples were analysed from an earlier, shorter sampling campaign at two hospitals in Stockholm and their corresponding municipal treatment site (Fig. 1). A minimum of three samples from each location were thawed, sterile-filtered to remove all bacteria naturally present in the wastewaters, and examined for their ability to select for antibiotic-resistant *E. coli* in a natural wastewater community assay^17,28^. Physiological saline was used as a negative control (both at 0 h and at the end of the experiment, 72 h^17^), while ciprofloxacin (0.1 and 0.01 mg/L) was used as positive controls.

**Fig. 2:**
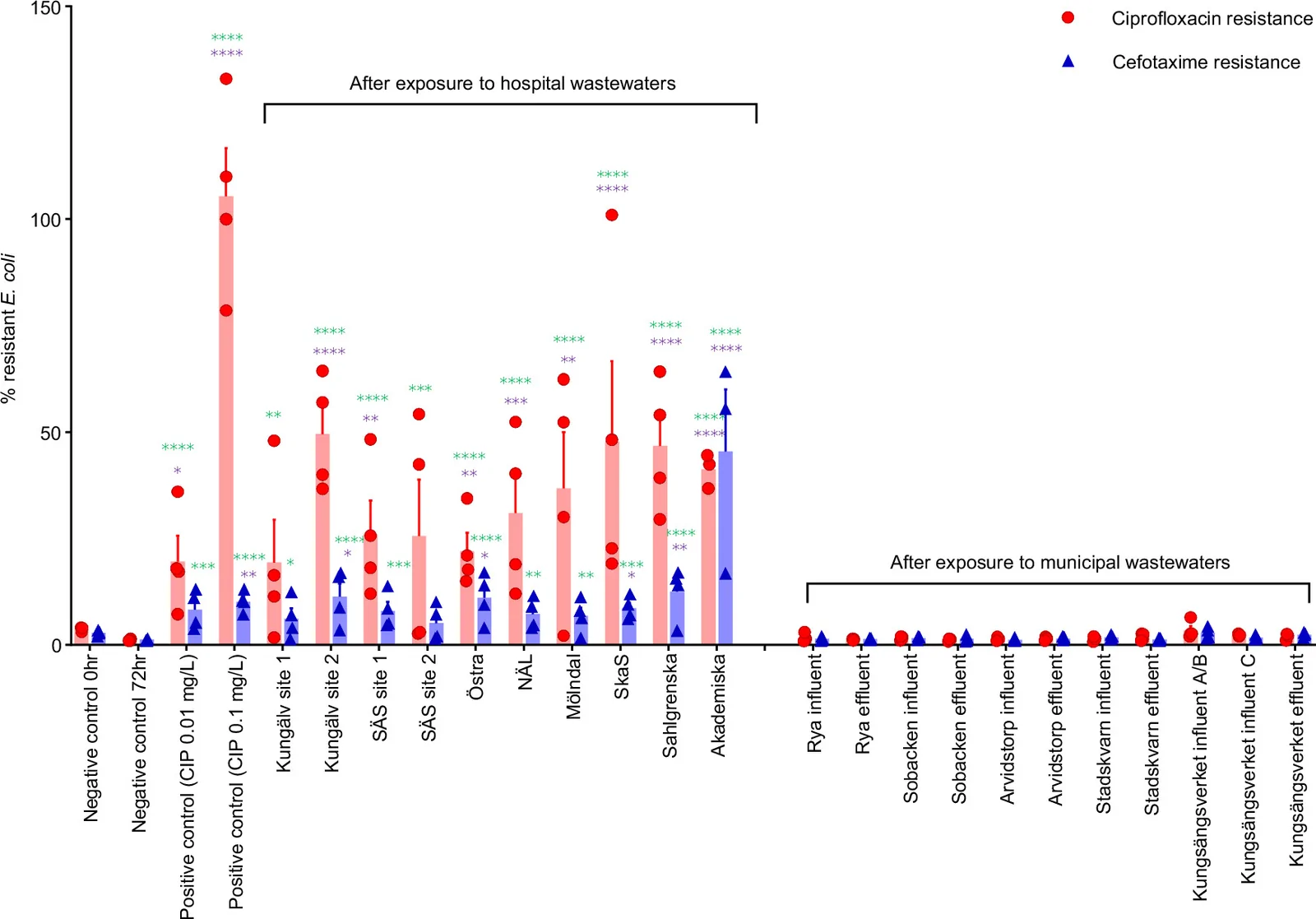
Selection of antibiotic-resistant *E. coli* in serially passaged (3 × 24 h) wastewater communities by sterile-filtered wastewaters (*n* = 4 or 5) (90%) from eight hospitals and their associated municipal wastewater treatment facilities. Growth in physiological saline with 10% LB medium served as a negative control, while 0.01 and 0.1 mg/L ciprofloxacin were positive controls. All cultures contained 10% LB medium. The percentage of resistant *E. coli* was determined by plating a fraction of the culture on CHROMagar™ ECC agar media with or without antibiotics (ciprofloxacin (0.5 mg/L, red) and cefotaxime (2 mg/L, blue)). One-way ANOVA followed by Dunnett’s multiple comparisons test was used to compare log-transformed resistance ratios at 72 h between wastewaters and physiological saline (negative control 0 h purple * and negative control 72 h green *), (^*^p < 0.05; ^**^p < 0.01, ^***^p < 0.001, ^****^p < 0.0001). Data are presented as mean values ±SEM. Source data, including all *p*-values associated with this figure are provided as a Source Data file. SÄS Södra Älvsborgs Sjukhus, NÄL Norra Älvsborgs Sjukhus, SkaS Skaraborgs Sjukhus, Rya Ryaverket.

Significant selection for ciprofloxacin and cefotaxime resistance was observed in wastewater from all studied hospitals (Fig. 2, and Supplementary Fig. 1), except Södra Älvsborgs Sjukhus (SÄS) site 2, where only ciprofloxacin resistance was selected for. There was, on average, stronger selection for resistance to ciprofloxacin than to cefotaxime, the only exception being the Uppsala hospital (Akademiska), where selection for resistance to both antibiotics was similar. Significant selection was observed regardless of the control applied (i.e. saline 0 h or 72 h), except for Kungälv site 1 and SÄS site 2, which showed significant selection only when compared with the 72 h saline control. No significant selection was detected for any of the municipal wastewaters, neither influents nor effluents (Fig. 2, and Supplementary Fig. 1).

### Evidence for on-site selection of antibiotic resistance in hospital sewer biofilms

To explore if selection could be observed within the hospital sewers, plastic plates (styrene acrylonitrile) were introduced in the sewer pipes of both hospitals and municipal sites in the Västra Götaland Region and Uppsala for approximately 30 days during the same sampling period as above. Resistance levels in naturally present *E. coli* were compared between the individual wastewater samples for each site and the corresponding biofilms by plating samples onto CHROMagar™ ECC plates with and without ciprofloxacin and cefotaxime at 0.5 mg/L and 2 mg/L, respectively.

**Fig. 3:**
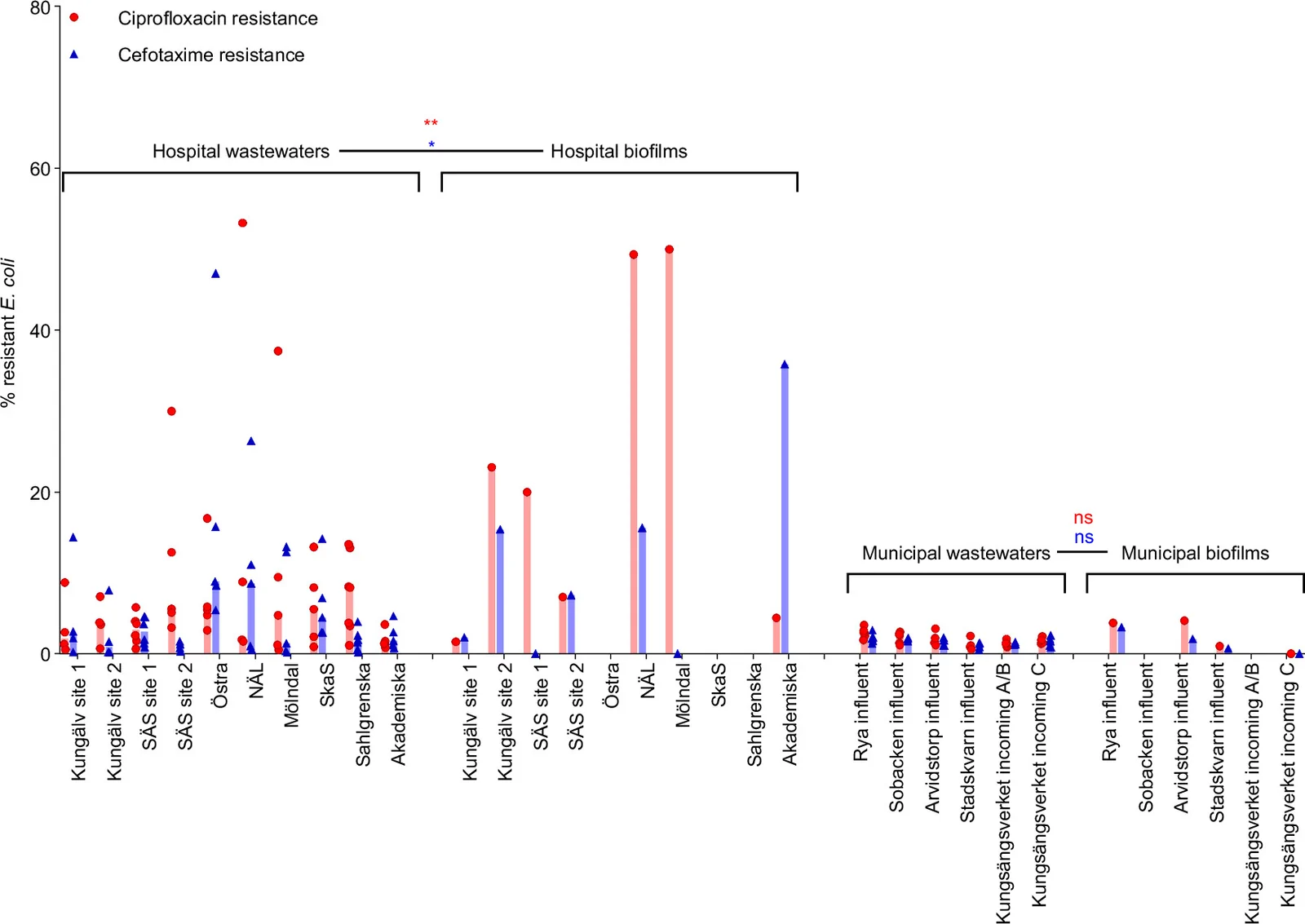
Enrichment of antibiotic-resistant *E. coli* in hospital sewer biofilms from wastewater. Wastewater (24 h composite) samples (*n* = 4 or 5) were collected at least 4 days apart from eight Swedish hospitals (10 locations) and corresponding municipal wastewater treatment plants. During this period plastic plates (styrene acrylonitrile) were submerged in the sewer systems on sites where there was a suitable access-point and allowed to develop biofilms for 25–35 days. Percentage of resistant *E. coli* was determined through plating both wastewater and disrupted biofilms on CHROMagar™ ECC agar media with or without antibiotics (ciprofloxacin (0.5 mg/L) in red and cefotaxime (2 mg/L) in blue). Each circle or triangle is a biological replicate sampled at different dates, each of which comprises the median of four technical replicates, except for hospital biofilms which have sixteen technical replicates. Red and blue bars show the median of the biological replicates. Note that total *E. coli* counts varied between samples, and hence the precision of estimated resistance ratios. A negative binomial mixed effects model approach was used to test the enrichment in resistance from wastewater to biofilm for cefotaxime and ciprofloxacin separately, via two-sided Wald *z*-tests comparing estimated resistance rates in wastewaters and biofilms on the log scale, (^**^Bonferroni adjusted *p*-value 0.001, ^*^Bonferroni adjusted *p*-value = 0.014, ns: Bonferroni adjusted *p*-value = 1). Source data are provided as a Source Data file. SÄS Södra Älvsborgs Sjukhus, NÄL Norra Älvsborgs Sjukhus, SkaS Skaraborgs Sjukhus, Rya Ryaverket municipal treatment plant in Gothenburg.

From a mixed model, analysis of the resistance rate ratios across matrix in the hospital and municipal influents showed a significantly higher level of *E. coli* resistant to ciprofloxacin (ratio = 3.11, *p* = 0.001) and to cefotaxime (ratio = 3.48, *p* = 0.014) in hospital biofilms compared to the corresponding hospital wastewaters. In contrast, at the municipal influent sites, resistance levels in *E. coli* to both antibiotics were not significantly different between biofilms and wastewaters (ciprofloxacin resistance, ratio = 1.23, *p* = 1 and cefotaxime resistance, ratio = 1.08, *p* = 1) (Fig. 3). The interaction term between matrix (biofilm and wastewater) and location type (hospital and municipal influent) was not significant, either for resistance to ciprofloxacin (*p* = 0.098, Likelihood Ratio Test) or cefotaxime (*p* = 0.142, Likelihood Ratio Test).

Additionally, resistance levels to ciprofloxacin and cefotaxime were significantly higher when comparing hospital vs municipal influent biofilms (ciprofloxacin resistance, *p* = 0.004 and cefotaxime resistance, *p* = 0.007) as well as hospital vs municipal influent wastewaters (ciprofloxacin resistance, *p* = 0.002 and cefotaxime resistance, *p* = 0.012). Given that there was no biological replication of biofilms within sites, we refrain from comparing different hospital sites to each other. The *E. coli* plate counts data are reported in the Source Data file.

**Fig. 4:**
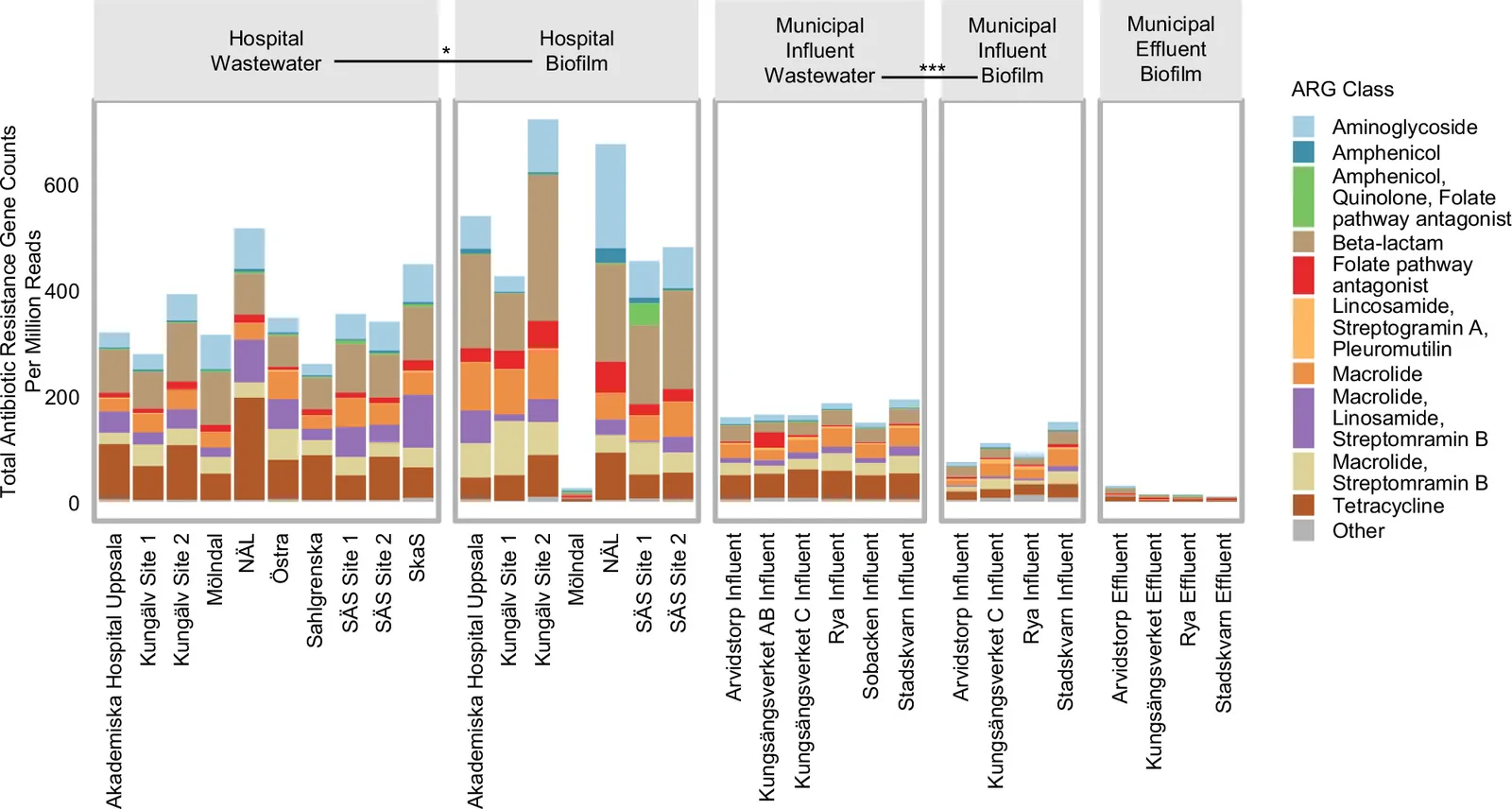
Average relative abundance of DNA Illumina reads matching antibiotic resistance genes (ARGs), normalized per million reads. Wastewater (24 h composite) samples (*n* = 4 or 5) were collected at least 4 days apart from eight Swedish hospitals (10 locations) and corresponding municipal wastewater treatment plants. During this period plastic plates (styrene acrylonitrile) were submerged in the sewer systems on sites where there was a suitable access-point and allowed to develop biofilms for 25–35 days. Shotgun metagenomic sequencing of the samples was performed using the NovaSeqXPlus system, and reads were mapped to ARG reference sequences in the ResFinder database (v.2.0.2). The bars represent the average ARG counts per million reads per ARG class across the biological replicates for each site and sample type. A negative binomial mixed effects model approach was used to test the enrichment in total ARG counts per million reads from wastewater to biofilm in hospital and municipal influent sites separately, via two-sided Wald *z*-tests comparing estimated total ARG counts per million reads on the log scale (^*^Bonferroni adjusted *p*-value = 0.03, ^***^Bonferroni adjusted *p*-value < 0.001). Source data are provided as a Source Data file. SÄS Södra Älvsborgs Sjukhus, NÄL Norra Älvsborgs Sjukhus, SkaS Skaraborgs Sjukhus, Rya Ryaverket municipal treatment plant in Gothenburg.

While the significant enrichment of resistant *E. coli* from hospital wastewater to biofilms provides direct field evidence of resistance selection (in conjunction with the experimental data), it does not reveal whether similar selection occurs in other bacterial taxa. Metagenomics analyses of antibiotic resistance genes (ARGs) can provide a broader picture, but can not separate selection of resistant strains within species from simple shifts in taxonomy that could result in changes in ARGs in either direction, unrelated to selection for acquired resistance traits^27^. In the context of establishing evidence for selection, metagenomic sequencing by Illumina technology followed by analyses of relative abundances of total mobile ARGs, as also done here (Fig. 4), should therefore at most be seen as supportive or exploratory. Analyses based on a mixed model revealed a highly significant interaction term between matrix and location type (*p* < 0.001, Likelihood Ratio Test). Comparisons across matrix within the individual location types indicated a marginally significant enrichment of total ARGs from hospital wastewater to hospital biofilms (*p* = 0.030) but a clearly significant reduction of total ARGs from wastewater to biofilm at municipal influent sites (*p* < 0.001). There was too little DNA recovered for metagenomic analyses of the municipal effluents, precluding assessment of enrichment here. However, it is noteworthy that the total ARGs (per million reads) were strongly reduced in municipal effluent biofilms compared to influent biofilms (2-sample *t*-test on the difference in log_2_(total ARGs per million reads) (*t* = −6.776, *df* = 5.02, *n* = 8, *p* = 0.001) Fig. 4). Overall PERMANOVA (*R*² = 0.37, *F* = 18.47, *p* = 0.001), as well as subsequent pairwise comparisons of the taxonomic composition (genus level) showed significant differences in beta-diversity between all matrix and location type combinations, indicating plausible effects of taxonomy on overall ARG counts (Supplementary Fig. 2, and Supplementary Data 1). ARG counts were much lower in the biofilm sample from the Mölndal hospital compared to other hospital biofilms (Fig. 4). Also, its taxonomic composition was distinct from the other biofilm samples (Supplementary Fig. 2), reflected in exceptionally few *E. coli* isolated. Hence, the low ARG count could very well have been a result of a dominance of taxa prone to carrying fewer mobile ARGs overall than did other biofilms.

### Identifying antibiotics selecting for multiple resistances in *E. coli* and *K. pneumoniae*

**Fig. 5:**
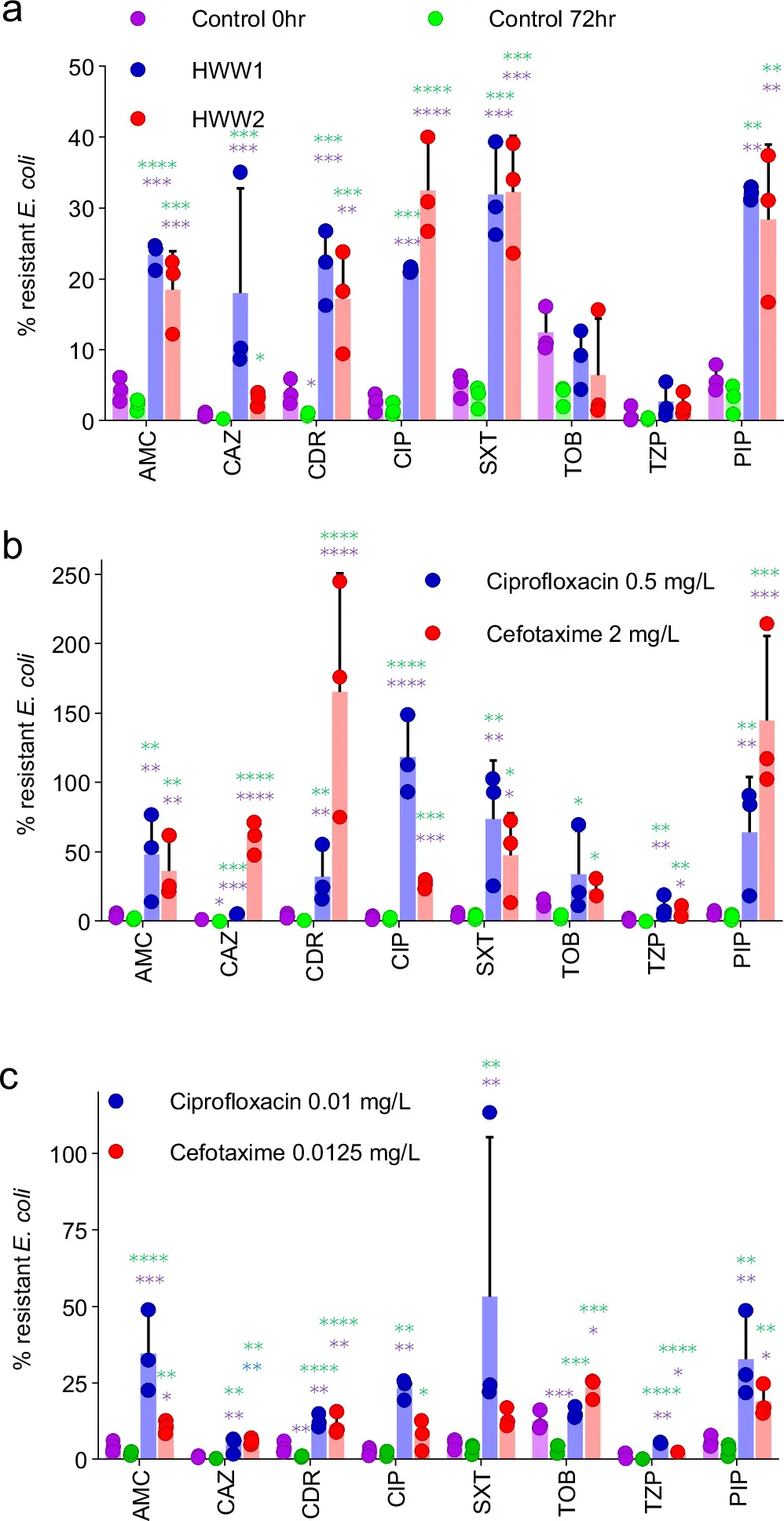
Measured concentrations of ciprofloxacin and cefotaxime select for resistance to multiple antibiotics. Selection for antibiotic-resistant* E. coli* in serially passaged wastewater communities (*n* = 3) by two sterile-filtered wastewaters (90%) from the Sahlgrenska University Hospital (HWW1 and HWW2; **a**) and by single antibiotics (ciprofloxacin and cefotaxime) at breakpoint concentrations (**b**) or at concentrations measured in the hospital wastewaters (**c**). Three complex municipal wastewater influent communities were exposed to either physiological saline (control), wastewaters or single antibiotics in saline and supplemented with 10% LB medium for 72 h (3 passages). The percentage of resistant *E. coli* was determined by plating a fraction of the culture on CHROMagar™ ECC agar with and without eight antibiotics at EUCAST breakpoint concentrations. One-way ANOVA followed by Dunnett’s multiple comparisons test was used to compare log-transformed resistance ratios of 72 h data to physiological saline at 0 or 72 h as two alternative controls (control 0 h purple * and control 72 h green *), (^*^p < 0.05; ^**^p < 0.01, ^***^p < 0.001, ^****^ *p* < 0.0001). Data are presented as mean values ±SEM. All source data, including all *p*-values associated with this figure are provided as a Source Data file. AMC amoxicillin-clavulanic acid, CAZ ceftazidime, CDR cefadroxil, CIP ciprofloxacin, SXT trimethoprim-sulfamethoxazole, TOB tobramycin, TZP piperacillin-tazobactam, PIP piperacillin.

Our work to identify selective agents in HWW initially focused on two 24 h composite samples from the Sahlgrenska University Hospital collected in January 2022 (HWW1 and HWW2), 2 years before the broad sampling described above. These samples were sterile filtered before freezing and kept at −20 °C over the course of analyses described below, the consequences of which will be discussed later. Both samples selected for *E. coli* resistant to six of the eight tested antibiotics, respectively, using the same sewage community assay (Fig. 5A), consistent with our earlier findings^28^. Antibiotics co-select for resistances across classes, exemplified here by exposing the same community to ciprofloxacin or cefotaxime only (Fig. 5B). Therefore, the underlying drivers cannot be readily inferred from the resistance patterns observed. Multi-drug resistance was also selected for in *K. pneumoniae* when exposed to both hospital wastewaters (HWW1 and HWW2) (Supplementary Information, Supplementary Figs. 3 and 4).

Antibiotic usage data from all connected wards at the Sahlgrenska University Hospital during the 3 days immediately prior to each of the 2 sampling days was used to narrow the focus on possible drivers (Supplementary Data 2). Out of 31 used antibiotics, the intravenously administered drug combination piperacillin and tazobactam (β-lactamase inhibitor) had the highest quantity used, with an average of 689 and 89 g respectively per day, followed by cefotaxime (73 g) and cloxacillin (57 g). Ciprofloxacin had an average daily, combined (oral and intravenous administration), usage of 14.4 g. Based on usage, antibiotic spectra, and feasibility for analyses, fifteen antibiotics were chosen for analyses in HWW1 and HWW2 by liquid chromatography coupled to tandem mass spectrometry (LC-MS/MS; Supplementary Data 3). Drugs are metabolised and excreted differently. Consequently, highly used antibiotics will not necessarily be found at high concentrations in the wastewater. The top two average concentrations across the two HWW samples were cefotaxime (16,115 ng/L) and ciprofloxacin (14,192 ng/L), followed by metronidazole (8718 ng/L) and cefadroxil (8265 ng/L).

To tentatively identify selective antibiotic(s), measured concentrations were compared to the corresponding lowest one percentile of minimal inhibitory concentrations (MICs) for *E. coli*^29^ (Supplementary Data 3), building on previously published principles for predicting selective concentrations^30^. Measured ciprofloxacin and cefotaxime concentrations exceeded their corresponding lowest percentile of MICs, whereas all other antibiotics were far below. Hence, we exposed three municipal wastewater communities to concentrations of ciprofloxacin (0.01 mg/L) and cefotaxime (0.0125 mg/L) approximately matching the lowest measured concentrations in the wastewaters. Ciprofloxacin (0.01 mg/L) selected significantly for *E. coli* resistance to all tested antibiotics, whereas cefotaxime (0.0125 mg/L) did so for seven out of eight antibiotics (Fig. 5C). Measured concentrations of ciprofloxacin and cefotaxime also selected for resistant *K. pneumoniae*. (Supplementary Information, Supplementary Figs. 3 and 4).

### Hospital wastewater exposure causes rapid cellular effects in *E. coli*, consistent with effects of β-lactam antibiotics

**Fig. 6:**
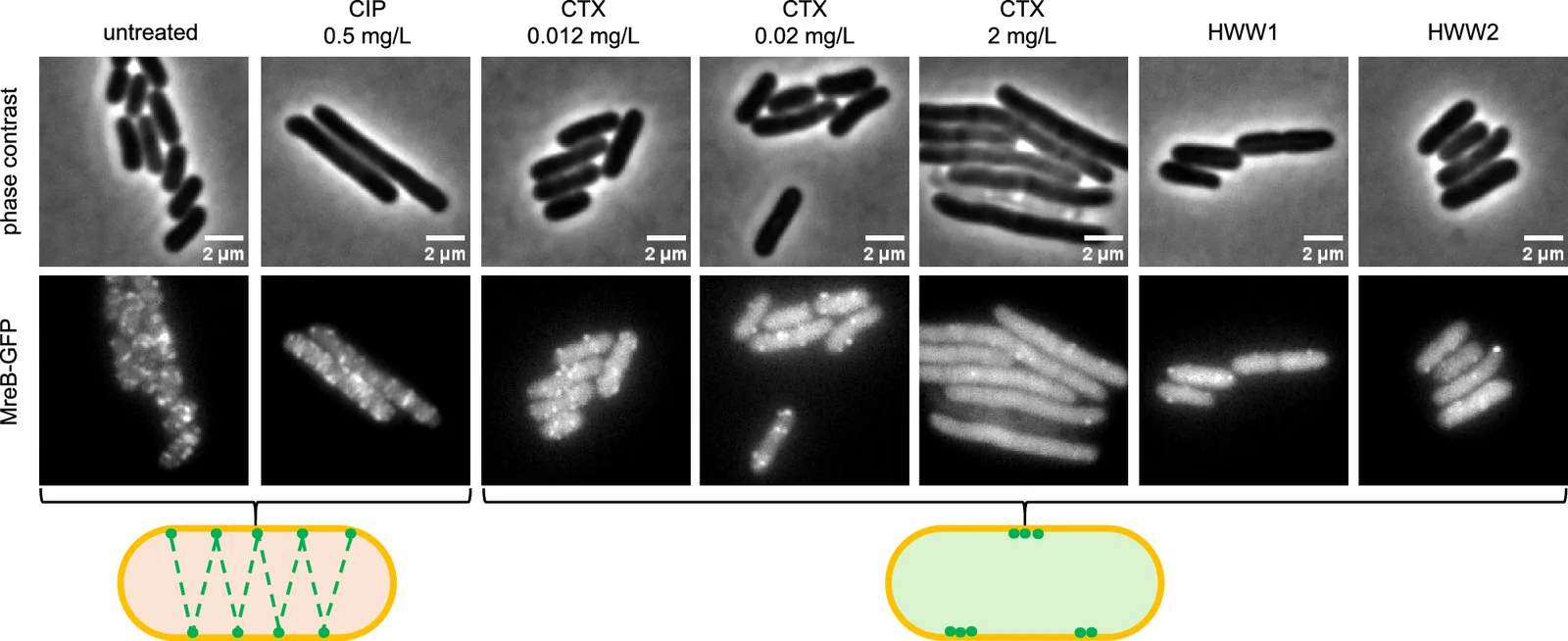
Delocalisation of MreB-msfGFP by hospital wastewater. Cultures of HS524 (MG1655 *mreB-msfgfp*) were exposed to different concentrations of cefotaxime, ciprofloxacin at the clinical breakpoint concentration, or to sterile-filtered wastewater samples from the Sahlgrenska university hospital (HWW1 and HWW2) and examined under the microscope to assess the localisation of MreB (*n* = 3). When cell wall synthesis is intact, MreB localizes in distinct, clearly membrane-associated foci along the lateral cell axis. The inhibition of cell wall synthesis by antibiotics, such as β-lactams, abolishes this pattern and causes the detachment of the protein from the membrane, becoming apparent in an increase of diffuse intracellular GFP signal and reduction of the number of membrane-associated foci. CTX cefotaxime, CIP ciprofloxacin, HWW1 hospital wastewater 1, HWW2 hospital wastewater 2, GFP green fluorescent protein.

If indeed cefotaxime, or any other β-lactam, is a strong selective agent in wastewater, we hypothesized that wastewater exposure should cause similar cellular phenotypes as treatment with β-lactams. Cell wall synthesis inhibitors, including β-lactams, inhibit peptidoglycan crosslinking and cause distinctive phenotypic effects. One such effect is the disturbance of the localization of the peripheral inner membrane protein MreB, an actin homologue that orchestrates lateral cell wall synthesis in rod-shaped bacteria, including *E. coli*^31–33^. The protein forms filaments that move along the lateral cell axis in a spiralling motion, which appears as characteristic patterns of distinct foci along the lateral cell membrane. The disruption of this pattern serves as a robust and specific reporter for cell wall synthesis inhibition^34,35^. The normal localization pattern of distinct, membrane-associated MreB foci was clearly apparent in untreated cells (Fig. 6). Treatment with cefotaxime at all tested concentrations (0.01, 0.02, 2 µg/mL) prominently abolished this pattern, resulting in detachment of the protein from the membrane, resulting in displacement of the fluorescent signal into the cytosol with only a few membrane foci remaining. In addition to this characteristic phenotype, cell elongation and lysis were observed at the highest tested concentration, a consequence of the inhibition of crosswall formation during cell division by β-lactams. Exposure to wastewater for 1 h elicited the same MreB delocalisation phenotype caused by all concentrations of cefotaxime (Fig. 6). This observation provides a further line of evidence that β-lactam antibiotics are present in the investigated HWWs at high enough concentrations to rapidly affect *E. coli* cellular functions. Exposure to ciprofloxacin did not induce a similar MreB phenotype, as expected.

MreB delocalisation is not strictly limited to cell wall synthesis inhibitors but can also be caused by compounds that dissipate the bacterial membrane potential^35,36^. To exclude the non-specific impact of other wastewater components other than β-lactams, localization of the membrane potential-dependent protein MinD was investigated. MinD is involved in cell division site regulation and in *E. coli* oscillates from cell pole to cell pole. This oscillation depends on the presence of an intact membrane potential and is abolished by depolarisation^36^. Thus, MinD has emerged as a reliable proxy for assessing membrane depolarisation effects. Clear pole-to-pole oscillation was observed in untreated control samples, while cells treated with the proton ionophore carbonyl cyanide m-chlorophenyl hydrazone (CCCP, positive control) showed a completely displaced GFP signal (Supplementary Fig. 6, and Videos S1 and 2). Samples incubated in wastewater maintained normal pole-to-pole oscillation (Supplementary Fig. 6, and Videos S3 and 4), showing that the membrane potential indeed remained intact. Thus, the observed MreB delocalisation effects are not due to nonspecific membrane effects but are attributable to the presence of cell wall synthesis inhibitors, such as β-lactams.

### Restored growth of isogenic *E. coli* constructs identify quinolones and β-lactams as the main drivers of selection

**Fig. 7:**
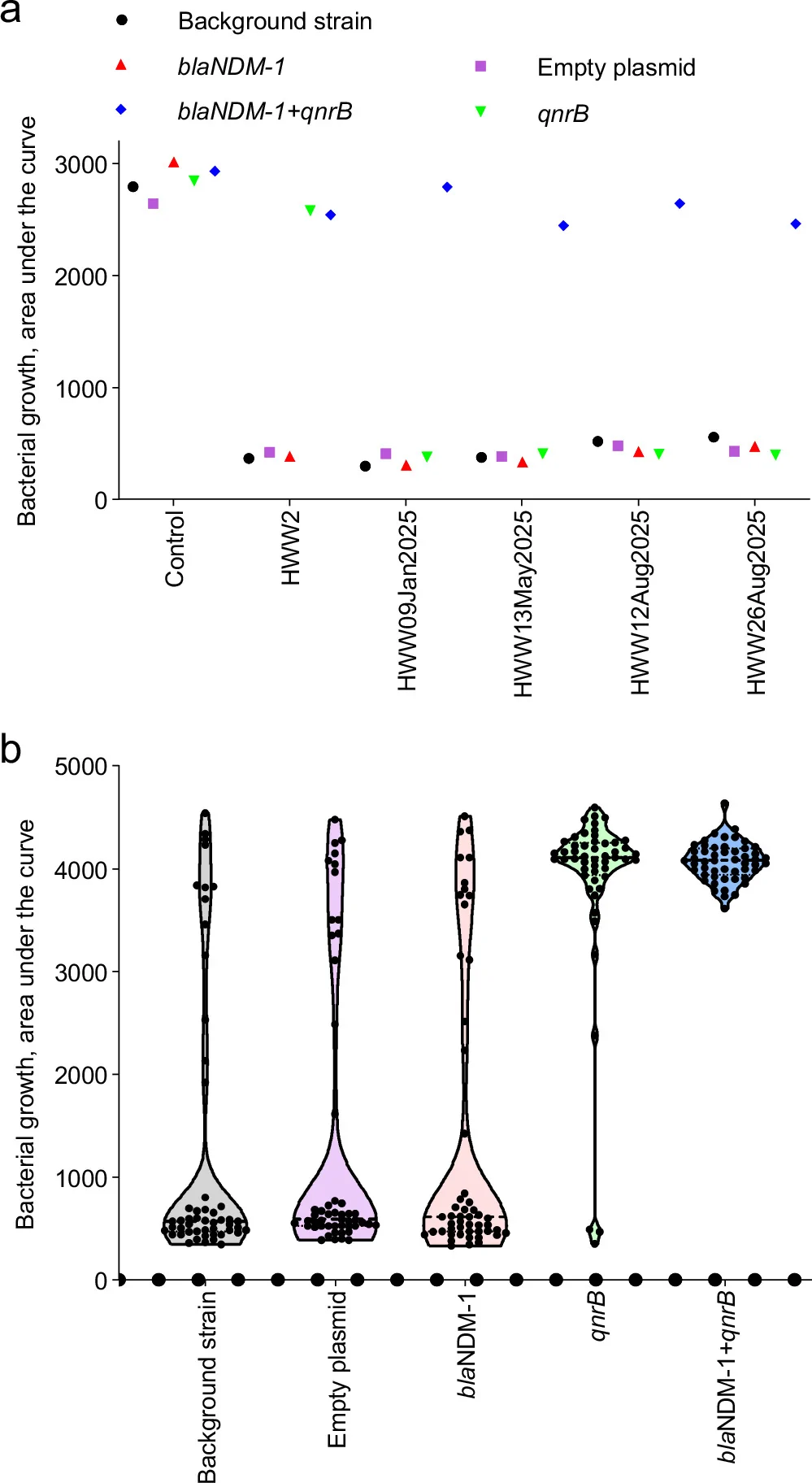
Isogenic *E. coli* constructs carrying β-lactam and/or fluoroquinolone resistance genes restore wastewater-inhibited growth. **a** The growth of five strains were assessed in physiological saline (control), and one old (HWW2, 2022) and four new (2025) hospital wastewater samples from the Sahlgrenska University Hospital. **b** The same five strains were then assessed for their ability to grow in multiple hospital wastewaters collected from the Västra Götaland Region (Sahlgrenska, Södra Älvsborgs Sjukhus, Norra Älvsborgs Sjukhus, Skaraborgs Sjukhus, Kungälv and Östra) plus Akademiska hospital located in Uppsala. Note that all samples in **a** were sterile-filtered before freezing, whereas all samples in **b** were filtered after freeze-thawing. The latter approach (Fig. 6) as well as long-term storage reduces β-lactam concentrations. All growth assays were performed in 90% solution (physiological saline or hospital wastewater) with 10% LB media using an OmniLog™ system and dye A. MC221 is the background strain isolated from Swedish wastewater. Source data are provided as a Source Data file. HWW hospital wastewater.

If quinolones and β-lactams are the dominating drivers of selection, we postulated that it should be feasible to restore the growth of a strain incapable of growing in HWW through providing resistance to quinolones and β-lactams. A set of isogenic *E. coli* strains was constructed based on a sensitive *E. coli* strain (MC221) isolated from municipal sewage. The addition of the broad-spectrum β-lactam (carbapenemase) resistance gene *bla*NDM-1 and the quinolone resistance gene *qnrB* individually or in combination increased resistance to β-lactams and quinolones, but not to any other antibiotic class (Supplementary Data 4 and 5). Note that the addition of these genes was an attempt to create a functional rescue tool and establish causality, not to claim that these specific genes are selected in the sewers. MC221 only required the presence of *qnrB* to grow in HWW2 from 2022 (Fig. 7A), suggesting ciprofloxacin is the major antibiotic causing the selective effect observed in section ‘Identifying antibiotics selecting for multiple resistances in *E. coli* and *K. pneumoniae*’. While effects attributable to cefotaxime specifically (Fig. 5C) or β-lactams (Fig. 7A) had earlier been shown for these samples, it should be noted that MC221 and the corresponding isogenic strains have a lowest MIC for cefotaxime of 0.03 μg/mL which is above the average concentration (0.0125 μg/mL) measured in HWW1 also shown to be selective in a community assay (Fig. 5C). This highlights a limitation of simple growth assays of individual isogenic strains to assess more subtle selection pressures. Still, the observation triggered our suspicion of a reduction of some antibiotics, including cefotaxime, during storage at −20 °C, something we could also find reports of in the literature^37,38^. At the time these assays were performed, only a small volume of HWW1 remained. To assess if long-term storage was likely a cause for losses, we collected additional HWWs during 2025, and both *bla*NDM-1 and *qnrB* were indeed required for the MC221 strain to grow in these (Fig. 7B). This demonstrates a combined selection pressure from quinolones and β-lactams in the newer wastewater samples from the Sahlgrenska University Hospital.

Wastewater from the hospitals in Västra Götaland and Uppsala, as well as corresponding municipal sites sampled in 2024, was also assessed using the five isogenic strains. The strain carrying *bla*NDM-1 and *qnrB* genes grew in all hospital wastewaters (Fig. 7B, and Supplementary Fig. 6), while the *qnrB* strain grew in most but not all. The *bla*NDM-1, empty plasmid and background strains did not grow in most of the hospital wastewaters collected (Fig. 7B, and Supplementary Fig. 6). All five strains grew well in all municipal wastewaters (Supplementary Fig. 7). In the older Stockholm samples from 2022, quinolone resistance was needed for *E. coli* to grow in some hospital samples, whereas β-lactam resistance was never required (Supplementary Fig. 6). Overall, these results demonstrate the widespread need for quinolone resistance for growth in Swedish hospital wastewaters, while β-lactam resistance was important for certain wastewaters. It also showed that no other resistances were critically important for the growth of *E. coli*. The growth of *K. pneumoniae* could also be restored through increasing ciprofloxacin resistance of strains unable to previously grow in HWW via serial passaging in the presence of ciprofloxacin. (Supplementary Results and Supplementary Fig. 8, and Supplementary Data 6 and 7).

**Fig. 8:**
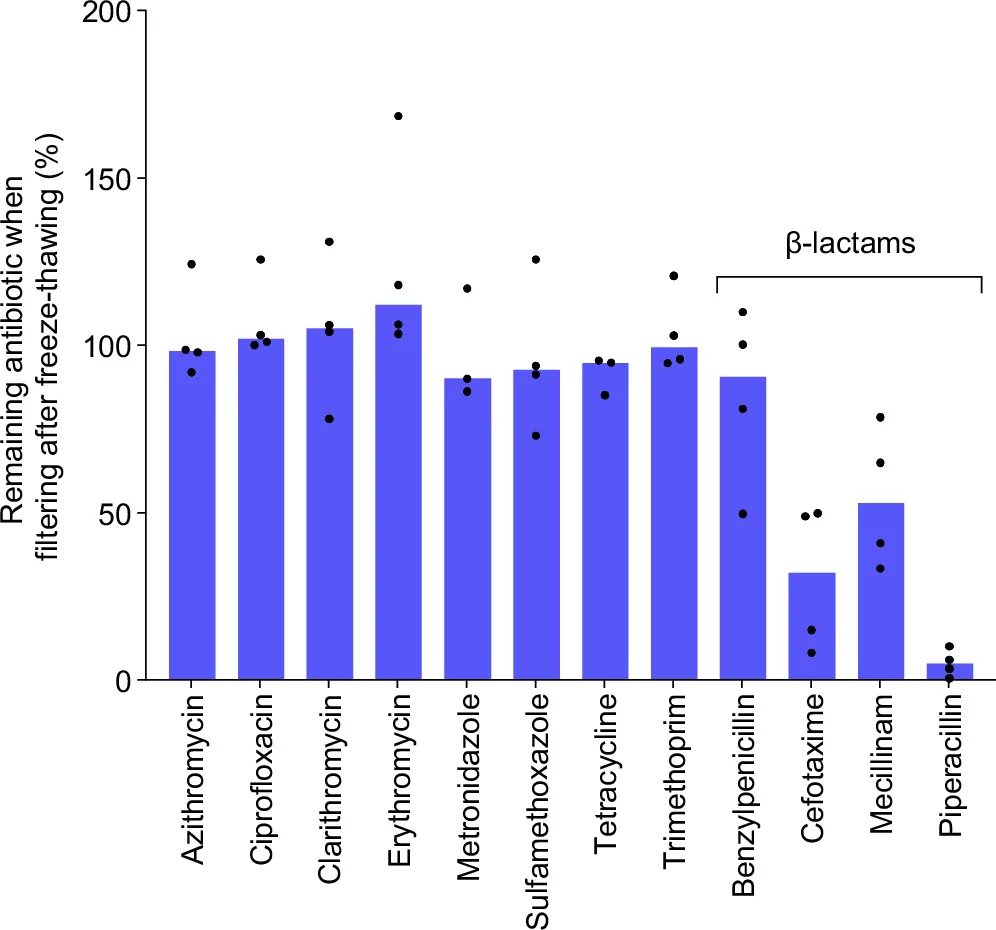
Freezing and thawing unfiltered hospital wastewater leads to reduced concentrations of several β-lactams but not of other antibiotics. Wastewaters (24 h composite) were collected on four occasions in September and October 2025 from the Sahlgrenska University Hospital (*n* = 4). For each sample, one aliquot was filtered immediately and frozen at −80 °C (control). Another aliquot was frozen whole at −20 °C for 1–2 days, then thawed, filtered immediately, frozen and stored at −80 °C until analyses of antibiotics by LC-MS/MS. Antibiotic concentrations of samples frozen whole are expressed in relation to corresponding controls. The blue bars show the median of the four samples, and the dots represent each sample. Source data are provided as a Source Data file. Actual measured concentrations can be found in Supplementary Data 8.

Given the pronounced growth-inhibitory effects of β-lactams in the 2025 Sahlgrenska hospital samples (Fig. 7A), but their absence in most samples from the regional survey and from Uppsala and Stockholm, we suspected a sampling-related effect on β-lactams (in addition to long-term storage). Samples from the larger regional sampling as well as from Stockholm and Uppsala were frozen without prior filtration (filtered after thawing). By parallel testing of filtering either before or after freezing in four new samples, we observed a marked reduction in concentration for several β-lactams (in particular cefotaxime, piperacillin and mecillinam) when freezing samples before filtering (Fig. 8, and Supplementary Data 8). This was accompanied by a partial loss of dependency of *bla*NDM-1 for growth in most samples (Supplementary Fig. 9). Notably, no other classes of antibiotics measured were affected by the different sampling procedures (Fig. 8, and Supplementary Data 8).

Analyses of antibiotics from the broad wastewater sampling in the Västra Götaland Region, Uppsala, and Stockholm confirmed that concentrations of ciprofloxacin in most hospital wastewaters were higher or around the MIC of the isogenic strains not carrying *qnrB* (Supplementary Fig. 9). Given the demonstrated reduction of β-lactams associated with freeze-thawing whole effluents as applied for these samples (Fig. 8, and Supplementary Data 8), concentration data were considered misleading for this group of antibiotics and are therefore not reported. No other antibiotics were found at concentrations close to reported MICs for *E. coli*. For all antibiotics, concentrations were consistently higher in hospital wastewaters compared to municipal wastewaters (Supplementary Data 9).

Extrapolation of risks for selection to additional hospital sewers can be facilitated both by comparisons with antibiotic concentrations and usage data. Therefore, at each of the seven Västra Götaland hospitals, detailed antibiotic usage data for the 3 days immediately prior to each sampling date were compiled on an aggregated level (Supplementary Data 10). Despite some quantitative differences, it was largely the same antibiotics that were common at all sites. The median relative usage of ciprofloxacin and cefotaxime (expressed as the proportion of the sum of all used Defined Daily Doses of all antibiotics) was 4.72 and 7.17, respectively, for the Sahlgrenska University Hospital and varied between 1.72–7.07 for ciprofloxacin and 0–8.63 for cefotaxime for all regional hospitals (Supplementary Data 11 and 12).

## Discussion

This study shows widespread selection of antibiotic-resistant *E. coli* by wastewater from ten major hospitals in eight Swedish cities. The restored growth in engineered, isogenic *E. coli* strains showed that quinolones and β-lactams are the only major growth-inhibiting antibiotic classes, and thus the dominant drivers behind the observed resistance selection. This conclusion is supported by several lines of evidence, further pinpointing ciprofloxacin and cefotaxime as selective agents. Knowing the identity of the main drivers opens possibilities for targeted mitigations, both upstream (usage) and downstream (wastewater treatment). The comprehensive usage data of antibiotics at seven hospitals not only guided chemical analyses and selection experiments but also enabled the extrapolation of findings of resistance selection to other hospitals around the world with similar or higher usage of quinolones and/or β-lactams. The absence of observable selection by all municipal wastewaters does not exclude that some level of selection may still take place, but it indicates that it should be a priority to reduce environmental resistance selection in hospital sewers.

While there are numerous reports demonstrating risks that human use of pharmaceuticals could have effects in the environment, the overwhelming majority rely exclusively on measured concentrations compared to effects observed in artificial exposure experiments^39–41^. In contrast to direct observations of effects in the field where exposure is not manipulated, such conclusions rely heavily on accurate measurements of drug levels at trace concentrations, preserved bioavailability, absence of inhibitory effects through mixture exposure, biotic interactions, and abiotic conditions that allow effects to manifest, and sometimes also extrapolations across species. Kraupner et al.^5^ showed that exposure to an average of 8.8 µg/L ciprofloxacin led to enrichment of resistant *E. coli* in sewer biofilms in controlled laboratory experiments (no enrichment observed at 1.2 µg/L, but notice the large span between tested concentrations). To our best knowledge, the selection of resistant *E. coli* from wastewater to biofilms in hospital sewers provides the first evidence of effects observed in the field that can be linked to excreted human pharmaceuticals since the case of ethinylestradiol and feminization of fish in the late 1990s^42–44^. The absence of detectable enrichment of resistant *E. coli* from municipal sewage to biofilms indicates that the enrichment from hospital sewage to biofilms is not simply a consequence of a higher ability of resistant *E. coli* to form biofilms. This is further supported by the enrichment of total ARGs from wastewater to biofilms seen at hospitals, but not at municipal sites. One cannot exclude additional drivers, but the observation that the overwhelming majority of the growth reduction observed in planktonic *E. coli* after hospital wastewater exposure is abolished by the addition of resistance genes to fluoroquinolones and β-lactams suggests other drivers are less important than these two classes of antibiotics. This discovery identifies hospital sewer biofilms as a more likely risk environment for resistance evolution than previously considered. More replication would be required to assess potential differences between hospitals. Clear morphological effects of HWW on *E. coli* within 1 h of exposure also suggest a possibility for effects on transient bacteria in hospital sewer pipes, although more studies on selective processes on planktonic bacteria in situ are needed.

Despite being the most used antibiotic class globally, β-lactams have previously not been widely recognised for environmental selection risks due to their relative instability. However, usage is certainly higher in hospitals than in community settings, and hospital sewers are typically located relatively close to toilets, leaving limited opportunities for β-lactam degradation. During this study, we learned that freezing samples before sterile filtration, as implemented in the 2024 sampling campaign, precluded reliable assessment of β-lactams. However, ciprofloxacin was unaffected; therefore, resistance selection was demonstrated by wastewater from all hospitals and, with appropriate sampling methodology, also linked to cefotaxime exposure at the Sahlgrenska University Hospital. The evidence for selection by cefotaxime broadly across all hospitals is therefore also weaker than for ciprofloxacin. The results presented here indicate the value of filtering wastewaters before freezing and to analyse samples without unnecessary delays to reduce risk of degrading β-lactams. The reason for the specific losses of β-lactams linked to freezing and thawing whole effluent is not yet known, but we speculate that it could be linked to cell lysis and the release of β-lactamases to the wastewater from indigenous resistant bacteria.

The identification of ciprofloxacin as an important driver of selection was expected. Fluoroquinolones are considerably more stable than β-lactams and have, based on measured concentrations and experimentally derived selective concentrations, been suggested as potential drivers of selection in several settings^5,26,45^. Both ciprofloxacin and cefotaxime individually select for multi-drug resistance, most likely through co-selection via the common co-occurrence of different resistance determinants in bacterial genomes. One should therefore be careful in deriving plausible drivers solely based on the resistances selected for.

Despite high use at the Sahlgrenska University Hospital (Supplementary Data 11 and 12), meropenem was only detected up to a few hundred ng/L in the wastewater. This is in agreement with several studies of hospital wastewater elsewhere^46–49^. In contrast, we have identified three studies reporting considerably higher concentrations of meropenem in hospital wastewaters, from low μg/L up to 1.9 mg/L^21,50,51^. Such high concentrations are very likely to be selective^50^. We analysed meropenem by LC-MS/MS in freshly filtered wastewater samples, stored only briefly at −80 °C^37^. We applied fresh deuterated meropenem as an internal standard to minimise potential matrix effects on retention time and signal suppression. To further validate the results, we also quantified a subset of the samples using the standard addition method and performed a freeze/thaw evaluation. We also evaluated the addition of EDTA to the sample and did direct injection without solid-phase extraction. All these approaches supported the initially determined concentration and had, at most, a very minor influence on the results (Supplementary Data 13). We can therefore currently not explain the discrepancy between studies, and for that reason, we do not rule out a contribution to the observed β-lactam selection pressure from meropenem, in addition to cefotaxime.

While HWW selects for resistant *E. coli* and *K. pneumoniae*, it is still unknown how far such effects extend taxonomically. Given the broad spectra of both ciprofloxacin and cefotaxime, selection is expected across a wide range of sewage bacteria, but certainly not all. Metagenomic analyses of wastewaters and biofilms may shed some light on whether broader selection takes place, although ARG-based assays cannot conclusively identify within-species selection unless ARGs can be conclusively linked to species^26,27^. The findings of very low relative ARG abundances in municipal effluent biofilms are consistent with (but not evidence for) deselection of resistant bacteria by many municipal wastewaters^17^. While quinolones and β-lactams are the only two classes strongly inhibiting growth of *E. coli* in the studied HWWs, it is possible that additional antibiotics and chemical stressors could be important selective agents in bacterial taxa with different susceptibility profiles^26^.

Based on the widespread selection at all investigated Swedish hospitals, and the established links to the usage and concentrations of ciprofloxacin and cefotaxime, it is highly plausible that wastewaters from other hospitals around the world select for antibiotic resistance as well^18,19,52^. Ciprofloxacin has for decades been one of the most used antibiotics worldwide. Concentrations above or close to the concentrations detected here have been reported in many HWWs^20,21^. The generation of local usage data here should, together with identified drivers, also allow for an initial assessment of risks for selection even at hospitals where there are no chemical analysis data of antibiotics in the wastewater. Hence, with similar or higher use of ciprofloxacin and/or cefotaxime, risks are apparent. While Sweden is low in total antibiotic usage, in-patient use at hospitals is comparable to other EU countries, with approximately a third of patients receiving antibiotic treatment at any given timepoint^18,19,52^. This apparent anomaly is, at least partly, explained by the fact that Sweden has the least hospital beds per capita in the EU^53^. Cefotaxime usage is typically higher in most large hospitals in Sweden compared to the studied ones in the Västra Götaland Region, including the Sahlgrenska University Hospital. Therefore, its importance may have been underestimated from a national perspective. Being injectable only, cefotaxime is almost exclusively used for inpatients at hospitals. While cefotaxime is a very common antibiotic, other third-generation cephalosporins, in particular ceftriaxone, are more often used in some countries^54^. This suggests a larger uncertainty with regard to how widespread a significant β-lactam selection pressure is in HWWs across the world, as well as to the relative attribution to different β-lactams.

Selection and evolution of resistance occurring within hospital sewers is a concern for the design of potential mitigations, as removing antibiotics further downstream at municipal treatment plants, where antibiotics are already diluted, may have limited effect. Also, minimising environmental release of and human exposure to resistant bacteria downstream is critical, but often challenging. This points to the value of mitigating environmental resistance selection in the sewers at hospitals, either by on-site wastewater treatment or by exploring upstream solutions, such as substitutions of fluoroquinolones and cefotaxime with other treatment options. In many places, including Sweden, there are already efforts in place to reduce the use of fluoroquinolone antibiotics. Also, as cefotaxime is a driver of recurrent healthcare facility-associated *Clostridioides difficile* infections^55^, there are initiatives to replace cefotaxime with alternative antibiotics, such as piperacillin-tazobactam when possible. This shows that some level of replacement to reduce selection risks in wastewater is likely feasible, aiming for mutually beneficial outcomes without compromising patient safety.

## Methods

### Wastewater collection

Hospital wastewater samples were collected from the central complex of the Sahlgrenska University Hospital in Gothenburg, the largest hospital in Sweden, with approximately 500 beds, with wards contributing sewage to the sampling point. Two 24-h composite samples, HWW1 and HWW2, were collected on January 7th and 21st, 2022, respectively, with eight complementary samples collected on 09Jan2025, 13May2025, 12Aug2025, 26Aug2025, 16Sep2025, 23Sep2025, 02Oct2025 and 08Oct2025. Composite samples were collected over each 24-h period (approximately 08:00-08:00), with approximately 31 mL sampled every 9 min (160 sub-samples per day), collected in refrigerated 5 L containers. Fresh samples were filtered sequentially (1.0, 0.45 and 0.22 µm pore size) to remove particles and bacteria. The sterility of each sample was confirmed by incubating 90% wastewater with 10% (v/v) lysogeny broth (LB) at 37 °C for 24 h (to mimic subsequent test conditions) and the optical density (OD) was measured at 600 nm. Filtered samples were stored in 50 mL falcon tube aliquots at −20 °C (2022 samples) and −80 °C (2025 samples) until further analyses.

The broader wastewater sampling of hospitals and wastewater treatment facilities in the Västra Götaland Region (Sahlgrenska, Östra, Mölndal, Kungälv, Södra Älvsborgs sjukhus (SÄS), Norra Älvsborgs Sjukhus (NÄL) and Skaraborgs Sjukhus (SkaS)) and Uppsala (Akademiska) followed a similar sampling protocol but with the critical difference that samples were frozen without prior filtering (filtered only after thawing). The approximate number of beds for each hospital contributing sewage to the sampling points can be found in Supplementary Data 14. Composite 24 h samples (approximately 08:00-08:00) were collected on a minimum of 4 different days between 01Sept2024 and 30Nov2024, with a minimum of 4 days between sampling days (Supplementary Data 9). One subsample of 160 mL from each site was mixed with 40 mL 100% glycerol (final concentration 20%) and stored at −20 °C for cultivation-based analyses (no filtering) and DNA extraction for sequencing. Additionally, municipal wastewater treatment plants receiving untreated wastewater from these hospitals were sampled during the same period (Supplementary Data 9). Figure 1 shows the locations of both hospitals and municipal sites. Ryaverket receives wastewater from four hospitals (Sahlgrenska, Östra, Mölndal and Kungälv). Figure 1 was made using R (v 4.4.0) with packages sf (v, 1.1.0), dplyr (v. 1.1.4), ggplot2 (v. 4.0.0) and ggrepel (v. 0.9.6).

Composite samples (24 h) from two Stockholm hospitals (Södersjukhuset and Huddinge Sjukhus) and their corresponding municipal wastewater treatment plant (two influent and one effluent stream) were collected similarly as the broad sampling campaign above in December of 2022 and February of 2023.

### Antibiotics usage data

The Region Västra Götaland kindly provided register data on each administered antibiotic dose, both orally and intravenously, from all wards contributing to wastewater at each hospital wastewater sampling point (aggregated data only). Neonatal wards were excluded as urine and faeces are consistently collected in diapers. Emergency departments were also excluded because of a different system for registering use. Usage data was collected for each of the 3 days preceding each of the wastewater sampling dates, acknowledging that time from administration to excretion via urine and faeces will vary both between antibiotics and between patients.

### Antibiotic concentrations in collected HWW

Based on the usage data, we selected antibiotics for quantification with the intent to identify which antibiotics in the HWW could potentially cause selection as described in the ‘Results’ section. Antibiotics were analysed in triplicate using liquid chromatography (LC) coupled with triple quadrupole mass spectrometry (MS)^16^. Specifically, LC-MS grade methanol and acetonitrile (Lichrosolv—hypergrade) were used, and purified water was prepared using a Milli-Q Advantage system. The mobile chromatographic phases were prepared using formic acid (Sigma-Aldrich, Steinheim, Germany) at a concentration of 0.1%. All standards, including isotope-labelled standards, are listed in Supplementary Data 15. Prior to solid phase extraction and measurement by LC-MS, samples were thawed and spiked with 5 ng of each internal labelled and surrogate standard. The LC-MS analysis was conducted on a UHPLC system connected to a TSQ Quantitative triple quadrupole MS (Thermo Scientific, San Jose, USA) equipped with a heated electrospray ionisation ion source operating in positive mode. The MS settings used for the included chemicals are presented in Supplementary Data 15.

### Selection experiment with wastewater and individual antibiotics in complex communities

To assess the selectivity of wastewater or individual antibiotics, we employed a previously described method using municipal wastewater microbial communities (*n* = 3) as inocula^17,28^. Notably, it is a selection-potential assay, not a reconstruction of sewer ecology. In brief, at the beginning of each selection experiment, a frozen batch of one inoculum was thawed at room temperature. Then, 0.1 mL of aliquots were inoculated into 15 mL falcon tubes containing 4.9 mL of a mixture consisting of 90% sterile-filtered wastewater (or physiological saline as control) and 10% LB. The tubes were incubated at 37 °C and 170 rpm for 24 h. Five microliters of these cultures were serially passaged daily over 72 h to a fresh tube containing the same ratio of wastewater (or physiological saline) and LB as described above. The selection potential of individual antibiotics was assessed using the same setup. Antibiotics (Sigma Aldrich; Sweden) were tested at concentrations measured in HWW and at breakpoint concentrations.

At the start of each experiment (0 h saline control) as well as after three passages, aliquots were cultured on antibiotic/non-antibiotic plates. To quantify resistant and non-resistant *E. coli*, CHROMagar™ ECC plates (CHROMagar™, France) were used with and without supplemented antibiotics at EUCAST 2022 clinical breakpoints (8 mg/L and 2 mg/L of amoxicillin and clavulanic acid (AMC); 4 mg/L of ceftazidime (CAZ); 16 mg/L of cefadroxil (CDR); 0.5 mg/L of ciprofloxacin (CIP); 4 mg/L and 76 mg/L of trimethoprim-sulfamethoxazole (SXT), respectively; 2 mg/L of tobramycin (TOB); and 8 mg/L and 4 mg/L of piperacillin and tazobactam, respectively; 8 mg/L of piperacillin (PIP)). These antibiotics represent several major classes of antibiotics frequently used in hospitals, all with activity against *E. coli*. Also, resistance to all of these antibiotics is present in *E. coli* in wastewater communities. It was therefore considered plausible that resistance to these classes could be selected for, either directly or indirectly (co-selection by other antibiotics). For the broad sampling campaign, including analyses of Stockholm samples (ten hospitals), only resistance to CIP (0.5 mg/L) and CTX (2 mg/L) was assessed. For *K. pneumoniae*, the agar plates were Simmons Citrate agar, pH 7.2 plus inositol (SCAI) plates (CHROMagar™, France), and SCAI supplemented with the same antibiotics at clinical breakpoints as specified for *E. coli*. The CHROMagar™ ECC plates were incubated for 20–24 h at 37 °C, and typical blue colonies (*E. coli*) were counted and reported as CFU/mL. The SCAI plates were incubated for 44–48 h at 42 °C, and typical yellow colonies were counted and reported as CFU/mL. Given that SCAI plates are less specific for *K. pneumoniae* than CHROMagar™ is for *E. coli*^16,56^, randomly selected colonies counted on each SCAI plate were confirmed as *K. pneumoniae* using matrix-assisted laser desorption/ionisation time of flight (MALDI-TOF MS). The resistance ratio was calculated by determining the number of colonies on antibiotic-containing plates (CFU/mL) relative to control plates without antibiotics. The percentage of resistance was calculated by multiplying this ratio by 100. One-way analysis of variance (ANOVA) with Dunnett’s multiple comparisons test was used to compare the wastewaters with physiological saline obtained at 0 h and 72 h, with each resistance type investigated separately. The response variable in the ANOVA was the *log*_10_-transformed percentage of resistance incremented by 1, to account for cases where the number of colonies on antibiotic-containing could be 0. One-way ANOVA with Dunnett’s multiple comparisons test was performed in GraphPad Prism (v10.1.0(316)).

To reach beyond classification of percentage of resistance above/below clinical breakpoints, and to assess if multi-resistant strains were selected for, disc diffusion tests were performed for fifty-two *K. pneumoniae* isolates (26 from 0 h and 26 from 72 h) that were randomly picked from SCAI plates. Overnight cultures were prepared for each isolate, and a solution corresponding to 0.5 McFarland standard was spread on Mueller–Hinton agar (MHA). Antibiotic discs (Oxoid, Sweden) were then placed on the agar plates, which were subsequently incubated at 37 °C for 16 h. The diameter of the inhibition zone around each disc was measured, and the results were interpreted according to the European Committee on Antimicrobial Susceptibility Testing (EUCAST 2022) guidelines.

### Growth of individual *K. pneumoniae* isolates in HWW

To further enable comparisons of individual strains' level of sensitivity to antibiotics with their potential for growth in hospital wastewater (HWW2), a collection of wastewater *K. pneumoniae* isolates (*n* = 117) with diverse resistance profiles was studied (Supplementary Data 7). The antibiotic susceptibility of each strain was evaluated using the Etest® method. The strips (BioMérieux, Marcy-l’Etoile, France) included amoxicillin/clavulanic acid (256 µg/mL), azithromycin (256 µg/mL), aztreonam (256 µg/mL), cefotaxime (32 µg/mL), ceftazidime (256 µg/mL), ceftriaxone (256 µg/mL), cefuroxime (256 µg/mL), cefepime (256 µg/mL), chloramphenicol (256 µg/mL), ciprofloxacin (32 µg/mL), clarithromycin (256 µg/mL), doripenem (32 µg/mL), gentamicin (256 µg/mL), imipenem (32 µg/mL), meropenem (32 µg/mL), nalidixic acid (256 µg/mL), oxacillin (256 µg/mL), piperacillin (256 µg/mL), tetracycline (256 µg/mL), and tobramycin (256 µg/mL). Overnight culture of each strain was diluted to 0.5 McFarland standard in physiological saline and spread onto a MHA plate. The Etest® strips were then placed on the plate and incubated at 37 °C for 16–20 h to determine MICs.

Individual growth curves were generated in triplicate for each isolate in HWW2 using the OmniLog™ (Biolog, USA) system in 96-well plates. Briefly, 18 mL of sterile filtered wastewater or saline was combined with 2 mL of LB medium and 200 µL of Biolog redox dye A per plate. The dye changes from yellow to purple as cells convert extracellular metabolites into mitochondrial reducing equivalents. After 200 µL of this mixture was added to every well, 2.5 µL of an overnight culture of each strain was inoculated into every second well. The remaining wells served as contamination controls. The plates were incubated at 37 °C with images taken every 15 min for 24 h. Total growth, measured as area under the curve over 24 h, was based on the median of three technical replicates.

### Construction of isogenic resistant *E. coli* strains

Experiments with isogenic strains that only differ by one or a few defined resistance genes can provide strong evidence for selection pressures caused by the type of antibiotics to which those genes provide resistance. We therefore created a set of isogenic plasmids to test the growth-inhibiting effects of fluoroquinolones and β-lactams in *E. coli*. The plasmid vector pZE21, containing a kanamycin resistance marker, was chosen as the template for the plasmid construction. The plasmid was linearized using PCR with primers that inversely flanked the multiple cloning site (MCS) region to create complementary ends for precise insertion of the genes of interest (GOIs) using Gibson Assembly. Since the GOIs contain their own promoters and terminators, the MCS was intentionally removed during linearization to prevent interference with gene expression.

The selected antibiotic resistance genes were *bla*NDM-1 (CP145538.1) (resistance to essentially all β-lactams) and *qnrB* (CP039327.1) (resistance to fluoroquinolones). Each gene sequence was modified in silico by adding the *recA* (∆LexA) promoter 16 bp upstream of the start codon and a *rrnG* terminator 12 bp downstream of the stop codon. Additional nucleotides were added flanking these regions to account for Gibson Assembly complementary sites. The 5′- and 3′-prime ends of the *bla*NDM-1 and *qnrB* constructs were designed with unique complementary ends for directional and multiplexed Gibson Assembly. The *qnrB* gene’s upstream sequence was complementary to one end of the linear plasmid, the downstream end was complementary to the upstream end of the *bla*NDM-1 construct, and the downstream end of the *bla*NDM-1 construct was complementary to the other end of the linear plasmid. The overlapping regions were 15 bp in length with a melting temperature of 50 °C, following the Gibson Assembly® Cloning Kit (New England Biolabs, USA) protocol. The complementary regions were designed and tested in silico using the SnapGene Software (Version 7.1.1), as well as all primers used in this study (Supplementary Data 16). These constructs were created through de novo DNA synthesis by Twist Bioscience, USA.

The linearization of pZE21 plasmid and the amplification of synthesised *qnrB* and *bla*NDM-1 genes were performed using PhusionTM High-fidelity DNA polymerase (ThermoFisher, USA) and primers listed in Supplementary Data 16. PCR products were purified using AmpureXP beads (Beckman Coulter, USA) following the manufacturer’s instructions.

A Gibson assembly reaction was performed to circulate pZE21 plasmid (150 ng) with *qnrB* (100 ng) and *bla*NDM-1 (100 ng) genes in a total volume of 20 µL. The mixture was incubated at 50 °C for 30 min. Assembled plasmid (pNR-11) was then electroporated into *E. coli* TOP10 and selected on LB agar containing kanamycin 50 mg/L. The *qnrB* and *bla*NDM-1 genes were respectively removed from pNR-11 plasmid (generating plasmids pNR-01 and pNR-10) by PCR using primers Con2_F & pZE21_LR and pZE21_LF & Con1_R (Supplementary Data 16). The resulting linearized plasmids were 5′-phosphorylated using T4 kinase (Thermo Scientific) at 37 °C for 20 min before circulated with T4 ligase (New England Biolabs) at 16 °C overnight. The plasmids were then transformed into *E. coli* TOP10 and selected on LB containing kanamycin 50 mg/L. The plasmid sizes and inserted genes were confirmed by PCR using corresponding primers (Supplementary Data 16).

All desired plasmids were extracted from *E. coli* TOP10 using GeneJET plasmid miniprep kit (Thermofisher, USA) by following the manufacturer’s instructions and then finally transformed into an environmental *E. coli* strain (MC221) that was largely antibiotic-sensitive (Supplementary Data 4 and 5) and not able to grow in HWW.

All constructed strains as well as the background strain were investigated for their antibiotic resistance profile by disc diffusion test using the procedure of the CLSI against ciprofloxacin (5 µg), cefotaxime (30 µg), meropenem (5 µg), mecillinam (10 µg), amoxicillin (10 µg), piperacillin (30 µg), amikacin (30 µg), gentamicin (10 µg), tobramycin (10 µg), trimethoprim (5 µg), chloramphenicol (30 µg) and doxycycline (30 µg) (Supplementary Data 4). Additionally, the MICs of tobramycin, ciprofloxacin, cefotaxime and meropenem were investigated by broth microdilution (Supplementary Data 5). Reference strain *E. coli* ATCC 25922 was used to verify the quality of the testing procedures.

The collection of isogenic strains was subsequently tested for their growth in the presence of HWW using the OmniLog^TM^-setup as described above under section ‘Growth of individual *K. pneumoniae* isolates in HWW’.

### Generation of ciprofloxacin-resistant *K. pneumoniae* mutants

As an alternative way of testing the hypothesis that only ciprofloxacin resistance was necessary for growth in HWW, non-growing strains of *K. pneumoniae* were selected and made resistant to ciprofloxacin by increasing their MIC. Briefly, these strains were initially cultured in Mueller–Hinton broth overnight. Subsequently, 5 µL of the initial culture was transferred to Mueller–Hinton broth containing increasing concentrations of ciprofloxacin (0.008, 0.016, and 0.03 mg/L) after every 24 h. The bacteria were then plated on SCAI plates supplemented with 0.02 mg/L of ciprofloxacin after exposure to 0.03 mg/L. Mutant strains were then isolated, and their growth curves in LB and HWW using the Omnilog™ machine were generated as described in section ‘Growth of individual *K. pneumoniae* isolates in HWW’.

### Morphological changes assessed by microscopy analysis

All microscopy was performed on a Nikon Eclipse Ti2 equipped with a CFI Plan Apochromat DM Lambda 100× Oil objective (N.A. 1.45, W.D. 0.13 mm), a Photometrics PRIME BSI camera, a Lumencor Sola SE II FISH 365 light source, an Okolab temperature incubation chamber, and NIS-Elements AR software version 5.21.03. Images were analysed with ImageJ^57^ and MicrobeJ^58^. Experiments were performed in a minimum of three replicates for all control conditions and a minimum of two replicates for wastewater samples.

Phenotypic analysis experiments were performed using *E. coli* HS524 (MG1655 *mreB::mreB-msfgfp kan*)^59^ (MreB localization), and *E. coli* RC1 (*Δlac Δara ΔminCDE*) carrying plasmid pFX9 (*bla Plac-gfp-minD minE)*^36^ (MinD oscillation). All *E. coli* strains were aerobically grown in LB at 37 °C and stress experiments were performed in early log phase (OD_600_ 0.2–0.4). Antibiotics were added at the following concentrations: 0.5 µg/mL ciprofloxacin, 0.012, 0.02, and 2 µg/mL cefotaxime, and 100 µM CCCP. For exposure to wastewater samples, cells were pelleted (1 min, 16 000 × *g*, 37 °C) and resuspended in filtered effluent samples. For MreB localization, *E. coli* HS524 was grown until an OD_600_ of 0.3–0.4 and treated with antibiotics and wastewater samples as specified above. Images were taken after 30 min of treatment. For MinD oscillation, *E. coli* RC1 pFX9 was grown until an OD_600_ of 0.2 followed by addition of 10 µM isopropyl β-d-1-thiogalactopyranoside (IPTG) to induce the expression of GFP-MinD. After 30 min, antibiotics were added as specified above. For wastewater samples, 10 µM IPTG was re-added after resuspension in effluent. After 30 min of antibiotic treatment, time-lapse microscopy was performed, recording images every 15 s for 2 min. Image stacks were converted into videos using ImageJ.

### DNA extraction and sequencing

#### DNA extractions

Sixteen biofilm samples (eight hospital and eight municipal sources) and 83 wastewater samples (51 hospital and 32 municipal sources were selected from the Västra Götaland County and Uppsala City sampling; Supplementary Data 17). Too small volumes of treated municipal effluent were unfortunately collected to generate useful amounts of DNA. Aliquots from each wastewater and biofilm sample were taken, 40 mL and 20 mL respectively, and frozen at −20 °C. The samples were thawed at room temperature and centrifuged at 16,100 × *g* for 1 h using a 5810R Centrifuge (Eppendorf, Germany). DNA was extracted from the collected particulate samples using an in-house modified chloroform-isoamyl alcohol and alcohol-salt precipitation protocol (Supplementary Methods). Samples resulting in DNA concentrations >10 ng/µl and yielding at least 500 ng were considered successful.

#### Shotgun metagenomic sequencing

Shotgun metagenomics sequencing using a NovaSeqXPlus was outsourced to NGI Sweden, a division of SciLifeLab located in Stockholm, using an ISO/IEC 17025-accredited workflow (Supplementary Methods). During sequencing, the hospital wastewater sample NÄL 10/7/2024 was lost and therefore excluded from further analysis. Moreover, the hospital biofilm sample from Östra was recognised as a compromised sample (hanging above the water surface) and therefore also excluded from the study.

Sequencing resulted in an average of 231 million reads per sample (SD = 41 million reads, combined forward and reverse) after quality filtering. The overall quality of the sequencing reads was high, boasting a Phred score larger than 35 for all samples. Therefore, the outcomes of the sequencing produced reads that were appropriate for further downstream analysis.

#### Metagenomic data analysis

Analyses were hosted and executed on our in-house server running Red Hat Enterprise Linux (v7.9) consisting of 80 Intel(R) Xeon(R) Gold 6230 CPU @ 2.10 GHz CPUs and 754 G RAM. All scripting and data processing was mediated through python programming (v3.9.15) and the software environments were maintained using Anaconda (v21.48.22.159-1). Descriptive and interferential statistics on the outcomes of the Diamond and Sylph software processing were done using the R Statistical Software (v4.5.1) and RStudio interactive development environment (v2025.05.1, Build 513). All descriptive plots were generated using ggplot2 (v3.5.2).

A custom workflow was used to mediate metagenomic data processing (Suppl Methods). Briefly, quality assessments before and after quality trimming were done using FastQC (v0.11.9) and summarised using MultiQC (v1.14). Sequencing read quality trimming was performed using BBMap (v39.01). Diamond BLAST was used to estimate the abundance of ARGs using the ResFinder database (v2.0.2)^60^. To account for variations in sequencing depth, all Diamond counts were normalized as counts per million reads.

Microbial community composition was determined using the Sylph (v0.8.1) metagenomic profiling software^61^ and their proprietary GTDB_r226 reference database. Beta-diversity was calculated on genus level to determine the taxonomic variations and similarities between samples. This was done using the vegan package (v2.7-2) and the *vegdist* function. Principal Coordinates Analysis (PCoA) based on Bray–Curtis dissimilarities was performed using classical multidimensional scaling to generate two-dimensional coordinates for visualising beta-diversity differences between samples.

In all cases, the overall distribution of data was not considered normal (*p* < 0.05) as assessed by the Shapiro–Wilk and Anderson–Darling tests. Therefore, non-parametric statistical tests were used to determine significance between groups. For two independent groups, the Mann–Whitney U test was used; for multiple group comparisons, the Kruskal–Wallis test was used. Significance was recognised for *p*-values < 0.05. In the event of a significant result within groups, the Dunn’s test post-hoc analysis was used.

### Comparing degrees of resistance in *E. coli* and ARG counts between wastewaters and biofilms

From the wastewater samples (September–November 2024) stored in 20% glycerol, a small subsample was serially diluted and plated on CHROMagar™ ECC plates (CHROMagar™, France) and on CHROMagar™ ECC supplemented with antibiotics (ciprofloxacin and cefotaxime) at clinical breakpoints (0.5 mg/L and 2 mg/L respectively), generating CFU/mL. This was done in quadruplicate for each location, providing technical replication, while each date, a minimum of 3 per location, was a biological replicate. The volume plated for each dilution was always 100 µl. A similar methodology was performed for biofilms; however, before taking a small subsample, the biofilms were broken up through vortexing for at least 1 min with glass beads. Thus, the detection limit of *E. coli was* 1 CFU per 100 µl of resuspended biofilm sample or serially diluted wastewater sample. Additionally, the hospital biofilms were plated 16 times each, as opposed to in quadruplicate, providing more technical replication. Notably, since there was only one biofilm collected per location, there was no biological replication, only technical replication of the biofilm analyses. The median CFU/ml counts of the technical replicates were then used as input to calculate the resistance rates of *E. coli* (by dividing the counts on antibiotic-supplemented plates by the counts from non-antibiotic plates) for all samples and to perform statistical tests.

To test for significant differences in resistance rates in *E. coli* between biofilms and waters from hospital and municipal influent sites, negative binomial mixed-effects models were used. For each antibiotic separately, the median CFU/ml from the technical replicates on the antibiotic-containing plates was used as a response variable, while the median CFU/mL from the corresponding non-antibiotic plates was used as an offset. Random intercepts for each site and sample ID (unique identifier for a biological replicate) were also included. This use of an offset allows modelling of a resistance rate (resistant *E. coli* counts vs total *E. coli* counts), while the random intercepts account for clustering/relatedness among samples (i.e. that resistance rates in biofilms and in waters from the same site may be correlated). One overall model per antibiotic was fitted, where matrix (wastewater vs. biofilm), location type (hospital vs. municipal influent), and their interaction were used as fixed effects. From these models, the contrast across the matrix for the two location types was estimated and tested for significance using a two-sided Wald *z*-test. This would correspond to testing the log ratio of resistance rates in hospital wastewaters vs hospital biofilms or municipal influent wastewaters vs municipal influent biofilms. A significant, positive value would indicate enrichment of resistance in the biofilm compared to the wastewater for the given location type. Similarly, the contrast across location type for the two matrix types was estimated and tested for significance using a two-sided Wald *z*-test. This would correspond to testing the log ratio of resistance rates in hospital vs municipal influent biofilms, or hospital vs municipal influent wastewaters. A significant, positive value would indicate enrichment of resistance in the hospital compared to municipal influent for the given matrix type. For the Wald *z*-tests, Bonferroni correction was applied to the resulting *p*-values to account for the multiple comparisons being made: two comparisons for contrasts across matrix and two comparisons for contrasts across location type. Also, tests for the significance of the interaction term, which would indicate differences in changes in resistance rates between biofilms and wastewaters across the two location types, were performed via a likelihood ratio test comparing the fitted model with a simplified model fitted without the interaction term.

As a complement to the analysis based on *E. coli*, a similar analysis was performed based on the total ARG counts from the metagenomic sequencing data (Section ‘DNA extraction and sequencing’). Here, the total ARG count was used as a response variable, while the total million reads were used as an offset in a negative binomial mixed-effects model, with random intercepts for each site. This use of an offset means that what is effectively modelled is the ARG counts per million reads, while the random intercepts account for relatedness among samples (i.e. how the ARG carriage rates in biofilms and wastewaters may be correlated within sites). As for the *E. coli*-based analysis, one overall model with location type, matrix and their interaction as fixed effects was fitted and the significance of the interaction term was tested. Then, the contrast across matrix was computed and tested for significance using a two-sided Wald *z*-test for both location types. This tests the estimated log ratio of total ARG counts per million reads between biofilms and wastewaters. A significant, positive value would indicate higher ARG carriage rates in biofilms than wastewater for the given location type. Bonferroni correction was applied to the *p*-values to account for the multiple comparisons being made.

All statistical analysis were performed in R (v.4.4). All negative binomial mixed effects models were fitted using the R package glmmTMB (v.1.1.14) and corresponding contrast and hypothesis test calculations were performed using the emmeans (v.2.0.1) package. Residuals were checked using DHARMa (v.0.4.7).

### Reporting summary

Further information on research design is available in the Nature Portfolio Reporting Summary linked to this article.

## Supplementary information

Supplementary Information

Description of Additional Supplementary Files

Supplementary Data 1 - 17

Supplementary Video 1 pFX9_untreated 30 min_002-1

Supplementary Video 2 pFX9_CCCP 30 min_006-1

Supplementary Video 3 pFX9 HWW1 30 min_029

Supplementary Video 4 FX9 HWW2 30 min_031

Reporting Summary

Transparent Peer Review file

## Source data

Source Data

## Data Availability

The sequencing data generated in this study has been deposited at NCBI repository under BioProject PRJNA1426335 (https://www.ncbi.nlm.nih.gov/bioproject/PRJNA1426335/), with sequences available under accession numbers SAMN55849194–SAMN55849290. Other data generated in this study are provided in the article, in the Supplementary Tables or the Source Data. Source data are provided with this paper.

## Code availability

The code used for the processing of and statistical analysis of metagenomics data, and statistical analysis of resistance to ciprofloxacin and cefotaxime in *E. coli*, has been submitted to Github at: https://github.com/watNoel/VGRegion-Hospital-Wastewater-Selection-Project.
