# Supplementary material for: Widespread selection of antibiotic resistance within hospital sewers
Source: Nat Commun. 2026 Oct 29;17:11162. doi: 10.1038/s41467-026-78836-8 (PMC13720167; doi:10.1038/s41467-026-78836-8)
Supplement: Supplementary file 1 [file 41467_2026_78836_MOESM1_ESM.pdf]

Supplementary Information: Widespread selection of antibiotic resistance within hospital sewers

Declan A. Gray[1,2] • Nguyen Thi Nhung[1,2] • Roelof Dirk Coertze[1,2] • Mutshiene Deogratias Ekwanzala[1,2] • Noel Waters[1,2] • Josef D. Järhult[3] • Michaela Wenzel[1,4] • Christina Åhrén[1,2,5] • Jerker Fick[6] • Carl-Fredrik Flach[1,2] • D. G. Joakim Larsson[1,2*]

[1]Centre for Antibiotic Resistance Research in Gothenburg (CARe), University of Gothenburg, Gothenburg, Sweden

[2]Department of Infectious Diseases, Institute of Biomedicine, The Sahlgrenska Academy, University of Gothenburg, Gothenburg, Sweden

[3]Department of Medical Sciences; Zoonosis Science Center, Uppsala University, Sweden

[4]Division of Molecular Bioscience, Department of Life Sciences, Chalmers University of Technology, Sweden

[5]Swedish Strategic Program Against Antimicrobial Resistance (Strama), Region Västra Götaland, Gothenburg, Sweden

[6]Department of Chemistry, Umeå University, Sweden

✉ D.G.J. Larsson, E-mail: joakim.larsson@fysiologi.gu.se

Results

For *K. pneumoniae,* significant selection was observed for resistance to three antibiotics after exposure to HWW1, while resistance to one additional antibiotic (ciprofloxacin) was selected for after exposure to HWW2 (Supplementary Fig. 3), in line with higher concentrations of ciprofloxacin in HWW2 (Supplementary Data 3). Isolates with either 0 or 1 resistance were no longer present after exposure to HWW, and the mean number of resistances changed from 2.35 to 4.27 ($p = 0.0002306$, Wilcoxon rank sum test) (Supplementary Fig. 3D). Additional information on the resistance profiles of all 52 randomly selected *K. pneumoniae* isolates can be found in Supplementary Data 6. To confirm that the starting colonies (wastewater communities) as well as counted colonies at 72 h after exposure were indeed *K. pneumoniae* colonies, a selected number of counted colonies at 0 h ($n = 144$) and 72 h ($n = 140$) were analysed by MALDI-TOF MS (Biomérieux Vitek MS prime), showing a 93.0% and 89.3% specificity before and after exposure to wastewater, respectively (Supplementary Fig. 4).

For *K. pneumoniae*, 0.01 mg/L of ciprofloxacin selected for resistance to cefadroxil and piperacillin in natural communities, while exposure to the slightly higher ciprofloxacin concentration measured in HWW2 (0.0185 mg/L) resulted in additional selection for resistance to amoxicillin-clavulanic acid and ciprofloxacin (Supplementary Fig. 3). At the clinical breakpoint concentration, ciprofloxacin selected for amoxicillin-clavulanic acid, cefadroxil, ciprofloxacin, piperacillin and tobramycin resistance. Cefotaxime (0.0125 mg/L) selected significantly for resistance to trimethoprem-sulfamethoxazole and piperacillin. A similar trend was observed for amoxicillin-clavulanic acid, ceftazidime, and cefadroxil (Supplementary Fig. 3). At the clinical breakpoint concentration, cefotaxime selected for resistance to cefadroxil, trimethoprem-sulfamethoxazole, and piperacillin. MALDI-TOF MS of random colonies was used to confirm correct species identification of the great majority of the counted colonies after exposure of the wastewater community to 0.0185 mg/L of ciprofloxacin (90.9%) and 0.0125 mg/L of cefotaxime (89.2%) (Supplementary Fig. 4).

No isogenic strains with variable resistance phenotypes were constructed in *K. pneumoniae.* Instead, ciprofloxacin-resistant mutants were generated through passaging in increasing concentrations of ciprofloxacin. Out of 117 tested *K. pneumoniae* wastewater isolates, the great majority were inhibited by the hospital wastewater (Supplementary Fig. 8 A-H), still only six strains did not grow at all (not reaching 50 Omnilog units within 24 h). When exposing ciprofloxacin-resistant mutants of these six strains, growth in hospital wastewater was restored for all (Supplementary Fig. 8 I-L). This indicates that the strongest driver of resistance selection in *K. pneumoniae* in the investigated wastewater was ciprofloxacin, in line with the selection results after exposure to measured concentrations of antibiotics (Supplementary Fig. 3).

Given recent literature reporting very different concentrations of meropenem in hospital wastewater, we performed additional quality assurance and quality control for the meropenem analyses, including evaluating the possible influence of differences in protocols applied by Voigt *et al*[1,2] and Schuster *et al*[3], in relation to ours. This included concentration-addition experiments evaluating the role of freezing samples, addition of EDTA, direct injection versus prior solid phase extraction and the use of different qualifying ions. Regardless of method, concentration-additions gave very good and consistent results (some additional losses when adding EDTA) (Supplementary Data 13). Hence, we can currently not attribute the lower levels of meropenem detected here as compared to Voight *et al*[2] and Shuster *et al*[3] to differences in the analytical procedures.

Figures and captions

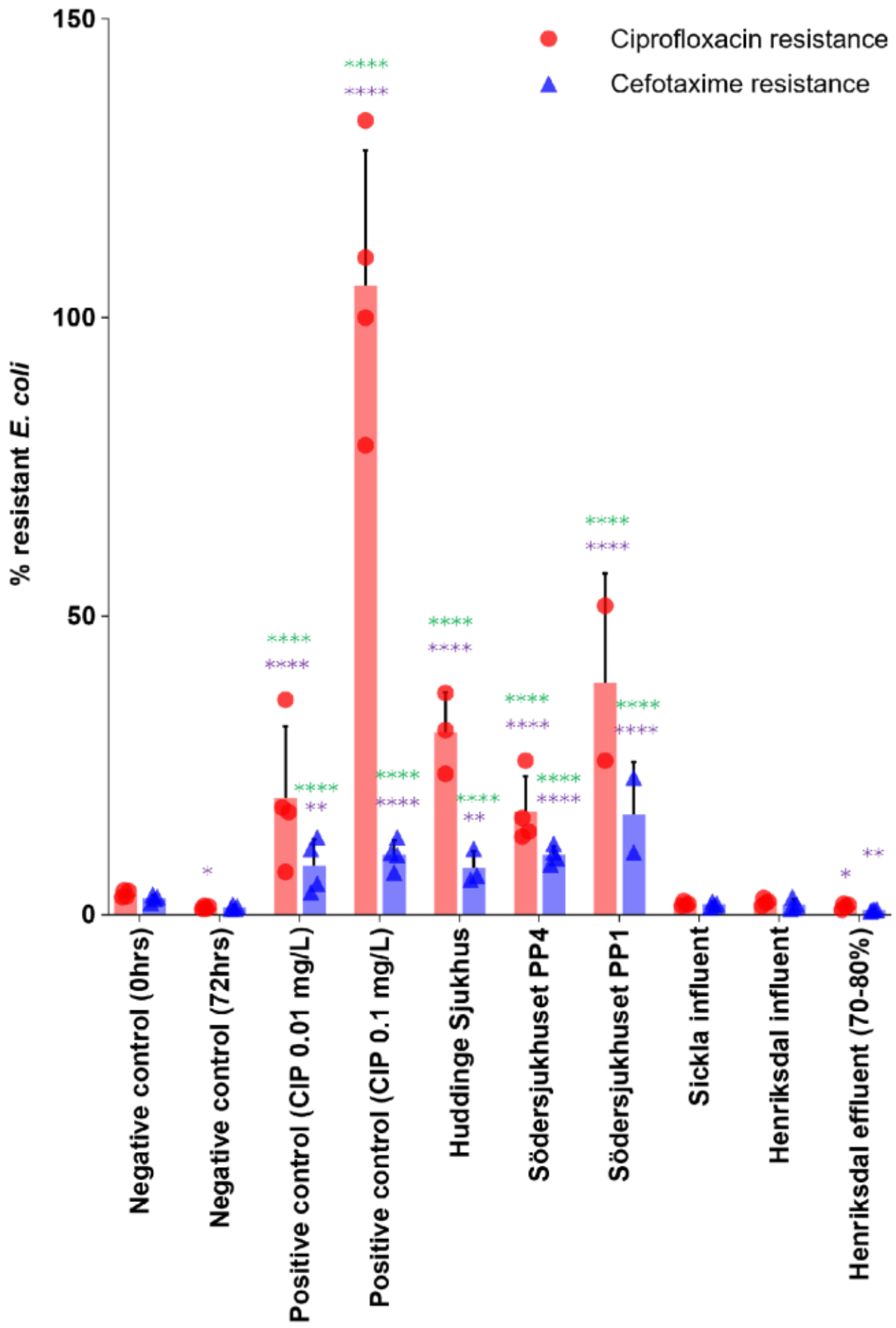

Supplementary Figure 1. Selection of antibiotic-resistant *E. coli* by in serially passaged (3x24 h) wastewater communities by sterile-filtered wastewaters (n = 4) (90%) from two large hospitals in Stockholm, Sweden and from their corresponding receiving municipal treatment plant. Physiological saline served as a negative control (0 h and 72 h), while 0.01 and 0.1 mg/L ciprofloxacin were positive controls. The value of applying a control parallel in time (72 h) and a control representing the original resistance rate is explained in Yu et al (2025)[4]. All cultures contained 10% LB medium. The percentage of resistant *E. coli* was determined by plating a fraction of the culture on CHROMagar™ ECC agar media with or without antibiotics. Antibiotics within agar plates were ciprofloxacin or cefotaxime at breakpoint concentrations (0.5 mg/L and 2 mg/L respectively). One-way ANOVA followed by Dunnett's multiple comparisons test was used to compare log-transformed resistance ratios of 72 h data to physiological saline at 0 or 72 h as two alternative controls (control 0 h purple * and control 72 h green *), (*p < 0.05; **p < 0.01, ***p < 0.001, ****p< 0.0001). Data are presented as mean values +/- SEM. All data and p-values associated with this figure are provided in the Source Data file. Sickla inlet and Henriksdal inlet both feed into Henriksdal WWTP, Henriksdal inlet

primarily receives water from the central parts of Stockholm municipality and parts of Nacka, while Sickla inlet receives water from the southern parts of Stockholm as well as Huddinge and parts of Haninge. 70-80% of the treated wastewater passes through the Henriksdal outlet. Abbreviations: CIP: ciprofloxacin. PP: sampling point.

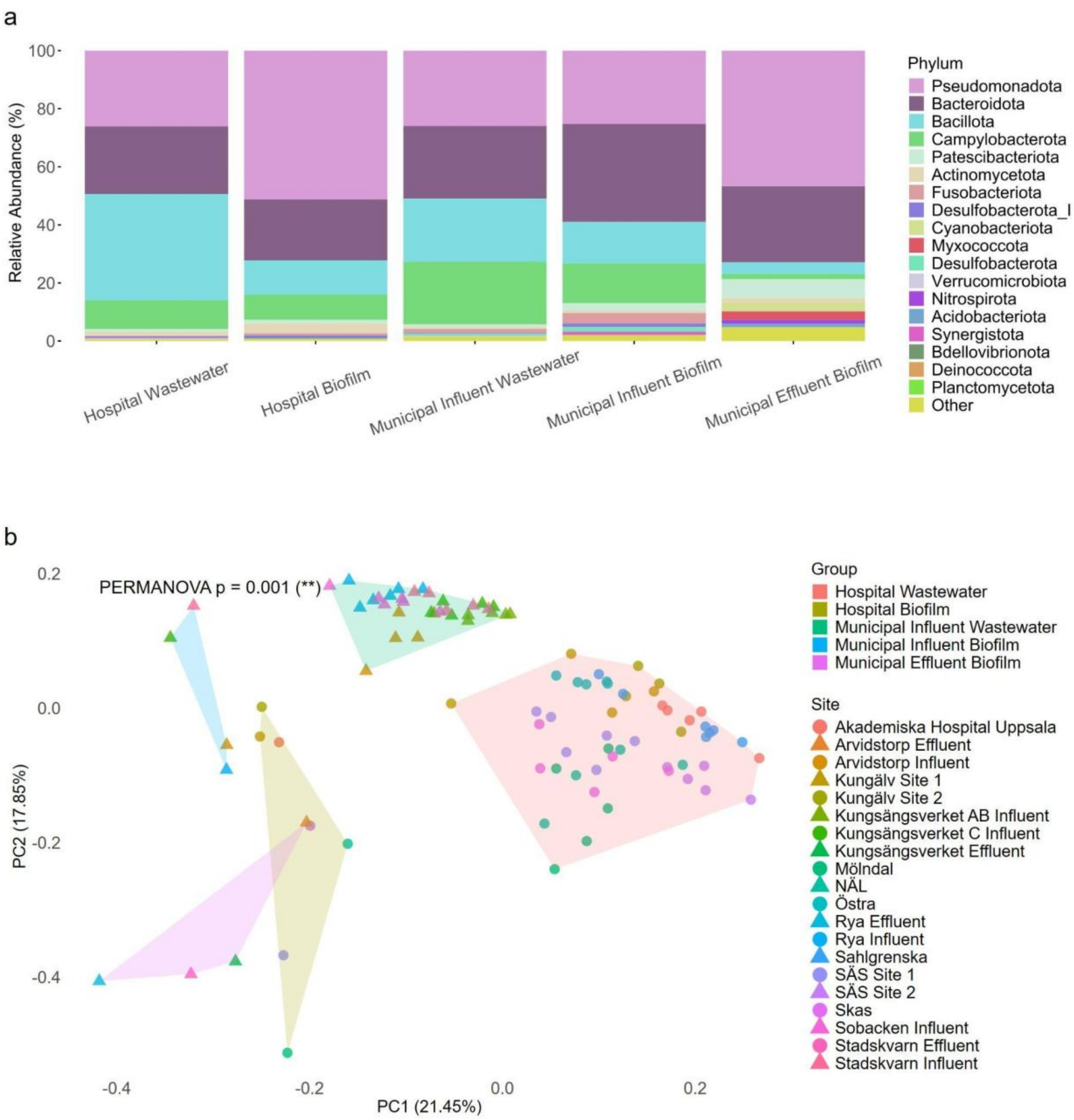

Supplementary Figure 2. Metagenomic analysis of biofilms and wastewaters from hospitals and associated municipal sewage treatment plants, complementing Fig. 4 in the main article. Panel a: Top 15 Phyla for each sample group. Panel b: Principal Coordinate Analysis illustrating beta-diversity on genus level of all samples by sample types (hospital = circles and municipal = triangles) and groups represented by coloured areas.

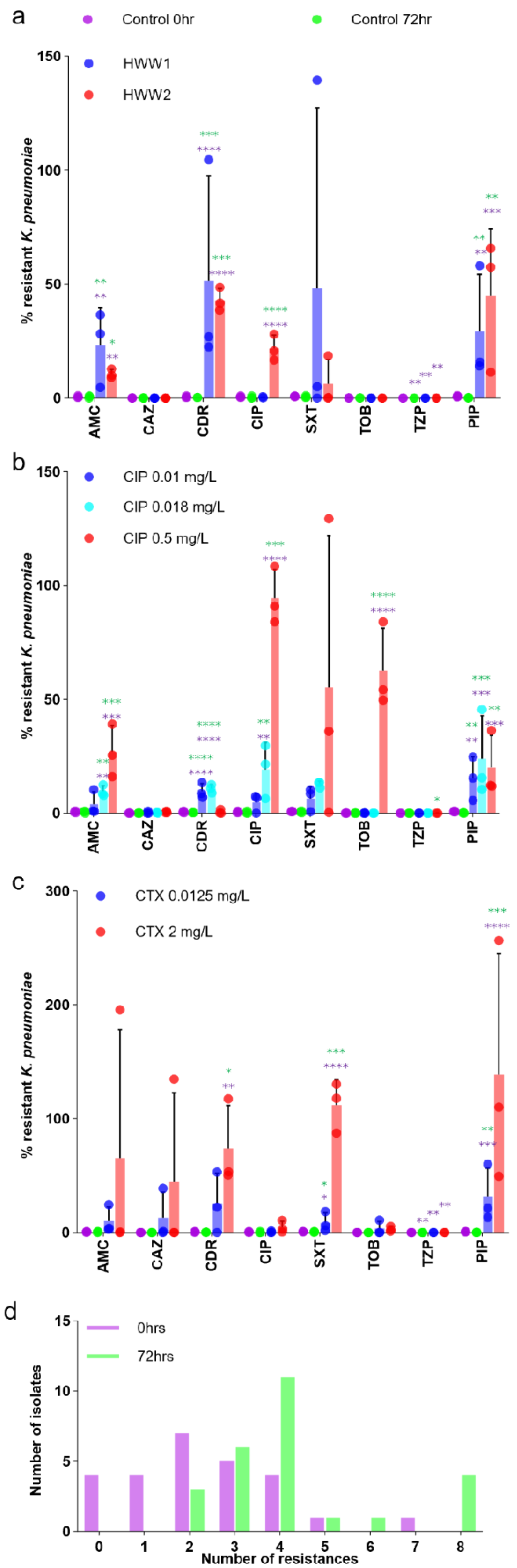

a
Control 0hr
Control 72hr
HWW1
HWW2
% resistant K. pneumoniae
AMC
CAZ
CDR
CIP
SXT
TOB
TZP
PIP
b
CIP 0.01 mg/L
CIP 0.018 mg/L
CIP 0.5 mg/L
c
CTX 0.0125 mg/L
CTX 2 mg/L
d
0hrs
72hrs
Number of isolates
Number of resistances

Supplementary Figure 3. Selection for antibiotic-resistant *K. pneumoniae* in serially passaged (3x24 h) wastewater communities (n = 3) by two sterile-filtered wastewaters from the Sahlgrenska university hospital (HWW1 and HWW2; panel a) (90%) and by single antibiotics (ciprofloxacin and cefotaxime, b and c respectively) at breakpoint concentrations or at concentrations measured in the hospital wastewaters. Three complex municipal wastewater influent communities were exposed to either physiological saline (control), wastewaters or single antibiotics in saline and supplemented with 10% LB medium for 72 h (3 passages). The percentage of resistant *K. pneumoniae* was determined by plating a fraction of the culture on SCAi agar media with or without antibiotics. One-way ANOVA followed by Dunnett's multiple comparisons test was used to compare log-transformed resistance ratios at 72 h between hospital wastewater/antibiotics and physiological saline (control 0 h purple *, and control 72 h green *) (*p < 0.05; **p < 0.01, ***p < 0.001). Data are presented as mean values +/- SEM. All data and p-values associated with this figure are provided in the Source Data file. Abbreviations: AMC: amoxicillin-clavulanic acid; CAZ: ceftazidime; CDR: cefadroxil; CIP: ciprofloxacin; SXT: trimethoprim-sulfamethoxazole; TOB: tobramycin; TZP: piperacillin-tazobactam; PIP: piperacillin. Panel d: Increase in the proportion of multi-resistances among *K. pneumoniae* from serially a passaged sewage community exposed to 90% hospital wastewater and 10% LB medium. After cultivating aliquots on SCAi plates without any antibiotics, 26 isolates were randomly picked at 0 h and 26 after and 3x24 h serial passaging. Resistances to 8 antibiotics for each isolate were profiled using disc diffusion test as per EUCAST guideline v.13 (Supplementary Data 6).

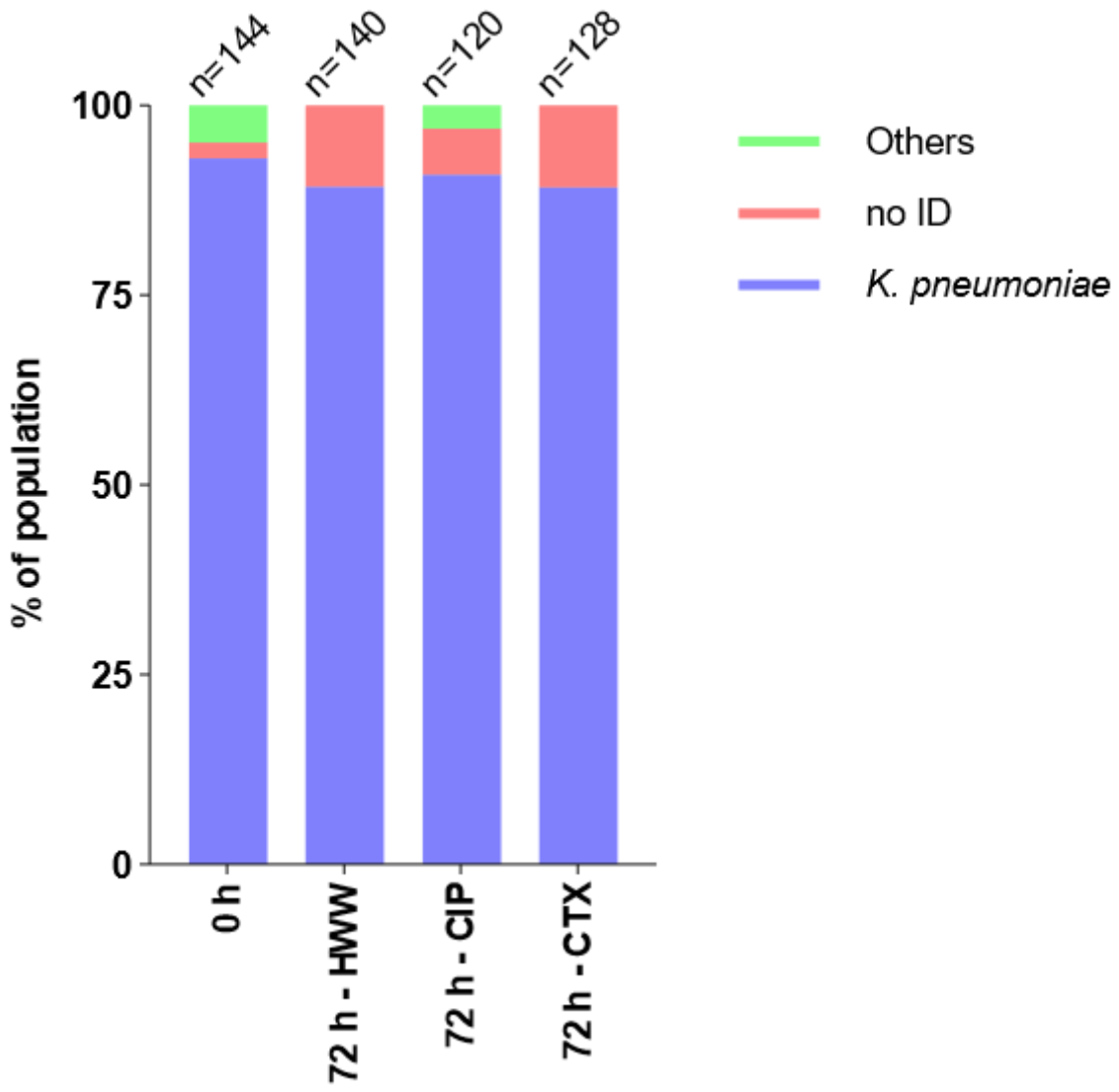

Supplementary Figure 4. Accuracy of colony-counting of presumed *K. pneumoniae* from complex, serially passaged sewage communities, and subsequently isolated on SCAi plates, as determined by MALDI-TOF MS at 0 h, at 72 h exposed to hospital wastewater, to 0.018 mg/L ciprofloxacin (72 h – CIP), or to 0.0125 mg/L cefotaxime (72 h – CTX). Note that the corresponding accuracy for isolating *E. coli* from complex sewage using CHROMagar™ ECC agar is typically above 99%[5]. Abbreviations: CIP: ciprofloxacin; CTX: cefotaxime; and HWW: hospital wastewater.

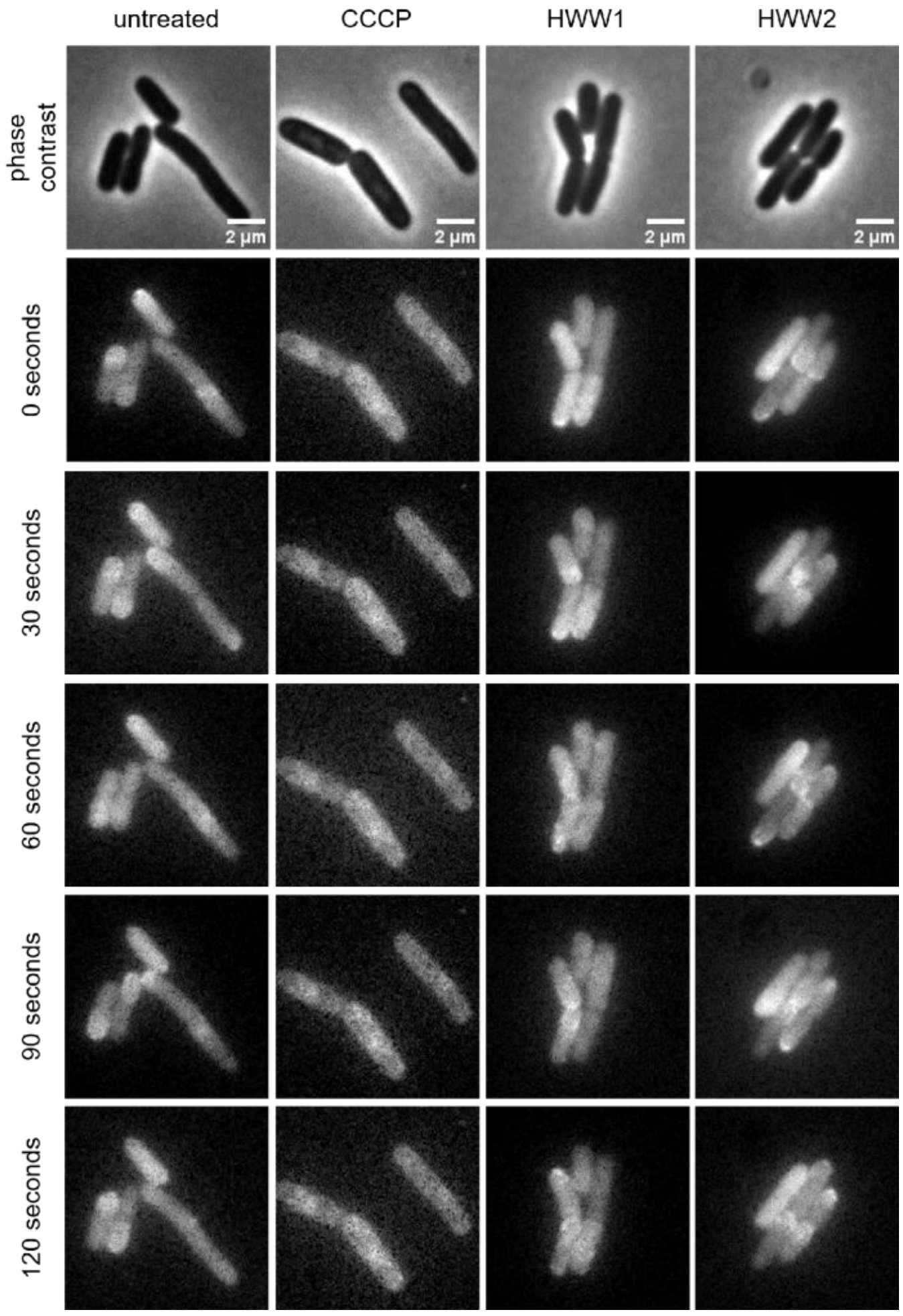

Supplementary Figure 5. Cellular effects of hospital wastewater (HWW) exposure are not a result of membrane potential depolarisation. For MinD oscillation, *E. coli* RC1 pFX9 was grown until an OD600 of 0.2 was reached, followed by addition of 10 µM isopropyl β- d-1-thiogalactopyranoside (IPTG) to induce the expression of GFP-MinD, (n = 3). After 30 min, antibiotics were added as for MreB experiments. For wastewater samples, 10 µM IPTG were re-added after resuspension in effluent. After 30 min of antibiotic treatment, timelapse microscopy was performed, recording images every 15 sec for 2 min. Images were analysed and stacks were converted into videos using ImageJ. Abbreviation: CCCP: carbonyl cyanide m-chlorophenyl hydrazone.

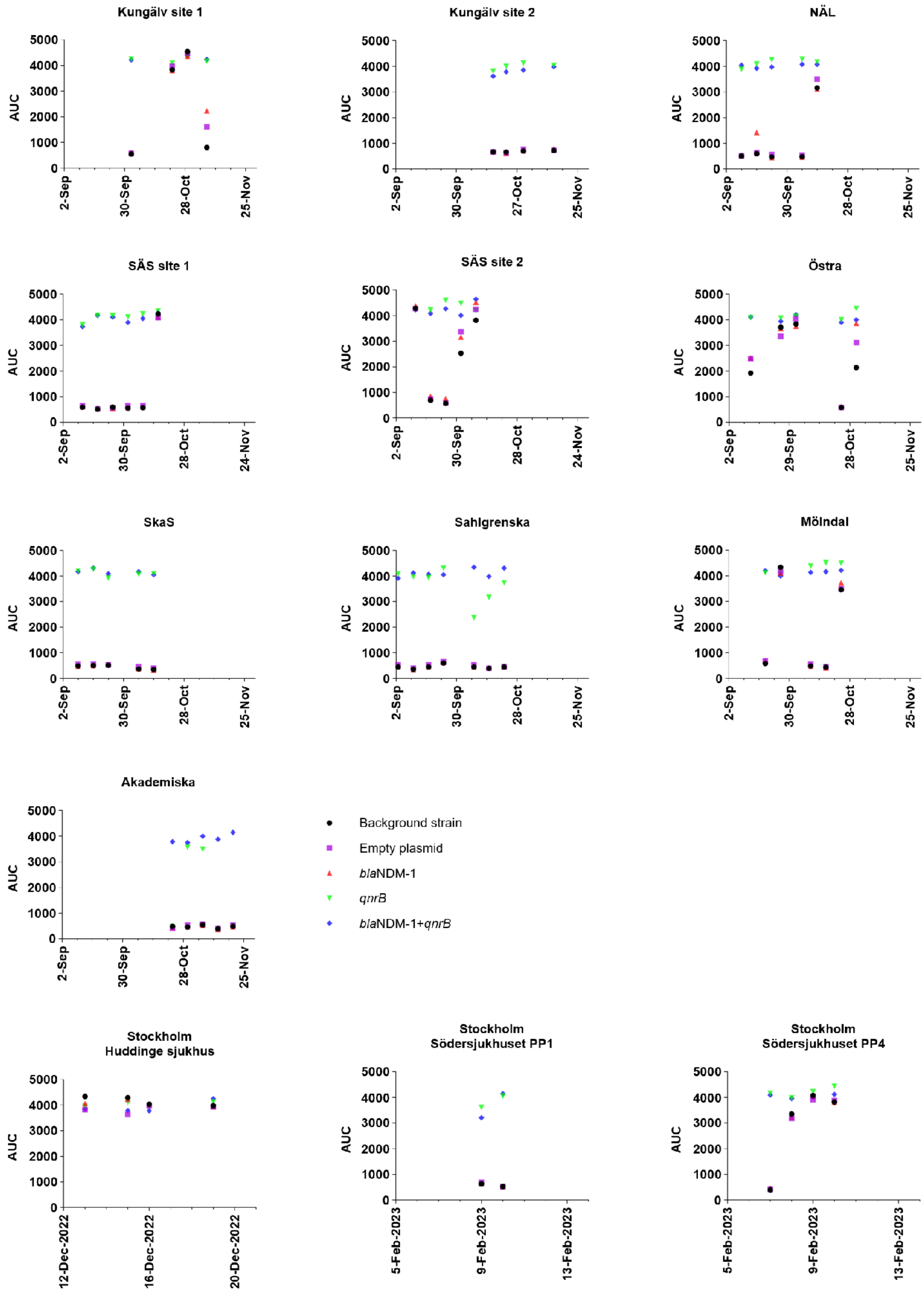

Supplementary Figure 6. Dependency of β-lactam and/or fluoroquinolone resistance for growth of *E. coli* in sterile-filtered wastewaters (24 h composite samples) from Swedish hospitals. Note that all samples were frozen without prior filtering, which leads to strong

reduction of several β-lactam antibiotics (Main Fig. 7 and Supplementary Data 8). Isogenic constructs were based on a non-resistant background strain (MC211), the background strain with an empty plasmid (pZE21) or with the same plasmid expressing either the *bla*NDM-1 gene, *qnrB* or both, as described in the methods section. Growth was assessed in the presence of 90% wastewater and 10% LB using the Omnilog™ system. Unless stated the sampling year was 2024. Source data are provided as a Source Data file. Abbreviations: SÄS: Södra Älvsborgs Sjukhus; NÄL: Norra Älvsborgs Sjukhus; SkaS: Skaraborgs Sjukhus; AUC: area under curve.

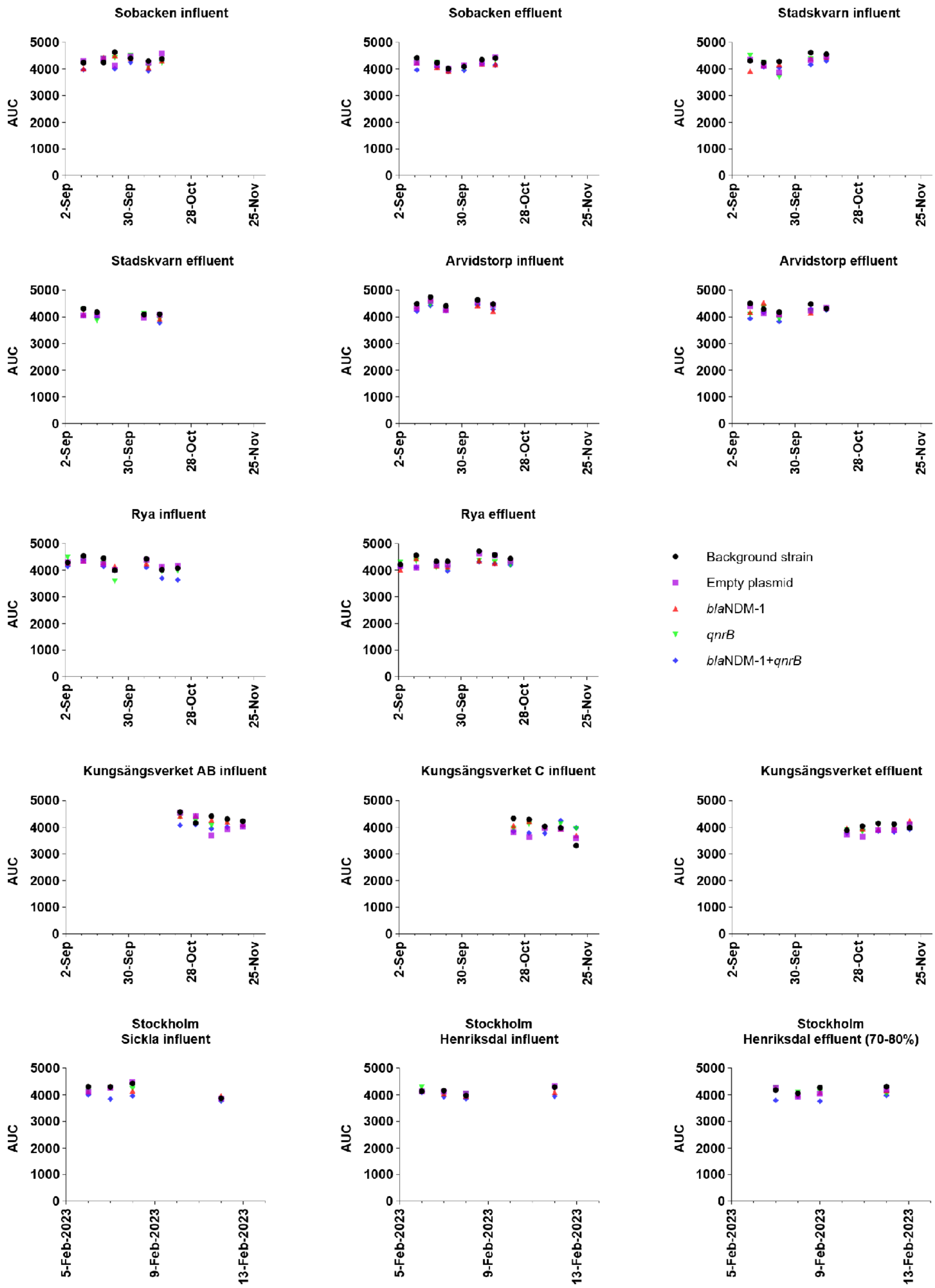

Supplementary Figure 7. Dependency of β-lactam and/or fluoroquinolone resistance for growth of *E. coli* in sterile-filtered wastewaters (24 h composite samples) from Swedish municipal treatment plants. Note that all samples were frozen without prior filtering. Isogenic constructs were based on a non-resistant background strain (MC211), the background strain

with an empty plasmid (pZE21) or with the same plasmid expressing either the *bla*NDM-1 gene, *qnrB* or both, as described in the methods section. Growth was assessed in the presence of 90% wastewater and 10% LB using the Omnilog^{TM} system. Unless stated the sampling year was 2024. Source data are provided as a Source Data file. Abbreviations: Rya; Ryaverket: AUC; area under curve.

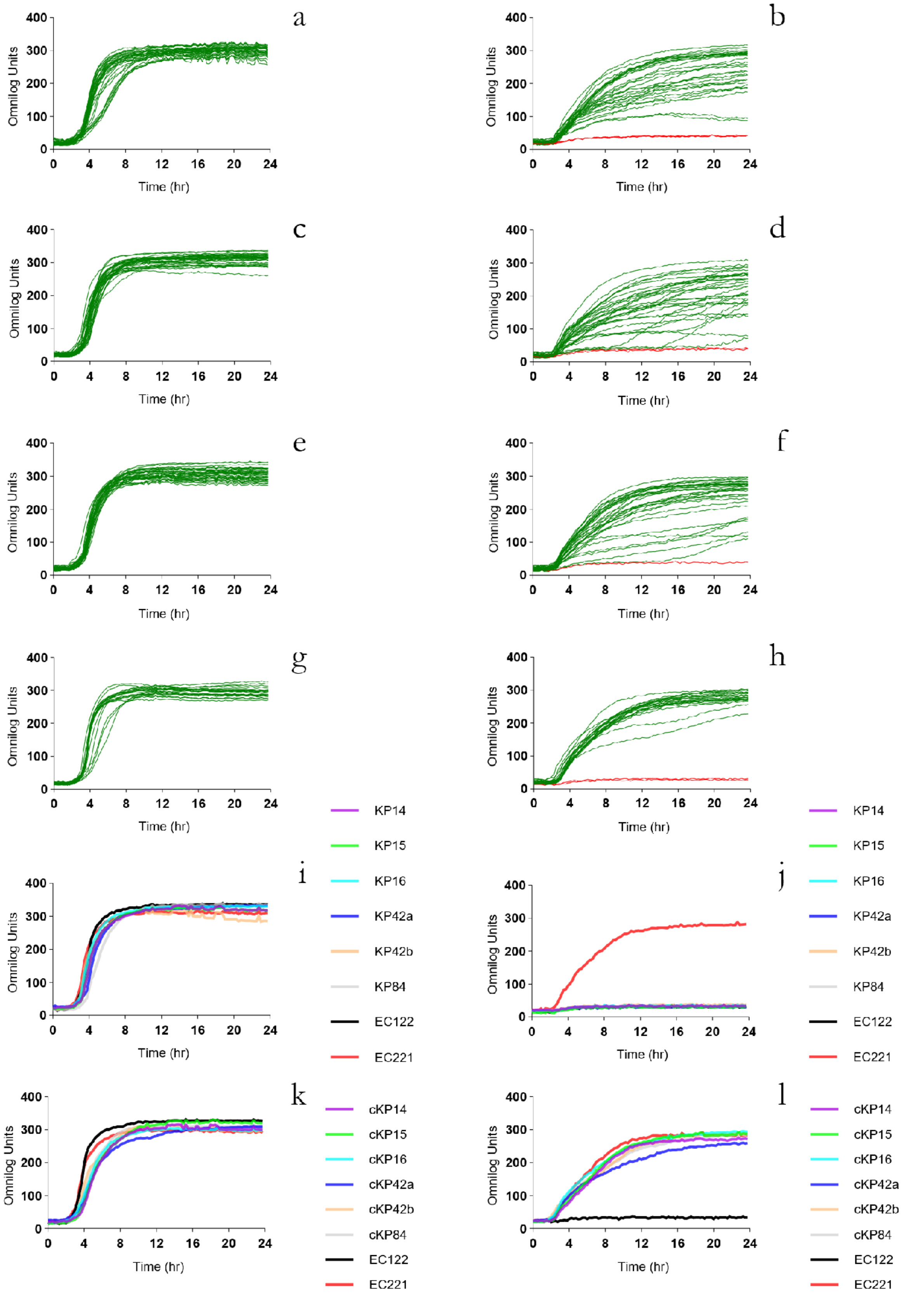

Supplementary Figure 8. Growth-inhibition of *K. pneumoniae* isolates by exposure to wastewater from the Sahlgrenska university hospital (HWW2) and rescue after adaptation to

ciprofloxacin as measured in the Omnilog™ system. 117 *K. pneumoniae* isolates were cultured in physiological saline (a, c, e and g) or HWW2 (b, d, f and h), both containing 10% LB media. Six of the isolates were then exposed to gradually increasing ciprofloxacin concentrations to reach an MIC of at least 0.01 mg/L. Panels (i) and (j) show the growth of the six original strains that did not grow in HWW2 in saline (i) and HWW2 (j), while panels (k) and (l) show growth of the corresponding ciprofloxacin-adapted strains in saline (k) and HWW2 (l), with 10% LB added to all wells. Two *E. coli* strains were included as controls.

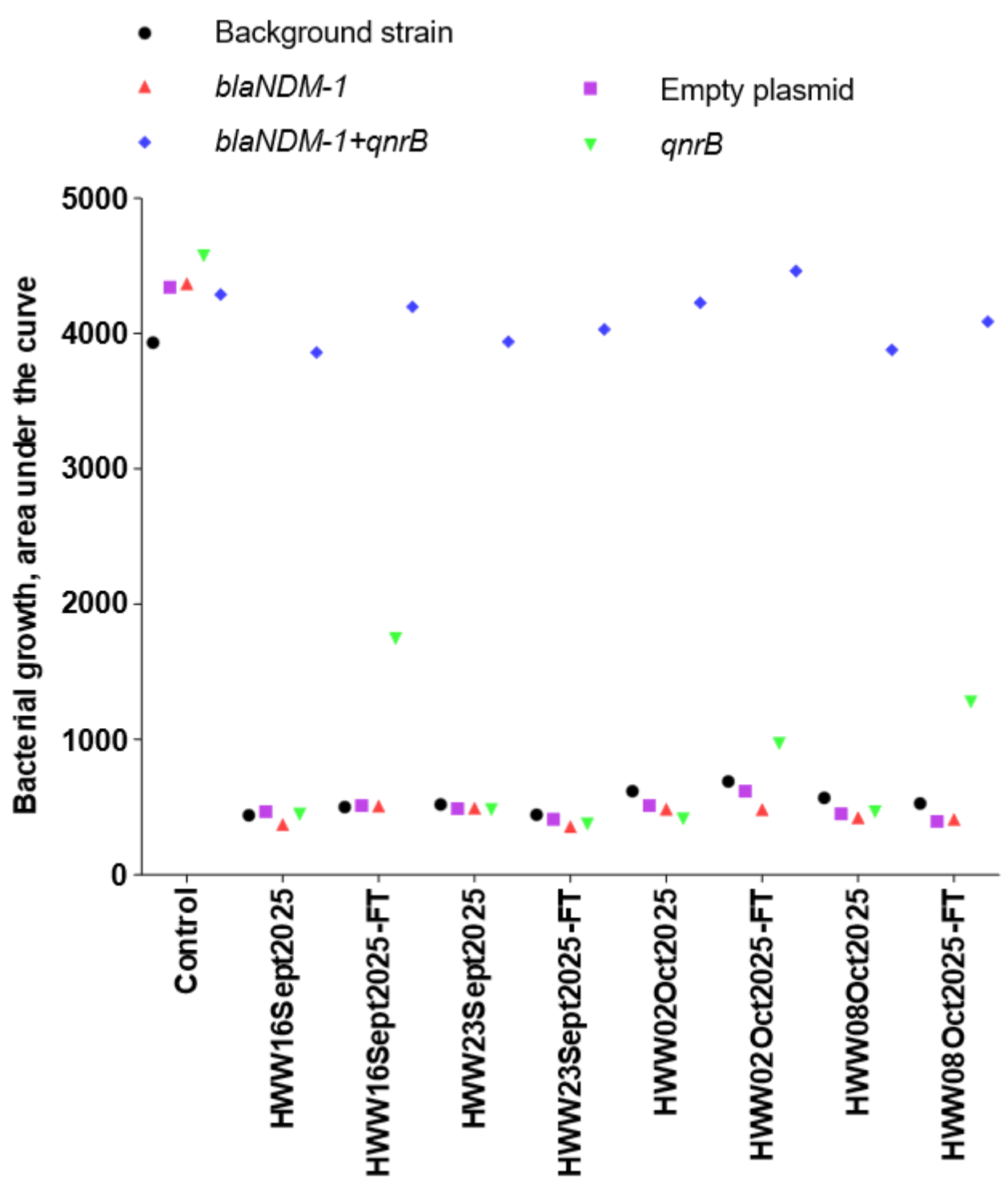

Supplementary Figure 9. Freezing and thawing unfiltered hospital wastewater reduces dependency on β-lactams for growth of *E. coli.* Wastewaters (24 h composite) were collected on four occasions in 2025 from the Sahlgrenska university hospital. For each sample, one aliquot was filtered immediately and frozen at -80 ºC (control). Another aliquot was frozen whole at -20 °C for 1-2 days, then thawed, filtered immediately, frozen and stored at -80 ºC until growth experiments with isogenic *E. coli* strains performed similarly as in supplementary figures 7 and 8. Source data are provided as a Source Data file. Abbreviations: HWW: hospital wastewater; FT: frozen and thawed before filtering.

Videos

Supplementary Video 1 pFX9_untreated 30 1

Supplementary Video 2 pFX9_CCCP 30 min 2

Supplementary Video 3 pFX9 HWW1 30 mir 3

Supplementary Video 4 FX9 HWW2 30 min 4

Supplementary Video 1-4. Cellular effects of HWW exposure are not a result of membrane potential depolarization. Full videos corresponding to snapshots shown in Supplementary Figure 5.

Methods

DNA Extraction

DNA extractions were performed by adhering the following protocol: 1-2 mL of a lysis buffer (50 mM EDTA, 50 mM Tris-HCl, 1 mM NaCl, 800 mM Guanidine HCl, 5% Triton X-100, 5% Tween 80, and 1 mg/ml lysozyme) were added to each sample and suspended so that very little particulate clumps are visible. Biofilm samples were vortexed for 5 minutes at top speed. Samples were incubated for 1 hour and 20 minutes at 55 °C while shaking at 800 rpm, followed by supplementation of Proteinase K (ThermoFisher Scientific, USA) to a final concentration of 100 µg/mL. Biofilm samples were vortexed for an additional 5 minutes at top speed. A second incubation was done at 37 °C for 1 hour and 20 minutes while shaking at 800 rpm.

Following incubation, an equal volume of Chloroform:Isoamyl alcohol 24:1 (Sigma-Aldrich, USA) was added and mixed in each sample. The mixture was centrifuged at 16,100 x g for 1 hour and 30 minutes at room temperature using an Eppendorf Centrifuge 5415R (Eppendorf, Germany). The top aqueous layer was carefully removed by pipetting, and 1/10 volume of 3 M sodium acetate and an equal volume of 2-propanol was added and mixed in each sample. The samples were incubated at -20 °C for 1 hour and 30 minutes followed by centrifuging for another 1 hour and 30 minutes at top speed at 4 °C. The resulting DNA pellet was airdried and dissolved in 100 µL of TE buffer.

RNase (ThermoFisher Scientific, USA) was added to a final concentration of 100 µg/mL and incubated at 37 °C for 30 minutes to remove RNA contamination from the DNA samples. The DNA was purified using a 1.8x ratio AmpureXP beads (Beckman Coulter, USA) and following the manufacturer's standard protocol. The DNA samples were eluted in 50 µL of nuclease-free water and stored at -20 °C until further analysis.

Metagenomic Sequencing

The Qubit 2.0 Fluorometer and Qubit dsDNA BR Assay Kit (ThermoFisher Scientific, USA) were used to assess the overall quality and quantity of the DNA prior to sequencing. All samples met the sequencing requirements (1.8 - 2 A260/A280 and >500 ng DNA per sample).

Sequencing was performed on a NovaSeqXPlus (NovaSeqXSeries Control Software 1.3.0.39308) with a 151nt(Read1)-10nt(Index1)-10nt(Index2)-151nt(Read2) setup using '25B' mode flow cell. The Bcl to FastQ conversion was performed using bcl2fastq_v2.20.0.422 from the CASAVA software suite. The quality scale used was Sanger / phred33 / Illumina 1.8+.

Metagenomic Data Analysis

An initial quality assessment was performed on the individual fastQ files using FastQC (v0.11.9) and summarized using MultiQC (v1.14). Although the overall quality of the reads was

high, a sequencing read trimming step was performed using BBMap (v39.01) and the bbduk.sh command was used to remove low quality flanking bases and residual adapter sequences. It was at this stage that all forward (R1) and reverse (R2) reads for each sample, originating from multiple sequencing lanes, were merged into single R1 and R2 files, respectively. A second quality assessment was performed to evaluate the outcome of the read trimming and all samples were approved for further analysis.

The Diamond BLAST software (v0.9.14) was used to match sequencing reads to a reference database to estimate the abundance of ARGs using ResFinder v2.0.2[6]. Diamond databases were prepared using the makedb command. Since Diamond requires amino acid input files, nucleotide reference sequences were translated into amino acid sequences using *transeq* (v6.6.0.0) after clustering identical sequences to remove duplicates using vsearch (v2.22.1). Diamond BLAST was performed on all samples and databases while maintaining a match inclusion criterion at a 70% id threshold and 80% query cover. A custom script was employed to extract read matches at a 90% identity threshold.

Microbial community composition was determined using the sylph (v0.8.1) metagenomic profiling software[7] and their proprietary GTDB_r226 reference database. The sylph command was run using the profile argument to generate a file containing the number of matches to individual bacterial genomes per sample. The sylph-tax command and the *taxprof* argument were used to transform the genome matches into relative abundance taxonomic classifications.

Supplementary References

1 Voigt, A. M. *et al.* The occurrence of antimicrobial substances in toilet, sink and shower drainpipes of clinical units: A neglected source of antibiotic residues. *Int J Hyg Environ Health* **222**, 455-467 (2019). https://doi.org/10.1016/j.ijheh.2018.12.013

2 Voigt, A. M. *et al.* Association between antibiotic residues, antibiotic resistant bacteria and antibiotic resistance genes in anthropogenic wastewater - An evaluation of clinical influences. *Chemosphere* **241**, 125032 (2020). https://doi.org/10.1016/j.chemosphere.2019.125032

3 Schuster, D. *et al.* Antibiotic concentrations in raw hospital wastewater surpass minimal selective and minimum inhibitory concentrations of resistant Acinetobacter baylyi strains. *Environ Microbiol* **24**, 5721-5733 (2022). https://doi.org/10.1111/1462-2920.16206

4 Yu, Z. *et al.* Antibiotic resistance selection and deselection in municipal wastewater from 47 countries. *Nat Commun* **16**, 9698 (2025). https://doi.org/10.1038/s41467-025-65670-7

5 Flach, C. F., Genheden, M., Fick, J. & Larsson, D. G. J. A Comprehensive Screening of Escherichia coli Isolates from Scandinavia's Largest Sewage Treatment Plant Indicates No Selection for Antibiotic Resistance. *Environ Sci Technol* **52**, 11419-11428 (2018). https://doi.org/10.1021/acs.est.8b03354

6 Florensa, A. F., Kaas, R. S., Clausen, P., Aytan-Aktug, D. & Aarestrup, F. M. ResFinder - an open online resource for identification of antimicrobial resistance genes in next-generation sequencing data and prediction of phenotypes from genotypes. *Microb Genom* **8** (2022). https://doi.org/10.1099/mgen.0.000748

7 Shaw, J. & Yu, Y. W. Rapid species-level metagenome profiling and containment estimation with sylph. *Nat Biotechnol* **43**, 1348-1359 (2025). https://doi.org/10.1038/s41587-024-02412-y
