# Supplementary material for: Widespread selection of antibiotic resistance within hospital sewers
Source: Nat Commun. 2026 Oct 29;17:11162. doi: 10.1038/s41467-026-78836-8 (PMC13720167; doi:10.1038/s41467-026-78836-8)
Supplement: Supplementary file 2 [file 41467_2026_78836_MOESM2_ESM.pdf]

Description of Additional Supplementary Files

Supplementary Data 1: Significance Two-sided PERMANOVA beta-diversity between groups.

Supplementary Data 2. Aggregated antibiotic usage data in wards at the Sahlgrenska university hospital contributing to the wastewater sampling point during the days just prior to sampling for hospital wastewaters collected in Jan 2022 (HWW1 and HWW2).

Supplementary Data 3. Measured concentration of antibiotics in hospital wastewater.

Supplementary Data 4. Antibiotic resistance profile of isogenic strains.

Supplementary Data 5. MICs of isogenic strains.

Supplementary Data 6. Resistance profiles of the randomly selected 52 K. pneumoniae isolates (26 from 0 h and 26 from 72 h) after selection experiment with HWW.

Supplementary Data 7. Resistance profile of the 117 K. pneumoniae strains as determined by disc diffusion.

Supplementary Data 8. Measured concentrations in hospital wastewater that was filtered immediately vs hospital wastewater having undergone freezing and thawing prior to filtering.

Supplementary Data 9. Measured antibiotic concentrations for hospitals and municipal treatment plant wastewaters.

Supplementary Data 10. Aggregated antibiotic usage data (grams) for the three days immediately prior to each sampling date.

Supplementary Data 11. Summary of antibiotic usage (proportion of total DDDs used) in VGR hospitals. Data are presented as median across sampling occations [1st-3rd quantile].

Supplementary Data 12. Summary antibiotic usage in VGR's hospitals. Data are presented as median [1st-3rd quantile] for the 3 days prior to each sampling date for each sampling location.

Supplementary Data 13. Validation of the chemical analytical protocol of Meropenem.

Supplementary Data 14. The approximate patient beds that supply sampling hospital sites.

Supplementary Data 15. Mass spectrophotometry parameters.

Supplementary Data 16. List of primers used to construct plasmids.

Supplementary Data 17. Summary of the samples and their relevant metadata used in this study.

Supplementary Video 1-4. Cellular effects of HWW exposure are not a result of membrane potential depolarization. Full videos corresponding to snapshots shown in Supplementary Figure 5.
