# Supplementary material for: Widespread selection of antibiotic resistance within hospital sewers
Source: Nat Commun. 2026 Oct 29;17:11162. doi: 10.1038/s41467-026-78836-8 (PMC13720167; doi:10.1038/s41467-026-78836-8)
Supplement: Supplementary file 3 [file 41467_2026_78836_MOESM8_ESM.pdf]

nature portfolio

Corresponding author(s): D.G.Joakim Larsson

Last updated by author(s): Jul 22, 2026

Reporting Summary

Nature Portfolio wishes to improve the reproducibility of the work that we publish. This form provides structure for consistency and transparency in reporting. For further information on Nature Portfolio policies, see our Editorial Policies and the Editorial Policy Checklist.

Statistics

For all statistical analyses, confirm that the following items are present in the figure legend, table legend, main text, or Methods section.

n/a	Confirmed	
☐	☒	The exact sample size (n) for each experimental group/condition, given as a discrete number and unit of measurement
☐	☒	A statement on whether measurements were taken from distinct samples or whether the same sample was measured repeatedly
☐	☒	The statistical test(s) used AND whether they are one- or two-sided<br>*Only common tests should be described solely by name; describe more complex techniques in the Methods section.*
☐	☒	A description of all covariates tested
☐	☒	A description of any assumptions or corrections, such as tests of normality and adjustment for multiple comparisons
☐	☒	A full description of the statistical parameters including central tendency (e.g. means) or other basic estimates (e.g. regression coefficient) AND variation (e.g. standard deviation) or associated estimates of uncertainty (e.g. confidence intervals)
☐	☒	For null hypothesis testing, the test statistic (e.g. F, t, r) with confidence intervals, effect sizes, degrees of freedom and P value noted<br>*Give P values as exact values whenever suitable.*
☒	☐	For Bayesian analysis, information on the choice of priors and Markov chain Monte Carlo settings
☒	☐	For hierarchical and complex designs, identification of the appropriate level for tests and full reporting of outcomes
☒	☐	Estimates of effect sizes (e.g. Cohen's d, Pearson's r), indicating how they were calculated

Our web collection on statistics for biologists contains articles on many of the points above.

Software and code

Policy information about availability of computer code

Data collection

No software was used to collect data.

Data analysis

GraphPad Prism (v10.1.0(316)) was used to perform one way anovas in the statistical analysis of selection toward antibiotic resistances.

The R code for the core statistical analysis of resistance rates in E.coli and ARG carriage rates is s uploaded and publicly available in a GitHub repository at https://github.com/watNoel/VGRegion-Hospital-Wastewater-Selection-Project .
For running this code, R version 4.4.0 is required along with the packages tidyverse version 2.0.0, glmmTMB version 1.1.14 , emmeans version 2.0.1 and DHARMa version 0.4.7

For the processing of metagenomics data generated for this study, detailed descriptions of software and versions are available in the Methods and Supplementary methods of the manuscript. Scripts for this processing are also available in aforementioned GitHub repository.
Briefly, for the processing of metagenomic data,

Python 3.9.15 was used, with packages (version) pandas 1.5.2, numpy 1.23.5 , tqdm 4.67.1 and the external tools (with version & purpose)
FastQC 0.11.9 Quality control of raw and trimmed sequencing reads
MultiQC 1.14 Aggregation of QC reports across samples
BBMap (bbduk) 39.01 Adapter removal and quality trimming
VSEARCH 2.22.1 Sequence clustering and duplicate removal
EMBOSS (transeq) 6.6.0 Translation of nucleotide sequences to protein sequences
DIAMOND 0.9.14 Fast alignment of reads against protein reference databases
Sylph 0.8.1 Metagenomic taxonomic profiling

April 2023

Sylph-tax 1.5.1 Taxonomic annotation and abundance summarization
vegan 2.7.1 in R (4.5.1) Bray–Curtis distance and diversity metrics

Finally,
The map of the sampling sites was made in R using R R (v 4.4.0) with packages sf (v, 1.1.0), dplyr (v. 1.1.4), ggplot2 (v. 4.0.0) and ggrepel (v. 0.9.6).

For manuscripts utilizing custom algorithms or software that are central to the research but not yet described in published literature, software must be made available to editors and reviewers. We strongly encourage code deposition in a community repository (e.g. GitHub). See the Nature Portfolio guidelines for submitting code & software for further information.

Data

Policy information about availability of data

All manuscripts must include a data availability statement. This statement should provide the following information, where applicable:

- Accession codes, unique identifiers, or web links for publicly available datasets
- A description of any restrictions on data availability
- For clinical datasets or third party data, please ensure that the statement adheres to our policy

Data generated for this study are provided in the main article, supplementary dataset and source data. Metagenomic sequencing data have been uploaded to NCBI with accession codes: SAMN55849194–SAMN55849290 under BioProject PRJNA1426335.

Research involving human participants, their data, or biological material

Policy information about studies with human participants or human data. See also policy information about sex, gender (identity/presentation), and sexual orientation and race, ethnicity and racism.

Reporting on sex and gender	Not applicable.
Reporting on race, ethnicity, or other socially relevant groupings	Not applicable.
Population characteristics	Not applicable.
Recruitment	Not applicable.
Ethics oversight	Not applicable.

Note that full information on the approval of the study protocol must also be provided in the manuscript.

Field-specific reporting

Please select the one below that is the best fit for your research. If you are not sure, read the appropriate sections before making your selection.

☐ Life sciences ☐ Behavioural & social sciences ☒ Ecological, evolutionary & environmental sciences

For a reference copy of the document with all sections, see nature.com/documents/nr-reporting-summary-flat.pdf

Ecological, evolutionary & environmental sciences study design

All studies must disclose on these points even when the disclosure is negative.

Study description	The study involved collection of wastewater and biofilm samples from multiple hospitals, municipal wastewater treatment plant influents across west Sweden and Stockholm/Uppsala in Sweden. From these samples, bacterial isolates, metagenomics data and filtered wastewaters were retrieved and used in analysis. For the statistical comparisons of resistance rates in E.coli and enrichment of ARGs in the metagenomes across biofilms/waters at municipal influent and hospital sites, hierarchical models with random effects for the different sites, including an interaction term between matrix(biofilm or water) and site type (hospital or municipal influent) were fitted. These models took one observation per sampling point and time (one per biological replicate which ranged from 1 for the biofilms to 4-7 for the wastewater samples). For the E.coli counts data, the input for each sample (biological replicate) consisted of the median of technical replicates which were four per sample, except for the hospital biofilm samples which were tested with 16 technical replicates per biofilm. The study also involved comparisons of bacterial characteristics, concentrations of antibiotics and selective potential of wastewaters from hospitals and municipal wastewater treatment plants. futher details on the level of replication and statistical comparisons made for these assessments are explained in the main manuscript file.
Research sample	Samples came from the six largest hospitals in western Sweden and their corresponding municipal treatment sites. In addition, we

Research sample	received samples from Uppsala (main hospital and corresponding municipal treatment plant) and Stockholm (two hospitals with one common treatment plant).<br>These samples are meant to represent the conditions in hospital/municipal wastewater treatment plants in Sweden with a focus on antibiotic residues, wherefore multiple hospitals were samples, at multiple occasions, in multiple regions of the country.
Sampling strategy	No sample size calculations were performed a priori. Our findings of significant effects indicates that samples sizes were sufficient.
Data collection	CFU data was recorded manually, in most cases by two people (Declan and Mutshiene), on pen and paper. Bacterial growth data was recorded using a robot (Omnilog). Sequencing data was produced with a NovaSeqXPlus system by NGI Sweden.Microscopy data was generated using Nikon Eclipse Ti2 by Michaela and Declan. Chemical analyses data was recorded by the mass spectrometer instrument software as described in the manuscript, by Jerker.
Timing and spatial scale	Sampling started in 2022 (Gothenburg) and stopped in late 2025 as outlined in detail in the manuscript. A large regional sampling was conducted during September until November 2024 across western Sweden and Uppsala in which samples were collected at a minimum of 4 days apart. Additional samples from 2022-2023 from Stockholm were included as described. Analyses of samples took place throughout this period.
Data exclusions	No data were excluded from any analyses. We discovered during the course of the study that the sampling protocol applied for the regional sampling in 2024 led to rapid degradation of several betalactams (but not other antibiotics) as demonstrated by separate, dedicated experiments (data shown in the main manuscript). Analytical data for betalactams is therefore not reported for the 2024 sampling campaign, since we believe it is misleading.
Reproducibility	All replications of experiments are included (no exclusion of any failed attempts to replicate results). During the finalization of this study, we identified in a parallel ongoing study that hospital biofilms tended to harbor a high proportion of Citrobacter freundii, which can appear morphologically similar to Escherichia coli on ECC agar. We therefore reanalysed all CFU-based analyses of hospital biofilms included in the present study and used MALDI-TOF identification to ensure that only confirmed E. coli colonies were counted. The reanalysed data were retained and are presented in the study (Main Figure 3), whereas the original dataset, which may have been affected by C. freundii contamination, was discarded.<br>Importantly, both datasets indicated significant enrichment of resistant bacteria from the corresponding hospital wastewaters, although the magnitude of enrichment differed between the datasets.<br>We also analysed selected samples from other source types included in the present study, including municipal biofilms, hospital wastewater, and municipal wastewater, and found no evidence of relevant C. freundii contamination in these samples.
Randomization	Not relevant. It is obvious which sites are hospitals and which ones are municipal wastewater treatment plants. During lab-based exposure experiments, identical inocula were used across treatment groups.
Blinding	Blinding was not applied. The type of data generated is not inherently sensitive to observer bias, such as e.g. subjective scoring of histological effects.

Did the study involve field work? ☒ Yes ☐ No

Field work, collection and transport

Field conditions	Dates are included for all samples. Wastewaters are only marginally affected by seasonal fluctuations in temperature as it is transported far underground. Cooled samplers were always used, samples were transprted cool to the laboratory before proccessed alternatively frozen directly as described in the paper. Hospitals and muncipal sites, which constitutes an improtant comparison, were sampled in parallel.
Location	Coordinates of sampling locations:<br>Name Lon Lat<br>Sahlgrenska hospital 11.9549 57.6833<br>Mölndal 12.0129 57.6614<br>Östra 12.0522 57.7226<br>NÄL 12.2658 58.3187<br>SkaS 13.8571 58.4525<br>Kungälv 11.9694 57.8784<br>SÄS 12.9615 57.7244<br>Ryaverket 11.8931 57.6976<br>Sobacken 12.9157 57.6523<br>Arvidstorp 12.2583 58.2751<br>Stadskvarn 13.8776 58.3899<br>Akademiska sjukhuset 17.6403 59.8499<br>Södersjukhuset 18.0559 59.3099<br>Huddinge 17.9344 59.2198<br>Kungsängsverket 17.6599 59.8441<br>Henriksdal 18.1082 59.3113
Access & import/export	All samples were collected in close collaboration with, and with the explicit support and assistance of, the regional hospitals and healthcare organizations, as well as the municipalities responsible for operating the municipal wastewater treatment plants. There were no imports/exports ass all samples were collected and processed in Sweden

Disturbance	Not Applicable

Reporting for specific materials, systems and methods

We require information from authors about some types of materials, experimental systems and methods used in many studies. Here, indicate whether each material, system or method listed is relevant to your study. If you are not sure if a list item applies to your research, read the appropriate section before selecting a response.

Materials & experimental systems

n/a	Involved in the study
☒	☐ Antibodies
☒	☐ Eukaryotic cell lines
☒	☐ Palaeontology and archaeology
☒	☐ Animals and other organisms
☒	☐ Clinical data
☒	☐ Dual use research of concern
☒	☐ Plants

Methods

n/a	Involved in the study
☒	☐ ChIP-seq
☒	☐ Flow cytometry
☒	☐ MRI-based neuroimaging

Plants

Seed stocks	Not applicable.
Novel plant genotypes	Not applicable.
Authentication	Not applicable.
