# Supplementary material for: Widespread selection of antibiotic resistance within hospital sewers
Source: Nat Commun. 2026 Oct 29;17:11162. doi: 10.1038/s41467-026-78836-8 (PMC13720167; doi:10.1038/s41467-026-78836-8)
Supplement: Supplementary file 4 [file 41467_2026_78836_MOESM9_ESM.pdf]

nature portfolio

Peer Review File

Widespread selection of antibiotic resistance within hospital sewers

Corresponding Author: Professor D.G. Joakim Larsson

This file contains all reviewer reports in order by version, followed by all author rebuttals in order by version.

Attachments originally included by the reviewers as part of their assessment can be found at the end of this file.

Version 0:

Reviewer comments:

Reviewer #1

(Remarks to the Author)
This well written article provides novel information about the importance of hospital wastewater as a driver of antibiotic resistance selection among E. coli in Sweden. An abundance of results are shared, yet the interpretation of the importance of the many results presented is lacking. Further, the limitations and potential generalizability (or not) of the results needs to be further explored.

Broad Comments
- Add limitations in the discussion section.
- Methods were not consistent between all sites/samples. This is acknowledged throughout the manuscript but presents difficulties in results interpretation.
- The discussion section does not place these results in the context of other related published literature – these comparisons need to be added.

Specific comments

Introduction
- Lines 55-57: “The corresponding number for the general population in Sweden is about one in 300 people, explaining the much higher levels of antibiotics typically found in hospital wastewater (HWW) compared to municipal wastewater.”
o Information is needed here on the levels of antibiotics previously reported in municipal wastewater.
- Lines 77-79: “A major aim of this study was therefore to move beyond laboratory studies and comparisons of concentrations, limited to show potential for selection, by also assessing evidence for on-site selection within the sewers” – however as described in the introduction the study is still largely lab-based. There are elements of the study that are applied, including the comparison of wastewater with biofilms – this needs to be emphasized as the field testing/applied aspects of the research.

Results
- Add a map to give context for international readers about the location of the Västra Götaland Region, Uppsala, and Stockholm and the sampling locations within this region and cities.
- Lines 108-109: “The significant selection was apparent regardless of which controls were applied (i.e saline at 0 h or 72 h).” – from Figure 1 it appears that this differed by sampling site, at some hospitals (ex. SAS site 2) significant selection was only apparent when compared to the 72 h control. This statement needs to be revised to include more nuance.
- Add analysis to determine if resistance selection results differ significantly between sampling sites
- Report p-values <0.001 as “<0.001” throughout results section
- Round all p-values >0.001 to 2 decimal places
- Lines 169-170: explain why these 8 antibiotics were selected.

Discussion
- Line 386 – add here data on usage of ciprofloxacin in countries other than Sweden and globally.
- Lines 395-397 – both sentences require citations for the statements made.

- Lines 405-413: Regarding suggestions for alternative antibiotics – add information here about clinical implications (ex. side effects) of alternatives suggested.
- Lines 411-413 – "This shows that some level of replacement to reduce selection risks in wastewaters is likely feasible, aiming for mutually beneficial outcomes without compromising patient safety." – add information here about what the impact is on patient safety from the suggested alternative antibiotics.
- Need to add discussion of significance of higher resistance rates in biofilms vs wastewater
- Need to add limitations
- Need more information about antibiotic usage in Sweden and how it compares to other countries – are these results generalizable?
- Need information about how these results might or might not apply to other bacteria.

Methods
- Samples were collected during different seasons from different hospitals. This could have impacted results as infections (and hence shedding) have seasonal variability. This needs to be acknowledged in a limitations section in the discussion section.
- Section 4.2 – why antibiotic usage data only for Region Västra Götaland?
- Lines 550-551 – add what antibiotics the blaNDM-1 and qnrB confer resistance against.
- Lines 616-617 – why these concentrations?
- Lines 632-633 - add target concentrations for DNA.

(Remarks on code availability)
NA

Reviewer #2

(Remarks to the Author)
Dear authors - please see comments attached as a .pdf document.

(Remarks on code availability)

Reviewer #3

(Remarks to the Author)
I congratulate the authors on a strong and ambitious study. The manuscript addresses an important question in environmental antimicrobial resistance: do hospital sewers only carry resistant bacteria from patients, or do they actively select for resistance? The authors design the entire study around this gap, which is conceptually important and well-executed.

Here is my report adapted to the guidelines for referees:

KEY RESULTS

The authors investigate whether hospital sewers actively select for antibiotic-resistant bacteria or only carry resistant bacteria shed by patients. They sample wastewater and sewer biofilms from 10 Swedish hospitals and matched municipal sites, and combine community selection assays, in situ biofilm colonization on plastic plates, shotgun metagenomics, LC-MS/MS antibiotic measurements, ward-level usage data, fluorescence microscopy of MreB and MinD, and engineered isogenic E. coli strains. The central finding is that hospital wastewaters consistently select for ciprofloxacin- and cefotaxime-resistant E. coli, while municipal wastewaters do not. Resistant E. coli are also enriched in hospital sewer biofilms compared with paired wastewater, but not at municipal sites. The isogenic rescue with qnrB and blaNDM-1 identifies fluoroquinolones and β-lactams as the dominant drivers, and ciprofloxacin and cefotaxime as the principal selective agents.

SIGNIFICANCE

Earlier studies have shown higher resistance levels in hospital wastewater than in municipal wastewater, but this alone cannot separate selection from simple shedding by patients. The authors designed the study explicitly around this gap. The biofilm experiment is novel and provides (to my knowledge) some of the strongest field-level evidence to date for selection driven by excreted human pharmaceuticals in a natural environment. The identification of actionable drivers also makes the work directly relevant for hospital wastewater management and antimicrobial stewardship. As a side contribution, the freeze-thaw artifact affecting β-lactam recovery is itself useful for wastewater monitoring. The work compares well with the authors' prior single-hospital study (Kraupner et al. 2021) and clearly extends it.

VALIDITY AND SUPPORT FOR CONCLUSIONS

The central conclusion, that Swedish hospital wastewaters select for resistant E. coli, mainly through fluoroquinolones and β-lactams, is well supported by multiple independent lines of evidence that converge despite different limitations. However, several specific claims need more careful framing or additional evidence, as listed below.

MAJOR POINTS

1. Soften the "in-situ selection" claim. The biofilm pattern does not exclude alternative explanations, such as differential colonization, persistence, repeated seeding from upstream resistant bacteria, or site-specific physical conditions. Please change phrases such as "direct field evidence" and "conclusively linked" to "evidence consistent with" or "strong evidence supporting" in-situ selection. The abstract sentence "Here we show selection of antibiotic-resistant bacteria within Swedish hospital sewers" is too strong as written.

2. Biofilm sampling has no biological replication within sites. Each site contributed a single biofilm with technical replication only. Please acknowledge this limitation explicitly in the Discussion and state it in the Results.

3. Separate strong local β-lactam evidence from broader claims. Because the 2024 samples were frozen before filtration, β-lactam concentrations from this collection are unreliable. Ciprofloxacin selection is supported across all hospitals; β-lactam selection is strongly supported at Sahlgrenska and by phenotypic assays, but direct concentration-based attribution of cefotaxime across all hospitals is weaker. Please make this distinction clearly. The abstract should also briefly mention the limitations of sample handling.

4. Clarify the role of blaNDM-1. blaNDM-1 is a broad-spectrum carbapenemase and is unlikely to represent the dominant β-lactam resistance mechanism in Swedish hospital sewer E. coli, where ESBLs are likely more common. Please state clearly that blaNDM-1 is used as a functional rescue tool, not as a claim that NDM-like genes are the naturally selected determinants.

5. Strengthen or moderate the metagenomics. Total mobile ARG counts per million reads are confounded by taxonomic differences (PERMANOVA $R^2 = 0.37$). If feasible, add ARG class-specific analyses, link ARGs to taxa or mobile elements, or use models accounting for taxonomy. If not feasible, please present the metagenomics as sa upportive context. The borderline result ($p \approx 0.047$) should be described as a "trend," not "enrichment."

6. Moderate the extrapolation beyond Sweden. Sweden has unusually low community antibiotic use. Prescribing patterns, sewer infrastructure, temperature, and baseline AMR vary across countries, especially in low- and middle-income country settings.

7. Bound the taxonomic scope. Both E. coli and K. pneumoniae are members of the Enterobacterales. The conclusion that quinolones and β-lactams are the dominant drivers may not apply to anaerobes, non-fermenters, or Gram-positives. Please address this point in the Discussion.

8. Discuss non-antibiotic co-selection. Hospital wastewater contains disinfectants, metals, biocides, and other drugs. The isogenic experiments do not exclude co-selective effects on biofilm community structure or on non-Enterobacterales taxa.

9. Restrain claims about horizontal gene transfer and pathogen emergence. The study shows selection of resistant phenotypes, not mobilization or transfer. Please separate demonstrated results from inferential downstream risks.

DATA AND METHODS

The methodology is sound and thorough. Some specific issues require attention:

Sequence data availability must be rectified. I tried to access BioProject PRJNA1426335 and BioSamples SAMN55849194–SAMN55849290 on NCB/ENAI but was unable to locate them. This may be a typo in the accession numbers, or the data may not yet be public. This must be resolved before the manuscript can proceed. Please verify the accessions, ensure the data are released or accessible to reviewers via a private link, and confirm completeness.

Reproducibility infrastructure. The GitHub repository is well-intentioned, but some improvements are needed. The encryption of input data with a reviewer-only passkey is a barrier — please replace it with a private GitHub link, a Zenodo reviewer token, or a Figshare reviewer link. The visualization R scripts list dependencies as x.x.x rather than real version numbers; please pin versions, especially vegan and pairwiseAdonis.

MINOR POINTS

State the primary hypotheses explicitly at the end of the Introduction; clarify what is new compared to Kraupner et al. 2021.

Revise the abstract's "complete rescue" wording — qnrB alone rescued growth in most but not all samples; rescue is complete only with both genes.

Clarify whether municipal samples were temporally and geographically matched to hospital samples and whether storage durations differed.

Clarify how biofilm resistance rates were normalized (surface area, biomass, total E. coli, DNA yield).

Report absolute E. coli counts in biofilms and wastewater, and indicate detection-limit issues.

Describe the 90% wastewater + 10% LB / 37 °C assay as a selection-potential assay, not a reconstruction of sewer ecology.

For the isogenic strains, clarify possible plasmid backbone effects, expression levels, and fitness cost in non-selective wastewater.

Note that ResFinder annotation may miss chromosomal mutations (e.g., gyrA / parC) and divergent environmental homologs.

Acknowledge that serial-passage K. pneumoniae mutants may carry undefined additional adaptations.

Provide a table mapping each figure to the script that produces it; complete the empty "Citation" section in the README.

CLARITY, CONTEXT, AND REFERENCES

The manuscript is well-written and well-organized. The Introduction is clear and convincingly distinguishes selection from shedding. References are appropriate.

EXPERTISE

My review covers microbiology, genomics, bioinformatics, and infectious disease surveillance. I cannot fully assess the fluorescence microscopy quantification or the LC-MS/MS analytical chemistry.

SUMMARY

This is a strong, ambitious, and policy-relevant manuscript. The core conclusions are well supported. The revisions needed include reframing causal claims, clarifying the evidence for β-lactam antibiotics, acknowledging limits on biofilm replication, presenting metagenomics more conservatively, moderating extrapolation, and improving statistical and computational reproducibility. The missing BioProject data must be resolved.

(Remarks on code availability)

Open Access This Peer Review File is licensed under a Creative Commons Attribution 4.0 International License, which permits use, sharing, adaptation, distribution and reproduction in any medium or format, as long as you give appropriate credit to the original author(s) and the source, provide a link to the Creative Commons license, and indicate if changes were made.
In cases where reviewers are anonymous, credit should be given to 'Anonymous Referee' and the source.
The images or other third party material in this Peer Review File are included in the article's Creative Commons license, unless indicated otherwise in a credit line to the material. If material is not included in the article's Creative Commons license and your intended use is not permitted by statutory regulation or exceeds the permitted use, you will need to obtain permission directly from the copyright holder.
To view a copy of this license, visit https://creativecommons.org/licenses/by/4.0/

Reviewer #1 (Remarks to the Author):

This well written article provides novel information about the importance of hospital wastewater as a driver of antibiotic resistance selection among E. coli in Sweden. An abundance of results are shared, yet the interpretation of the importance of the many results presented is lacking. Further, the limitations and potential generalizability (or not) of the results needs to be further explored.

Broad Comments
- Add limitations in the discussion section.
- Methods were not consistent between all sites/samples. This is acknowledged throughout the manuscript but presents difficulties in results interpretation.
- The discussion section does not place these results in the context of other related published literature – these comparisons need to be added.

We are pleased that the reviewer recognizes the novelty of the study, while also identifying areas where additional clarification is needed. The broader points raised regarding the importance of the results, limitations discussed in the manuscript, generalizability of the findings, and the contextualization of our results with the existing literature are addressed in detail under the specific comments below.

We also acknowledge the reviewer's concerns regarding methodological inconsistencies across the study, particularly with respect to sample handling and processing. As noted in the manuscript, this work represents a large-scale study conducted over several years, during which methodological refinements were implemented as new insights emerged. We have aimed to be fully transparent about these changes, including how and why they arose, and we have clarified where methodological differences may have had implications for interpretation. We agree that an abundance of results are included in the manuscript, however, we believe we have highlighted the major findings in an appropriate manner in the main and extended figures. We have modified, added and combined certain figures to make our message clearer.

Specific comments

Introduction
- Lines 55-57: "The corresponding number for the general population in Sweden is about one in 300 people, explaining the much higher levels of antibiotics typically found in hospital wastewater (HWW) compared to municipal wastewater."

o Information is needed here on the levels of antibiotics previously reported in municipal wastewater.

In addition to previous references included, we have added a reference to an additional review article supporting our statement (Rodriguez-Serin et al, no 22). Table 2 and Table 3 in the cited review reports concentration ranges in untreated wastewater from municipal wastewater treatment plants versus untreated wastewater from hospitals. As pointed out by the reviewer, we recognize the value of also providing the readers with an overall ballpark figure of concentrations, at the same time recognizing that it is difficult to make a concise, general and still correct statement on the levels found, as concentrations vary between individual antibiotics as well as between different regions with different usage patterns. In addition to the added reference, we have now added that in municipal wastewaters, one typically finds up to low micrograms per litre for certain antibiotics, whereas in hospital wastewaters, reports of hundreds of micrograms per litre are not uncommon. We think this captures the overall picture reasonably well.

- Lines 77-79: "A major aim of this study was therefore to move beyond laboratory studies and comparisons of concentrations, limited to show potential for selection, by also assessing evidence for on-site selection within the sewers" – however as described in the introduction the study is still largely lab-based. There are elements of the study that are applied, including the comparison of wastewater with biofilms – this needs to be emphasized as the field testing/applied aspects of the research.

We thank the reviewer for the comment, and we fully agree. We have re-written this section to more accurately reflect the work presented in the manuscript, clarifying that it includes both experimental and *in situ* investigations.

Results

- Add a map to give context for international readers about the location of the Västra Götaland Region, Uppsala, and Stockholm and the sampling locations within this region and cities.

We thank the reviewer for this good suggestion. A map has been added to the manuscript. Please see new Figure 1.

- Lines 108-109: "The significant selection was apparent regardless of which controls were applied (i.e saline at 0 h or 72 h)." – from Figure 1 it appears that this differed by sampling site, at some hospitals (ex. SAS site 2) significant selection was only apparent when compared to the 72 h control. This statement needs to be revised to include more nuance.

The reviewer is correct. We have re-written the section to more accurately represent the results of the statistical analyses.

- Add analysis to determine if resistance selection results differ significantly between sampling sites

We can see the potential value of such a statistical comparison between wastewaters from different hospitals, but we deliberately choose not to include it to try to avoid over- or mis-interpretation of the results. As described in the manuscript, we realized in retrospect that the way we handled the samples (frozen before thawing and filtering) resulted in a loss of the overwhelming majority of the beta-lactam selection pressure. Hence, a statistical comparison between individual hospital sites would not really represent the different selection potential of the whole wastewaters, but largely reflect the fluoroquinolone-component with an unknown contribution from beta-lactams. We think that could easily become confusing. However, as pointed out, the comparison to the saline controls still shows the important message that there is a strong selection pressure for several resistances across all hospitals wastewaters, something that would not change conceptually had we handled samples in an ideal manner.

An additional reason not to compare all hospital wastewaters with each other (but just to controls) is that it would add a very large number of statistical comparisons, which would call for a much stricter multiple-comparison approach than the current one, which in turn might lead to lower statistical power to address our primary question - if each hospital wastewater selected for resistance.

- Report p-values <0.001 as “<0.001” throughout results section

Done

- Round all p-values >0.001 to 2 decimal places

Done

- Lines 169-170: explain why these 8 antibiotics were selected.

This work builds on Kraupner et al, 2021 (https://doi.org/10.1016/j.envint.2021.106436). That study used these antibiotics because they represent several major classes of antibiotics frequently used in hospitals, all with activity against *E. coli*. Also, resistances to all of these antibiotics are present in *E. coli* in wastewater communities. It was therefore considered plausible that resistance to these classes could be selected for, either directly or indirectly (co-selection by other antibiotics). Once new information came to light during the course of the present study (including both solid indications as

to the classes of antibiotics responsible for selection, as well as the strong co-selection across antibiotic classes, as apparent in e.g. extended figure 4b and 4c), these eight antibiotics were reduced to two. We have now chosen to add an explanation to this in the methods-section (lines 525-529), as we feel it would disrupt the flow in the more condensed result section.

Discussion

- Line 386 – add here data on usage of ciprofloxacin in countries other than Sweden and globally.

Appropriate references have been added to support this statement.

- Lines 395-397 – both sentences require citations for the statements made.

These are the two sentences the reviewer refers to: "*Being injectable only, cefotaxime is almost exclusively used for in-patients at hospitals. While cefotaxime is a very common antibiotic, other third-generation cephalosporins, in particular ceftriaxone, are more often used in some countries.*" We have added a reference to support the comparably higher use of ceftriaxone in e.g. the USA. We do not think a reference is needed for the first statement as we think it clear that injectable antibiotics are much more rarely used for out-patients. There are no oral formulations for cefotaxime as it is a drug with very low oral bioavailability.

- Lines 405-413: Regarding suggestions for alternative antibiotics – add information here about clinical implications (ex. side effects) of alternatives suggested.

We are not ourselves recommending any specific alternatives here, as such a suggestion (as the reviewer correctly recognizes) should involve a thorough evaluation of side effects - as well as comparisons of efficacy and costs. That is far beyond the scope of this study. What we do want to convey though is that substitution could be a realistic alternative, illustrated by cases where some hospitals have already made changes in their use of both ciprofloxacin and cefotaxime for other reasons than risks for resistance selection in the sewers. In the case of cefotaxime to pip-taz, one such reason was to reduce risks for certain side-effects (*Clostridioides difficile* infections). The last sentence of the discussion serves the purpose of pointing out that changes in antibiotic usage to reduce downstream selection in sewers should never be done at the patient's expense.

- Lines 411-413 – "This shows that some level of replacement to reduce selection risks in wastewaters is likely feasible, aiming for mutually beneficial outcomes without compromising patient safety." – add information here about what the impact is on patient safety from the suggested alternative antibiotics.

Please first see our response to the previous comment. We believe this request is beyond the scope of this manuscript. There are hospitals that intentionally have (partially) replaced cefotaxime with piperacillin-tazobactam to reduce side effects, specifically *Clostridioides difficile* infections (PhD thesis 2026 by Dr Johan Karp, https://gupea.ub.gu.se/items/d3fc8d9e-aac7-4a9f-8511-27b66eeeccf9), but we cannot find a study that comprehensively addresses all relevant clinical outcomes or health economic consequences in a comprehensive manner, focusing on these two specific treatment regimes. Therefore, we did not want to make an explicit recommendation to swap cefotaxime for piperacillin-tazobactam, but just provide the case study as an example of what might be possible. The current lack of a full evaluation of consequences on both efficacy, side effects and economy is also why we chose the phrase "aiming for".

- Need to add discussion of significance of higher resistance rates in biofilms vs wastewater

Text has been added to paragraph two of the discussion.

- Need to add limitations
- Need more information about antibiotic usage in Sweden and how it compares to other countries – are these results generalizable?
- Need information about how these results might or might not apply to other bacteria.

We have added additional discussion in paragraph 7 of the discussion. Sweden is low in total antibiotic usage (in-patient plus out-patients), following good overall adherence to guidelines. Still, antibiotic use in hospitals (in-patients) is rather typical for European hospitals, with approximately a third of the in-patients receiving antibiotic treatment at any given timepoint in both Sweden and the EU on average (referred to in the introduction, lines 54-55, references 18 and 19). Also, as expressed as DDDs (defined daily doses) per 1000 inhabitants, the in-hospital use in Sweden is rather typical for EU, whereas the outpatient use is very low (compare tables 4 and 6 in ECDCs official report (https://www.ecdc.europa.eu/en/publications-data/antimicrobial-consumption-eueea-esac-net-annual-epidemiological-report-2024). Sweden has historically invested more in outpatient care than the rest of the EU, hence Sweden has less hospital beds per capita than any other EU country (reference no 53). On average, one could therefore say it takes "a bit more" to get hospitalized in Sweden, which in turn might explain why hospital use (per patient) is very similar to the rest of EU "despite" the overall more restrictive use of antibiotics in Sweden as a whole. We have also included a reference to a study by Klein et al in PNAS from 2024 describing global trends in antibiotic consumption, noting that it refers to total use, not hospital use specifically (reference no 52). With regards to how the results might or might not apply to other bacteria, we have dedicated an entire paragraph to this aspect (lines 403-413).

Methods

- Samples were collected during different seasons from different hospitals. This could have impacted results as infections (and hence shedding) have seasonal variability. This needs to be acknowledged in a limitations section in the discussion section.

For the comparative study of resistance rates including hospitals and municipal sites within the Västra Götaland Region and Uppsala, both wastewater and biofilm sampling for resistance rates in *E. coli* were conducted during the same time period (Sep-Nov 2024 as stated in the Methods section, line 469), not during different seasons. Hence the design limits any potential influence from seasonal variability (noting also that intestinal *E. coli* are present and shed year-round by all inhabitants, largely unrelated to seasonal infections). Note that we did not analyze resistance rates in the Stockholm samples (only selection, presented in extended data, figure 1), which were collected during the winter of 2022-2023.

For our comparative selection experiments, we have in addition to samples from this defined and directly comparable time period (Sept-Nov 2024) as presented in Figure 2 (revised version) also included some samples from other seasons (in particular from the Sahlgrenska university hospital, and from the mentioned Stockholm sites). Note that here, we were interested in the presence of drivers of selection only, and not the shed bacteria as such. Hence, seasonal differences in relevant infections would likely not play any role for the outcome unless it had a marked effect on antibiotic usage, and available data does not suggest that is the case https://www.vgregion.se/halsa-och-vard/vardgivarwebben/vardriktlinjer/strama/statistik/ This is also in agreement that we do observe selection not only by wastewaters from all hospital sites in the time-matched comparison (Fig. 2), but also in all samples taken at other timepoints from the Sahlgrenska university hospital and the two Stockholm sites. Taken together, we therefore do not think it is critical to discuss differing sampling times as a limitation in the manuscript.

- Section 4.2 – why antibiotic usage data only for Region Västra Götaland?

Certainly it would have been nice to have such data from all sites. Hospital antibiotic usage data is collected at the regional level within Sweden, and we are very thankful that Region Västra Götaland compiled this data for us, as it does involve quite an effort. Not only does it involve manual curation of the database output, but prior to that it builds on detailed mapping of the underground sewer systems and identification if there were any wards that were not connected to the respective sampling points. For this reason, we were unfortunately not able to receive detailed usage data from the other

regions. However, being able to provide such high-resolution and comprehensive usage data as we do here for hospitals within the Västra Götaland Region, in combination with both wastewater concentrations, selection potential and observed resistance rates in environmental samples, is quite unique, as far as we are aware. We do not think the lack of access to detailed usage data from Uppsala (1 hospital) and Stockholm (2 hospitals) jeopardizes or changes any conclusions made.

- Lines 550-551 – add what antibiotics the blaNDM-1 and qnrB confer resistance against.

Done.

- Lines 616-617 – why these concentrations?

The concentrations of ciprofloxacin (0.5 µg/mL) and cefotaxime (2 µg/mL) were used as positive controls. They represent the clinical breakpoint concentrations for these antibiotics as defined by EUCAST, therefore, they would be expected to be inhibitory to clinically susceptible *E. coli*. Cefotaxime concentrations, 0.012 and 0.02 µg/mL corresponded to measured concentrations of cefotaxime detected in hospital wastewater 1 and hospital wastewater 2.

- Lines 632-633 - add target concentrations for DNA.

Target Concentrations added.

Reviewer #2

Summary In this study, Gray and colleagues demonstrate extensive selection for antimicrobial resistance (AMR) within antibiotic-rich hospital wastewaters. Studying wastewater samples taken from 10 hospitals in Sweden, the authors used in situ biofilm assays, metagenomics, selection experiments and microscopy to investigate selection for AMR genes and phenotypes. They found that wastewater samples from a specific hospital contained, among other antibiotics, high levels of cefotaxime and ciprofloxacin. Biofilms grown in situ within hospital wastewater pipes accumulated more resistance genes and more resistant E. coli bacteria compared with the wastewater itself, a pattern not seen in biofilms grown in municipal wastewaters. In the lab, serial passaging of a wastewater bacterial community in hospital wastewaters selected for increases in E. coli and K. pneumoniae resistance to multiple antibiotics, with the same selection absent in controls using municipal wastewater. Fluorescence microscopy showed that E. coli cells exposed to hospital wastewater displayed cell envelope perturbations consistent with those seen for beta-lactam antibiotic exposure. Finally, conferring cefotaxime and ciprofloxacin resistance genes to sensitive wastewater E. coli strain sufficed to restore growth in hospital wastewater. Together, these data suggest that Swedish hospital wastewaters drive selection for AMR, and that cefotaxime and ciprofloxacin are the primary selective drivers. Response and recommendation This manuscript addresses a crucial topic for public health: which environments drive AMR selection, and how? The authors have evidently worked hard to expand upon the scale and realism of previous investigations, using multiple orthogonal lines of evidence to rigorously demonstrate and characterise AMR selection in the wastewaters of Swedish hospitals. This manuscript therefore represents a substantial advance in our understanding of wastewater AMR drivers, and could (as the authors discuss) form the basis of targeted interventions to limit these in hospital settings worldwide. I have several criticisms (see below), but if these can be resolved I find this manuscript provisionally suitable for publication by Nature Communications. (Recommendation: Resubmission with Minor Revision.)

We thank the reviewer for their very accurate summary of our study and for recognizing the substantial effort invested in evaluating resistance selection and the drivers of selection in hospital wastewater. We appreciate that the reviewer also shares our assessment of the potentially more global implications of our work.

Major comments

1. Enrichment of resistance in biofilms does not demonstrate in situ selection. The enrichment of resistant E. coli and ARGs in general in hospital wastewater biofilms is a

very interesting effect but I fundamentally do not see how this is definitive evidence for in situ selection. This is because the enrichment of resistant strains in hospital wastewater (HWW) biofilms cf. circulating HWW could have alternative explanations not addressed here. For instance: perhaps resistant bacteria are just more likely to form biofilms (and indeed biofilm formation might contribute to their resistance phenotype). A steady flow of resistant and non-resistant bacteria would then be expected to create resistance-enriched biofilms as observed. This conceptual problem is compounded by a lack of data: only N=1 biofilms were grown at each hospital site (Fig. 2) and so there is no quantification of how variable accumulated resistance is within a given setting. Can the authors explain clearly why their data points to natural selection rather than alternative, ecological explanations (as above)? If not, I think that the authors could safely drop this claim without damaging their narrative - while they are not in situ, the HWW serial passaging experiments are compelling evidence for selection.

We understand and follow the argumentation by the reviewer. We think we are at least partially to blame for not clearly enough highlighting the counter-arguments for an alternative ecological explanation. Also as explained below, we were not clear in explaining the full line of evidence for antibiotic-driven selection of resistance from wastewater to hospital sewer biofilms:

We agree that it is conceivable that resistant bacteria might just be more likely to form biofilms than their non-resistant counterparts. If that was the simple explanation, our observation would however still represent in-situ selection of resistant bacteria into hospital biofilms, but as the reviewer points out, it would not be driven by antibiotics directly, but rather through co-selection via other traits that favor integration of resistant strains into biofilms. This would of course change both risk-scenarios and the possibilities for countermeasures. However, we do have a control for this alternative explanation - which is the wastewater-to-biofilm comparison at municipal sites (where antibiotic concentrations are much lower); In contrast to the hospital sites, we see no enrichment of resistant bacteria from the water phase to the biofilms at municipal sites. This lack of similar effects at municipal sites makes it highly unlikely that the reason for enriching resistant *E. coli* in the hospital biofilms is simply because of a link between antibiotic resistance and enhanced biofilm-forming capacity. We might add that it is of course not possible (from a science-theory point-of-view) to prove that there is no enrichment at all into biofilms at the municipal sites. But we note that biofilm-enrichment is not only non-significant at the municipal sites, the interaction term when comparing municipal and hospital sites is strongly significant, meaning that if there is any enrichment of resistant bacteria in biofilms at the municipal sites, it is clearly and significantly lower than at the hospital sites. The reviewer is correct that there is only one biofilm sampled at each site (allowed to form during the same period where the wastewater samples are taken), hence - as the reviewer points out - we cannot assess

variability of selection to biofilms within sites - which we also refrain from. We only conclude there is selection on an aggregated level (hospital sewers).

Coming back to the pedagogic problem: Previously, we showed the municipal wastewater to biofilm data in a separate figure in the annex. Now, we have merged the figures (Figure 3, main manuscript) so that the enrichment of resistant *E. coli* from water to biofilm at hospitals and the lack of corresponding enrichment at municipal sites becomes directly apparent to the reader. We have also explicitly explained in the discussion (second paragraph) that the lack of enrichment from municipal wastewater to biofilms show that it is not simply a consequence of resistant bacteria being more prone to form biofilms.

Upon reading the reviewer's comments, we also realized that we failed to highlight an important finding from one of our previous studies (Kraupner et al., 2018 https://doi.org/10.1016/j.envint.2018.04.029), which was cited but not discussed explicitly in this context. While we in the present study experimentally and directly demonstrate that ciprofloxacin and cefotaxime at measured concentrations selects for resistance in planktonic bacteria, we did not directly assess here if measured antibiotic concentrations are sufficient to drive selection into biofilms. But we did so for ciprofloxacin in the study by Kraupner et al from 2018. There, we demonstrated in controlled experiments, only varying the ciprofloxacin concentration, that measured ciprofloxacin concentrations of on average 8.8 µg/L (but not at 1.1 µg/L - note the relatively large span between tested concentrations) significantly increased the proportion of resistant over non-resistant *E. coli* in complex sewer biofilms. Notably, this concentration is frequently detected, and often exceeded, in the hospital wastewater samples analyzed in the present study. Hence, together with the results presented here, this previous experimental evidence therefore strongly supports the conclusion that antibiotics in hospital wastewater drives selection for resistance in sewer biofilms, and it is now explicitly added to the discussion (second paragraph).

One should also keep in mind the findings of full, or at least close to full, rescue of otherwise sensitive, planktonic *E. coli* by addition of resistance genes to fluoroquinolones and beta-lactams. Taken together, we do think there is strong evidence that points to natural selection (by antibiotics, with the strongest evidence for ciprofloxacin) as the cause for enrichment of resistant bacteria in hospital sewer biofilms, rather than simply other ecological explanations (noting that we are not excluding contributions from other drivers, including ecological ones). We therefore do not think there is a need to drop the claim that we observe selection of resistance in situ and that we present strong evidence that it is (at least in part) driven by excreted human pharmaceuticals. One may take a step back and compare the level of (field) evidence presented for our case with e.g. these two studies published in a prominent journal with rather claiming titles where there are no observations of effects in the field (unless drugs

were experimentally added): 1) T. Brodin et al. ,Dilute Concentrations of a Psychiatric Drug Alter Behavior of Fish from Natural Populations. Science 339, 814-815(2013). DOI:10.1126/science.1226850 2) Jack A. Brand et al., Pharmaceutical pollution influences river-to-sea migration in Atlantic salmon (Salmo salar). Science 388, 217-222 (2025). DOI:10.1126/science.adp7174.

Please see also see our response to reviewer #3, 1st comment, in particular with regards to the language used (we have replaced “conclusively linked” with “linked with strong evidence”).

2. Biotic drivers of AMR selection missing. I understand the logistical reasons for sterile-filtering HWWs prior to lab selection experiments. However, as well as altering HWW sample composition over time (Fig. 6), this will exclude potential biotic drivers of resistance evolution (e.g. microbe-microbe interactions are known to drive resistance selection - see e.g. Koch 2014 and Larsen 2022). I think this limitation, and its potential consequences for the study, should be discussed in the manuscript.

The reviewer is correct that there are limitations to laboratory experiments, in that one cannot account for every factor that may be important in the field. This was also mentioned as a limitation in the second paragraph, 7th line, of the discussion, also mentioning biotic interactions explicitly. Importantly, our *in situ* comparison of sewer biofilms and wastewater incorporates all relevant factors, including complex and variable biological interactions, thereby overcoming the limitations with experimental lab studies. Notably, our selection experiments, despite being based on filtered wastewaters, are also performed with a complex wastewater microbial community that is added to each filtered wastewater as inoculum. Hence even in the laboratory test, we do take into account biotic interactions (but admittingly in a simplified way by necessity). We think both fully complex field studies and more controlled lab studies are needed. While laboratory experiments cannot fully capture the complexity of the systems in nature, they are essential for establishing causality and mechanisms involved. With regards to the comment on altered composition after filtering, this is not what Fig 6 shows. It shows that freezing whole effluents prior to filtering alters composition (reduces certain beta-lactams) as compared to filtering fresh samples.

3. Experimental focus on E. coli lacks justification. Why are the authors focussing on resistance in E. coli (and related K. pneumoniae) specifically, when antimicrobial resistance is problematic in so many other species? This justification is currently missing but would be easy to add - e.g. does the authors’ metagenomic data demonstrate that it is one of the dominant species in HWW? Connected with this: I feel that the metagenomic data should be more prominent because it is the only dataset

shown here that generalises beyond E. coli / K. pneumoniae to other bacterial phyla. I recommend that Extended Data Figure 3 be added to / merged with the main figure list.

We fully agree with the reviewer that the challenge of antibiotic resistance extends well beyond *E. coli* and *K. pneumoniae*. There are, however, several reasons why we have focused on these species in the present study, as well as reasons why we do not wish to place greater emphasis on the metagenomic analyses in a revised version.

Recently, we published a paper in Nature Communications (Larsson et al., 2026; https://doi.org/10.1038/s41467-026-71462-4) in which we discuss why resistance selection cannot readily be inferred from metagenomic data alone. We refer the reviewer to that paper for a more comprehensive treatment of the topic. Briefly, metagenomic analyses are powerful for describing resistance gene occurrence and diversity, but they have important limitations when it comes to assessing selection processes. Changes in resistance gene abundance may reflect shifts in community composition rather than selection favoring strains with acquired resistance over susceptible counterparts of the same species (i.e. the definition of resistance selection and the key process behind resistance evolution). And shifts in community composition (in field samples) can be due to a nearly infinite number of causes.

This perspective is informed by substantial experience with environmental metagenomics. To our knowledge, we published the first study applying shotgun metagenomics to investigate antibiotic resistance in environmental samples (Kristiansson et al., 2011, https://doi.org/10.1371/journal.pone.0017038 also highlighted in Nature the same year. https://doi.org/10.1038/news.2011.46). Since then, we have contributed numerous studies in this area. Through this work, we have come to increasingly appreciate the methodological and interpretational limitations of metagenomic approaches for assessing resistance selection. In retrospect, we believe that some interpretations, including our own in earlier studies, may have been stronger than warranted by the data. Unfortunately, we also believe that resistance selection is still frequently overinterpreted from metagenomic observations alone.

For this reason, the primary approach taken in the present study is cultivation-based assessment of within-species selection, while the metagenomics analyses is considered complementary and less conclusive (please see also comment from and our response to reviewer #3, comment 5). But cultivation also has limitations, most notably that it generally allows investigation of only one species at a time. Nevertheless, it enables direct measurement of changes in the proportion of resistant and susceptible strains within a species, thereby avoiding the confounding effects of shifts in species composition.

At present, *E. coli* is essentially the only species that can be isolated from complex wastewater samples and identified to species level with very high (>99%) accuracy using relatively simple cultivation-based methods. Such accuracy is essential, as

otherwise changes in species composition could be mistaken for changes in resistance frequencies within species. More recently, we developed a cultivation protocol for *K. pneumoniae* with an identification accuracy of approximately 84% (Bobis Camacho et al., 2024; https://doi.org/10.1016/j.jgar.2024.03.005). We are currently seeking funding to extend this approach to additional bacterial species.

Our focus on *E. coli* is not motivated by a higher abundance over other taxa in wastewater, but rather by its public health relevance and methodological suitability. Resistant *E. coli* and *K. pneumoniae* are responsible for the highest number of attributed mortalities globally (Murray et al., 2022; https://doi.org/10.1016/S0140-6736(21)02724-0), with particularly high relevance in Europe. It can disseminate via wastewater systems, interact with environmental bacterial communities, exchange genetic material, and subsequently contribute to human infections. In addition, *E. coli* is intrinsically susceptible to many antibiotics, making it a useful model organism for studying resistance selection. We have devoted the 6th paragraph in the discussion to how taxonomically widespread selective effects may be, including the possible role of additional antibiotics and chemical stressors. We have also clarified the role of resistant *E. coli* and *K. pneumoniae* in our introduction.

Minor comments

1. L53: "…contaminated by use and excreted ABX". I couldn't parse this, typo?

The sentence has now been modified.

2. L66: "However, this approach relies not only on accurate determination of trace contaminants in often highly complex matrixes, but also the ability to accurately reproduce all relevant biotic and abiotic field conditions, including mixture exposures, in the laboratory experiments." Is there any evidence that the complexity of the HWW mixtures is actually important? Could the authors please discuss: does Figure 3 corroborate or dispute this motivating statement?

The cited sentence refers back to a general statement "*Comparisons of measured concentrations to effect concentrations derived from laboratory experiments is the classical way to assess environmental risks for any chemical*". There is a vast literature devoted to how various chemical components affect the toxicity of other components, one of the most well studied is how hydrogen ions (pH) affect the toxicity of metals (but also of many ionizable pharmaceuticals). In the literature, one typically refers to synergistic, additive or inhibitory effects. We refer to literature by e.g. Prof Thomas Backhaus for more reading that also includes bacterial toxicity. Additionally, one may add that complexity also matters as it frequently makes it much more challenging to measure trace contaminants correctly due to increased background signal, sometimes signal suppression and sometimes causing changes in retention times. The classical

risk assessment relies fundamentally on that the chemical analytical results are correct.

In this particular case, we show that both ciprofloxacin and beta-lactams contribute to the selection in HWW, so that is in itself evidence that mixture exposure matters. In this particular study, we have not addressed to what extent other components matter for the effect of the individual antibiotics, since here we found good agreement both between the observed effects in the field (full complexity and full relevance, but less control), observed effects by filtered wastewater in complex communities (still complex and relevant, but not being able to replicate all conditions of the sewer system) and in controlled experiments with single antibiotics (present study and also the Kraupner et al study with ciprofloxacin from 2018). And yes, we believe previous figure 3 (now figure 4) corroborates the above statement, because it shows two components of the hospital wastewater that causes antibiotic resistance selection.

3. L142: How are you identifying mobile ARGs (and why only these?) using short read data? Please clarify.

By mobile ARGs, we refer to antibiotic resistance genes that have been identified as horizontally transferable, based on their occurrence across multiple bacterial species and their unusually high sequence conservation compared with genes that are primarily vertically transmitted. This concept and methodologies used to identify such genes are described in detail in two of our recent studies namely Parras-Moltó et al, (mSpere 2025; https://doi.org/10.1128/msphere.00114-25) and Lund et al, (Nature communications 2025; https://doi.org/10.1038/s41467-025-57825-3).

Current ARG databases generally distinguish between mobile resistance genes and resistance-conferring mutations in chromosomal genes. Quantifying the relative abundance of mobile ARGs from metagenomic shotgun sequencing data is comparatively straightforward, as it primarily involves counting sequence reads that match known resistance genes in curated databases. Because mobile ARGs tend to be highly conserved, relatively stringent sequence matching criteria can be applied with confidence.

In contrast, assessing resistance-associated point mutations in chromosomal genes from metagenomic data is considerably more challenging. These chromosomal genes are often present across a large proportion of bacterial taxa, while their sequences typically exhibit varying degrees of taxonomic divergence. Consequently, identifying and quantifying specific resistance mutations requires alternative methodological approaches. For example, in an earlier study (Johnning et al., 2015; https://doi.org/10.3389/fmicb.2015.01355 as well as in the Kraupner et al 2018 study https://doi.org/10.1016/j.envint.2018.04.029), we used targeted amplicon sequencing to investigate resistance-associated mutations in gyrA and parC.

Moreover, interpretation of resistance mutation frequencies is often complex. The relevance of inferred mutation frequencies can be difficult to assess, particularly in highly diverse microbial communities. Given these methodological and interpretational challenges, we did not consider the substantial additional effort required to quantify chromosomal resistance mutations justified within the scope of the present study.

4. L390: "The generation of local usage data here should also allow for an assessment of risks for selection even at hospitals where there are no chemical analysis data of antibiotics in the wastewater". This statement contradicts your finding that the most heavily-used antibiotics (e.g. Pipercillin, L178) are not in fact the most concentrated drugs in HWW nor the most important for E. coli selection. Changing your narrative here would reinforce your point about why it is important to study HWW itself - it is difficult to infer selection from hospital usage data.

We admit that we were not completely clear on this point. It is certainly correct that it is difficult to infer selection from hospital usage data alone. If one did that naively, assuming both equal excretion rates, similar environmental fate and equal potency of all antibiotics to *E. coli*, then piperacillin-tazobactam would indeed be identified as the most important driver. What we intended to convey was that once one has identified drivers, and benchmarked these against usage data, one may extrapolate risks to other sites where only use data (not wastewater concentrations) are available. We have now revised and expanded the text slightly to make it more clear: "The generation of local usage data here should, together with identified drivers, also allow for an initial assessment of risks for selection even at hospitals where there are no chemical analysis data of antibiotics in the wastewater. Hence, with similar or higher use of ciprofloxacin and/or cefotaxime, risks are apparent"

5. Figs. 1-3: I found it counterintuitive that you report resistance changes in terms of percentage resistance - what then does it mean to have a sample where >100% of bacteria are antibiotic resistant? Using an alternative metric and/or plotting the raw data would make these quantifications more comprehensible.

We understand the confusion. Values exceeding 100% are common when plotting resistance rates derived from plating assays. They arise from unavoidable technical variability in the assay, including factors such as pipetting errors and other sources of experimental noise. These values do not reflect biologically meaningful resistance frequencies above 100%, but rather reflect the inherent measurement uncertainty associated with microbiological quantification methods.

Presenting only raw CFU counts would make it substantially more difficult to compare trends across sites, as total bacterial abundances can vary considerably between

samples. By expressing the data as resistance frequencies, we can more clearly visualize and compare temporal and spatial trends. Our objective is also not to study CFU counts per se, but rather the changes in resistance frequencies. Consequently, the relevant metric is the ratio (or percentage) of resistance colonies, with percentages simply representing ratios multiplied by 100. Please note that we report the raw CFU data as supplementary data for full transparency.

l6. Fig. 3: CTX is listed in the legend but not in the labels of the graphs themselves - where is the CTX treatment here?

This is an overlooked mistake by us. Cefotaxime used to be abbreviated in the legends within the figure but then we decided to spell out cefotaxime, so we have now removed CTX from the text legend below the figure.

7. Fig. 6: how can the ABX concentrations be increasing (beyond 100%) during freeze-thawing - does this point to a methodological flaw? Again it would be good to see raw numbers for context.

Similar to the case with % resistances above, percentages above 100 are a result of technical variability. Please see Supplementary Table 8 for the raw numbers.

8. Extended data Fig. 3: why do you think there were so few ARGs collected in Möldal HWW biofilms, compared with surrounding HWW / other locations? Please comment.

We think the most apparent possible explanation is that the taxonomic composition of Mölndal hospital wastewater biofilm differed from the other hospital biofilms, as also suggested by extended figure 2b. Different taxa carry vastly different numbers (and types) of mobile ARGs and different taxa also have inherent different sensitivities to different antibiotic classes (that is independent of mobile ARGs). If the dominant taxa in a community carry fewer mobile ARGs than other co-occurring bacteria, yet exhibit intrinsically higher resistance to ciprofloxacin and cefotaxime, this outcome is not unexpected. This scenario also illustrates why simple gene counting from metagenomic data is not a reliable approach for assessing within-species selection dynamics. Such analyses are very easily confounded in either direction by shifts in community composition. Explaining why the taxonomy differed is beyond the scope of this study, and currently we could only speculate. However, simple things like differences in water flow etc may very well matter.

9. Extended data Fig. 3: given your conclusion that ciprofloxacin and cefotaxime are the principal selective agents in HWWs, can you then see the expected enrichment of ARGs

providing resistance to these antimicrobials? If not, how does that alter your conclusions?

Both ciprofloxacin and cefotaxime strongly co-select for resistance to several other antibiotic classes (old Fig. 3, new figure 4). This is most likely due to substantial co-occurrence of ARGs (and chromosomal resistance mutations) in resistant strains. Hence, we conclude that it is highly challenging to predict the stressor (type of antibiotic) from the outcome of the selection pressure (which resistances are selected for, including which genes that are enriched), or vice versa. That is why we rely on other lines of evidence to show that ciprofloxacin and cefotaxime act as selecting agents in the hospital wastewater (and why the metagenomics is considered at most supportive a nd the corresponding figure is placed among the extended data). Although all ARG data is available as supplementary data, (link redacted due for confidentiality reasons) we chose not to report on or discuss increases or decreases of specific genes or gene classes, as it would not alter our conclusions with regards to the identification of selective agents. Note that we do observe increased phenotypic resistance to both ciprofloxacin and cefotaxime (e.g. new Fig 2).

10. Out of curiosity: do any of your data tell us whether the observed increases in resistance in HWW-cultured communities are due to demographic shifts, or de novo resistance evolution?

We interpret “demographic shifts” as “shifts in relative proportions of already existing strains of the studied microbial community”. Our approach does not distinguish between demographic shifts vs de novo resistance evolution (selection of de novo mutations arising spontaneously during the exposure). However, since we detect resistant strains also in the controls, we would assume that demographic shifts dominate, at least initially.

Reviewer #3 (Remarks to the Author):

I congratulate the authors on a strong and ambitious study. The manuscript addresses an important question in environmental antimicrobial resistance: do hospital sewers only carry resistant bacteria from patients, or do they actively select for resistance? The authors design the entire study around this gap, which is conceptually important and well-executed.

We thank the reviewer for these encouraging comments, including recognizing the importance of the presented research.

Here is my report adapted to the guidelines for referees:

KEY RESULTS

The authors investigate whether hospital sewers actively select for antibiotic-resistant bacteria or only carry resistant bacteria shed by patients. They sample wastewater and sewer biofilms from 10 Swedish hospitals and matched municipal sites, and combine community selection assays, in situ biofilm colonization on plastic plates, shotgun metagenomics, LC-MS/MS antibiotic measurements, ward-level usage data, fluorescence microscopy of MreB and MinD, and engineered isogenic E. coli strains. The central finding is that hospital wastewaters consistently select for ciprofloxacin- and cefotaxime-resistant E. coli, while municipal wastewaters do not. Resistant E. coli are also enriched in hospital sewer biofilms compared with paired wastewater, but not at municipal sites. The isogenic rescue with qnrB and blaNDM-1 identifies fluoroquinolones and β-lactams as the dominant drivers, and ciprofloxacin and cefotaxime as the principal selective agents.

SIGNIFICANCE

Earlier studies have shown higher resistance levels in hospital wastewater than in municipal wastewater, but this alone cannot separate selection from simple shedding by patients. The authors designed the study explicitly around this gap. The biofilm experiment is novel and provides (to my knowledge) some of the strongest field-level evidence to date for selection driven by excreted human pharmaceuticals in a natural environment. The identification of actionable drivers also makes the work directly relevant for hospital wastewater management and antimicrobial stewardship. As a side contribution, the freeze-thaw artifact affecting β-lactam recovery is itself useful for wastewater monitoring. The work compares well with the authors' prior single-hospital study (Kraupner et al. 2021) and clearly extends it.

VALIDITY AND SUPPORT FOR CONCLUSIONS

The central conclusion, that Swedish hospital wastewaters select for resistant E. coli, mainly through fluoroquinolones and β-lactams, is well supported by multiple independent lines of evidence that converge despite different limitations. However, several specific claims need more careful framing or additional evidence, as listed below.

We appreciate and thank the reviewer again for an excellent summary of our study.

MAJOR POINTS

1. Soften the "in-situ selection" claim. The biofilm pattern does not exclude alternative explanations, such as differential colonization, persistence, repeated seeding from upstream resistant bacteria, or site-specific physical conditions. Please change phrases such as "direct field evidence" and "conclusively linked" to "evidence consistent with" or "strong evidence supporting" in-situ selection. The abstract sentence "Here we show selection of antibiotic-resistant bacteria within Swedish hospital sewers" is too strong as written.

We first would like to refer the reviewer to our response to the first comment by reviewer #2. As stressed there, we recognize a pedagogic oversight from our side in not clearly comparing enrichment to biofilms between hospital and municipal sites (revised fig 3) as well as omitting the explicit citation of our previous study (Kraupner et al, 2018 https://doi.org/10.1016/j.envint.2018.04.029) showing that measured concentrations of ciprofloxacin enriches *E. coli* in sewage biofilms under controlled conditions.

The reviewer raises four possible alternative explanations - differential colonization, persistence, repeated seeding from upstream bacteria, or site-specific physical conditions;

For the first two, we interpret them as referring to differential biofilm colonization ability and/or higher persistence of resistant *E. coli* in biofilms over non-resistant counterparts, and that this could lead to enrichment in the biofilms without a selection pressure from antibiotics. This is basically the same the possibility as the one raised by reviewer #2 (the possibility that resistant *E. coli* are more prone to form biofilms than non-resistant counterparts). Our key argument is that if this was the main explanation, we would have expected a similar enrichment at sites with less antibiotics (municipal sites) but we do not. One cannot exclude that resistant *E. coli* are somewhat better biofilm formers, but it is not detectable in our setup and it does not explain the significant difference in enrichment from wastewater to biofilms between hospital and municipal sites. Added

to that is that exposure to measured concentrations of ciprofloxacin do lead to enrichment of resistant over non-resistant *E. coli* in complex sewage biofilms (Kraupner et al, 2018) - as well as in planktonic sewage *E. coli* (Fig. 4).

Regarding the possibility of repeated seeding by resistant bacteria from upstream sources, our study design explicitly accounts for this. The wastewater samples represent the source population seeding the biofilms, and our analyses compare resistance frequencies in these source populations directly with those observed in the corresponding biofilms. Thus, the comparison is inherently controlled for differences in the incoming bacterial communities.

With regards to site-specific physical conditions, we used a statistical analyses methodology with random effects for site, to specifically account for site-specific variability while estimating differences between wastewater and biofilms. It is of course conceivable there could be a factor X present only at hospital sites but not municipal sites (if that is what the reviewer meant with "site-specific factor") that would contribute to the selection for resistant bacteria. Note that we are not excluding additional drivers, but we argue that there is strong evidence at hand that the enrichment observed directly in situ at the hospital sites is driven at least in part by excreted antibiotics. This is supported primarily by the lack of corresponding enrichment at municipal sites, that measured concentrations of ciprofloxacin in hospital sewers leads to enrichment of resistant *E. coli* into complex sewer biofilms in controlled experiments (Kraupner et al, 2018), that measured concentrations of both ciprofloxacin and cefotaxime selects for planktonic resistant *E. coli* and that resistance genes to fluoroquinolones and beta-lactams completely or almost completely rescues otherwise sensitive strains from the growth inhibition from the hospital wastewaters.

To reviewer #2, we raised examples from language used in publications in the journal *Science* where there is vastly less evidence for effects in the field by excreted pharmaceuticals than what we present here (we do not say we agree with the language in those cases, just noting prevailing language use in other high-impact journals). We deliberately avoid the word "proof", and in line with the reviewers suggestion we have now replaced the term "conclusively linked to", but we think that the term "strong evidence for selection" is warranted and balanced. Please note that the term "evidence" in English does not mean "proof" while rather "information that increases or decreases the credibility of a hypothesis", but certainly evidence could be weak or strong. For example, finding higher ratios of resistant bacteria in hospital sewage compared to municipal sewage is also evidence for selection in the sewers, but we would refer to it as "weak evidence" as there are apparent and plausible alternative explanations (including selection in the patients). For such an observation, we think the term "evidence consistent with selection" as proposed by the reviewer would also be appropriate. A matching dose-response between lab experiments and measured

concentrations in the field would also be evidence, but stronger (noting that established effect concentrations in our study show much clearer overlap with our measured field concentrations as compared to the two mentioned *Science* papers). We do recognize that the evidence for the cause of widespread selection that we present is somewhat stronger for ciprofloxacin than for cefotaxime. This is both because we, due to imperfect handling of samples, failed to link the selection clearly to beta-lactams at most hospital sites (except for the Sahlgrenska university hospital where the beta-lactam contribution is clear), and because we only have data on that measured concentrations of ciprofloxacin leads to enrichment into biofilms (Kraupner et al, 2018) while we rely on selection potency in the planktonic phase for cefotaxime. We have therefore clarified this difference in the discussion. We see a value in neither over- nor underclaiming. Describing our findings merely as "evidence consistent with selection" is unnecessarily weak, and could be applied to many studies in the past. With regards to the sentence in the abstract "*Here we show selection of antibiotic-resistant bacteria within Swedish hospital sewers*", please note that we do show that resistant *E. coli* are enriched/selected for from the wastewater to the biofilms, and the sentence does not exclude a contribution from additional drivers beyond antibiotics, including ecological ones. After removing "conclusively linked" we think the language currently used appropriately reflects the combined evidence at hand and highlights the strengths of the study.

2. Biofilm sampling has no biological replication within sites. Each site contributed a single biofilm with technical replication only. Please acknowledge this limitation explicitly in the Discussion and state it in the Results.

It is correct that we do not have replication of biofilm samples within individual sites. For this reason, we interpret our findings at the level of hospital versus municipal systems as groups, rather than making inferences about individual sites (new Fig. 3). We have now clarified this limitation both in the results and the discussion sections.

Importantly, despite this limited replication, we observe statistically significant effects in the data. We acknowledge that low replication would be a critical limitation in cases where no significant effects were detected. In particular, for the absence of significant enrichment in biofilms at municipal sites, limited replication could in principle influence statistical power, and we have now explicitly acknowledged this limitation in the revised manuscript.

However, we also note that replication was sufficient to detect a statistically significant interaction term, which forms the central statistical basis for our conclusions. This significant interaction, together with the significant enrichment observed in hospital-

associated biofilms, supports our inference that enrichment patterns differ systematically between hospital and municipal systems.

3. Separate strong local β-lactam evidence from broader claims. Because the 2024 samples were frozen before filtration, β-lactam concentrations from this collection are unreliable. Ciprofloxacin selection is supported across all hospitals; β-lactam selection is strongly supported at Sahlgrenska and by phenotypic assays, but direct concentration-based attribution of cefotaxime across all hospitals is weaker. Please make this distinction clearly. The abstract should also briefly mention the limitations of sample handling.

The reviewer is correct. We have now explicitly added to the discussion that the evidence for selection by cefotaxime at hospitals beyond Sahlgrenska is weaker than for ciprofloxacin, for the exact reasons indicated by the reviewer. We have, however, not changed the abstract. We agree that sample handling did limit the level of evidence for cefotaxime as a selective agent broadly across all hospitals, however we do not make any specific claim in the abstract with regards to how broadly ciprofloxacin or cefotaxime selects across hospitals. Hence, what is stated in the abstract still holds. Adding explanations in the abstract that reflects this additional nuance is not very easy without losing other important aspects, given the limit of 200 words (currently 199 words).

4. Clarify the role of blaNDM-1. blaNDM-1 is a broad-spectrum carbapenemase and is unlikely to represent the dominant β-lactam resistance mechanism in Swedish hospital sewer E. coli, where ESBLs are likely more common. Please state clearly that blaNDM-1 is used as a functional rescue tool, not as a claim that NDM-like genes are the naturally selected determinants.

This is correct and we made no claims within the manuscript that NDM-1 genes were selected for by any hospital wastewater. A clarification has been added to results section 2.5 that they were created as a functional rescue tool to establish causality, not to claim that these specific genes are selected in the sewers.

5. Strengthen or moderate the metagenomics. Total mobile ARG counts per million reads are confounded by taxonomic differences (PERMANOVA $R^2 = 0.37$). If feasible, add ARG class-specific analyses, link ARGs to taxa or mobile elements, or use models accounting for taxonomy. If not feasible, please present the metagenomics as supportive context. The borderline result ($p \approx 0.047$) should be described as a "trend," not "enrichment."

We fully agree with the reviewer regarding the inherent challenges of demonstrating selection based on shotgun metagenomic analyses of ARGs in complex microbial

communities. As discussed in our response to a previous comment, we have recently addressed this issue in some more detail in Larsson et al. (2026, Nature Communications https://doi.org/10.1038/s41467-026-71462-4), which we now also cite in the revised manuscript. Accordingly, we see no possibility to reliably link ARGs to specific taxa, nor are we aware of any models that appropriately could account for taxonomy - given that assignments of mobile ARGs to host from short-read metagenomic is inherently uncertain.

In the present study, we deliberately show the figure illustrating the metagenomic results in the Extended Data rather than the main manuscript, as we consider this data as primarily supportive rather than forming the core evidence for selection. We have now further clarified this positioning in the Result section, explicitly stating that these data should be interpreted as supportive or in an exploratory manner. The inability of ARG-based assays to conclusively identify resistance selection was already stated in the discussion (6th paragraph). We have also added a reference to Larsson et al. (2026) to guide readers to a more comprehensive discussion of the limitations of this approach.

With respect to the "borderline result" highlighted by the reviewer, we report this as a "marginally significant enrichment of total ARGs," together with the corresponding p-value. Given a conventional significance threshold of $\alpha = 0.05$, we consider it appropriate to denote the result as statistically significant while simultaneously qualifying its strength using the term "marginally." We believe this provides a transparent and balanced description of the statistical evidence, appropriately conveying both its nominal significance and its limited robustness.

6. Moderate the extrapolation beyond Sweden. Sweden has unusually low community antibiotic use. Prescribing patterns, sewer infrastructure, temperature, and baseline AMR vary across countries, especially in low- and middle-income country settings.

The reviewer is correct in that Sweden is low in total antibiotic usage, following good adherence to rather strict guidelines. But with regards to the antibiotics use that matters here - the use in hospitals - just over a third of Swedish in-patients receive antibiotic treatment at any given timepoint, which also is typical for hospitals across Europe (referred to in the introduction). This can be explained because Sweden has less hospital beds per capita than any other country in the EU, so hospitalized patients in Sweden are, on average, more severely sick (which is likely linked to higher need for antibiotics), while less sick patients are typically treated as outpatients at primary health care centers.

The reviewer is correct that countries, particularly low- and middle-income countries (LMICs), often differ substantially in how waste streams are managed. However, in this

context we are discussing untreated hospital wastewater, and therefore the presence or absence of downstream wastewater treatment is not directly relevant to our argument. The key prerequisite for the processes described in our study is the existence of a water-based hospital sewage collection system through which excreted antibiotics and bacteria are transported and can interact with each other. We are aware that all hospitals in LMICs do not have water-based collection systems, but our conclusions also only pertain to those that have.

While it is correct that baseline AMR varies across countries, we do not see any obvious reason why that would conceptually affect the possibility of beta-lactams or fluoroquinolones to select for resistance in the sewers, given that selective concentrations are reached.

Temperature will differ between hospital sewers, although in an international context Sweden would probably represent the colder rather than the warmer end. As sewer pipes are typically underground, temperature is surprisingly stable across the year, in particular close to toilets as in hospital sewers. On a note, we have shown in other studies (Kraupner et al, 2021 and Huijbers et al, 2023 https://www.mdpi.com/1660-4601/20/5/4555) that if we in the laboratory compare the killing-effect at low and high temperatures of hospital wastewater, high temperature is linked to lower survival of non-resistant strains (as expected).

We write that antibiotic concentrations and use data allow extrapolation of risks beyond Sweden, which we stand behind. We do not write that results can be directly and exactly extrapolated without considerations of any other factors, but we think it is reasonable to assume that the antibiotic concentrations in hospital sewers (that in turn is a function of local use) is the main determinant for selection risks. We have added more references to international use data. With regards to cefotaxime, we highlight the uncertainty with regards to international extrapolations in the second last paragraph of the discussion "*While cefotaxime is a very common antibiotic, other third-generation cephalosporins, in particular ceftriaxone, are more often used in some countries. This suggests a larger uncertainty with regard to how widespread a significant β-lactam selection pressure is in HWWs across the world, as well as to the relative attribution to different β-lactams*."

7. Bound the taxonomic scope. Both E. coli and K. pneumoniae are members of the Enterobacterales. The conclusion that quinolones and β-lactams are the dominant drivers may not apply to anaerobes, non-fermenters, or Gram-positives. Please address this point in the Discussion.

This is correct and this is explicitly stated in paragraph 6 of the discussion.

8. Discuss non-antibiotic co-selection. Hospital wastewater contains disinfectants,

metals, biocides, and other drugs. The isogenic experiments do not exclude co-selective effects on biofilm community structure or on non-Enterobacterales taxa.

Our group has worked extensively with the possible role of co-selection from e.g. disinfectants, biocides and metals in wastewaters over the past 15 years. Still, we have not found much strong evidence (see also e.g. Yu et al, 2025 Nature communications https://doi.org/10.1038/s41467-025-65670-7). That, of course, does not rule out some undiscovered contributions, and e.g. chlorhexidine concentrations are expected to be much higher in hospital wastewaters than in municipal wastewaters. There are also some indications in the recent literature that non-antibiotic pharmaceuticals have potential to co-selection for antibiotic resistance. However, the results from the isogenic constructs presented here show that for *E. coli*, it is direct selection by quinolones and beta-lactams that matter the most, not biocides, metals, disinfectants, non-antibiotic pharmaceuticals or even antibiotics of other classes. Having said that, one can by no means exclude a role of other types of selective agents in more distant taxa or in the biofilms, as the reviewer points out. We have therefore clarified that we cannot exclude additional drivers, and added specifically that additional chemical stressors could be important selective agents in bacterial taxa with different susceptibility profiles.

9. Restrain claims about horizontal gene transfer and pathogen emergence. The study shows selection of resistant phenotypes, not mobilization or transfer. Please separate demonstrated results from inferential downstream risks.

We share the reviewer's concern of separating demonstrated effects with inferences on possible downstream risks. We do state in the introduction that "*The concern that antibiotic pollution can lead to the mobilization, transfer and irreversible establishment of novel resistance factors from diverse environmental communities into human pathogens has emphasized the need for a multisectoral approach*". That is clearly not results, but simply provides the rationale behind why we (and others) are concerned about environmental antibiotic selection pressures. We cannot find any place in the manuscript where we mix up demonstrated results from inferred risks such as mobilization or transfer.

DATA AND METHODS

The methodology is sound and thorough. Some specific issues require attention:

Sequence data availability must be rectified. I tried to access BioProject PRJNA1426335 and BioSamples SAMN55849194–SAMN55849290 on NCB/ENAI but was unable to

locate them. This may be a typo in the accession numbers, or the data may not yet be public. This must be resolved before the manuscript can proceed. Please verify the accessions, ensure the data are released or accessible to reviewers via a private link, and confirm completeness.

We apologize if the reviewer link was not made available to you. Please find the link here: (link redacted due for confidentiality reasons)

We are aware the status for the sequences is "to be released", however, we have confirmed with the editor and at this stage that is okay. All sequences will be made publicly available upon publication of this article.

Reproducibility infrastructure. The GitHub repository is well-intentioned, but some improvements are needed. The encryption of input data with a reviewer-only passkey is a barrier — please replace it with a private GitHub link, a Zenodo reviewer token, or a Figshare reviewer link. The visualization R scripts list dependencies as x.x.x rather than real version numbers; please pin versions, especially vegan and pairwiseAdonis.

We have now updated the dependencies and README.txt file to list versions as appropriate.We apologize if the reviewer password to the github datasets was not made available to you. Please find the password here:

Password to datasets in Github: Grayetal!2026

Link to repository: https://github.com/watNoel/VGRegion-Hospital-Wastewater-Selection-Project

The password is a temporary solution, upon publication the password encryption will be removed to make data available without any such restrictions.

We have now also created a figshare repository as a mirror of the github repository, where we have added the input and output files in decrypted format. The reviewer link is provided here:

https://figshare.com/s/e6211cbefaece857d58e

We hope this can ease the reviewing process.

MINOR POINTS

State the primary hypotheses explicitly at the end of the Introduction; clarify what is new compared to Kraupner et al. 2021.

We believe the last two paragraphs of the introduction already acknowledge the reviewer's request. The second to last paragraph states (in relation to Kraupner et al, 2021): "*However, it remains unknown whether wastewater from other hospitals can also promote resistance, if similar selection occurs in-situ under conditions present within the hospital sewers, and what selective agents are responsible*.". Then in the following paragraph we state our aims, which overlap with the gaps Kraupner et al. 2021 didn't answer, namely: 1) generalizability to more hospitals 2) identification of drivers through multiple lines of evidence, 3) links to usage (further allow extrapolations) 4) generating field evidence of selection (rather than relying on only laboratory experimental data). While phrased as aims, rather than hypotheses, we think they serve the same purpose.

Revise the abstract's "complete rescue" wording — qnrB alone rescued growth in most but not all samples; rescue is complete only with both genes.

The abstract states that complete rescue was achieved by the presence of fluoroquinolone **and** β-lactam resistance genes. Hence, we have acknowledged that both are required.

Clarify whether municipal samples were temporally and geographically matched to hospital samples and whether storage durations differed.

They were indeed matched both geographically and temporally. The localization of both the hospitals and municipal sites are now indicated on a map (new Fig. 1). Additionally, we have added information to clarify the matching in methods section 4.1. While we did our best to collect samples the same days from hospitals and the matching municipal sites, however there was a limit to the number of sampling machines we had access to, so in some cases there was a difference of a day or two, all dates are listed in supplementary table 9.

Clarify how biofilm resistance rates were normalized (surface area, biomass, total E. coli, DNA yield).

Resistance rates were generated by comparing CFUs on antibiotic plates versus CFUs on non-antibiotic plates (ECC CHROMagar). Hence, biofilm resistance was normalised per total *E. coli*. This is described in the methods section.

The ARG data for both biofilms and wastewater ARG counts were normalized against total reads (expressed per million reads), see methods section 4.9, "To account for variations in sequencing depth, all Diamond counts were normalized as counts per million reads.".

Report absolute E. coli counts in biofilms and wastewater, and indicate detection-limit issues.

The absolute *E. coli* count can be found in the source data file. We have now indicated this more clearly in the results section 2.2. Furthermore, we have included a statement about detection limit with the plating methodology in methods section 4.10.

Note that the wastewater-biofilm statistical comparison is count-based (the counts on antibiotic plates are the response variable, and counts on non-antibiotic plates are used as an offset, allowing us to effectively model the resistance rate). Thus, the ratio 100/1000 is not seen as equivalent to e.g 1/10 from the models perspective, and the count-based negative binomial model accounts for the greater uncertainty in low-count observations.

Indeed, as can be seen in the raw counts data, there are a number of samples where the plate counts were low, even on the non-antibiotic plates, which incurs a larger uncertainty if estimating resistance rates in those individual samples. However, in the statistical analysis of comparing biofilms to influent wastewaters in terms of resistance rates, we are not making inferences on resistance rates at specific hospital/municipal site wastewaters or biofilms but rather on the differences in rates across site- and sample types as a whole.

Describe the 90% wastewater + 10% LB / 37 °C assay as a selection-potential assay, not a reconstruction of sewer ecology.

We agree the assay is a selection-potential assay and not a “reconstruction of sewer ecology”. We have now explicitly stated this in the methods section (4.4).

For the isogenic strains, clarify possible plasmid backbone effects, expression levels, and fitness cost in non-selective wastewater.

We were aware of potential effects from the addition of a plasmid, that is why we use an isogenic approach, including both the background stain as well as the same strain with the empty plasmid strain inserted in the different assays. Hence, the key comparisons of growth between isogenic strains reflects only the difference attributable to one (or two) resistance genes (not the plasmid backbone). We acknowledge also that expression levels can influence both the level of resistance provided by the ARGs as well as the overall fitness of the strains. The level of resistance provided is indicated in Supplementary table 5. Notably, we have not used the strains in any direct competition experiment, just singly, so increased costs relative to competing strains is not really an issue here. And as figure 6a (control) shows, all strains grow similarly in 90% saline and 10% LB, hence any inferred costs are modest. The observation that expression of both ARGs leads to rescued growth in all tested waters, further ensures that excessive costs associated with the expression of the ARGs is not a major issue for the tests performed here.

Note that ResFinder annotation may miss chromosomal mutations (e.g., gyrA / parC) and divergent environmental homologs.

We are aware of this limitation and we intentionally only focus on mobile ARGs. We refer this reviewer to the more detailed response to reviewer #2 (comment 3) why we are intentionally not assessing the relative abundance of chromosomal mutations.

Acknowledge that serial-passage K. pneumoniae mutants may carry undefined additional adaptations.

This is correct. We have acknowledged this limitation in the supplementary information on preparing *K. pneumoniae* mutants.

Provide a table mapping each figure to the script that produces it; complete the empty "Citation" section in the README.

Only the metagenomic figures are produced by R. The rest are produced manually in GraphPad Prism, hence no code to refer to but all underlying data is available in the source data file. We will complete the citation in the README file when the study is accepted and has received a doi.

CLARITY, CONTEXT, AND REFERENCES

The manuscript is well-written and well-organized. The Introduction is clear and convincingly distinguishes selection from shedding. References are appropriate.

EXPERTISE

My review covers microbiology, genomics, bioinformatics, and infectious disease surveillance. I cannot fully assess the fluorescence microscopy quantification or the LC-MS/MS analytical chemistry.

SUMMARY

This is a strong, ambitious, and policy-relevant manuscript. The core conclusions are well supported. The revisions needed include reframing causal claims, clarifying the evidence for β-lactam antibiotics, acknowledging limits on biofilm replication, presenting metagenomics more conservatively, moderating extrapolation, and

improving statistical and computational reproducibility. The missing BioProject data must be resolved.

We thank the reviewer for the overall positive assessment and constructive criticism. We hope we have satisfactorily addressed all points above in our detailed responses and associated revisions.

Review of Gray *et al (2026).* "Widespread selection of antibiotic resistance within hospital sewers"
Review for *Nature Communications*

Summary
In this study, Gray and colleagues demonstrate extensive selection for antimicrobial resistance (AMR) within antibiotic-rich hospital wastewaters.

Studying wastewater samples taken from 10 hospitals in Sweden, the authors used *in situ* biofilm assays, metagenomics, selection experiments and microscopy to investigate selection for AMR genes and phenotypes. They found that wastewater samples from a specific hospital contained, among other antibiotics, high levels of cefotaxime and ciprofloxacin. Biofilms grown *in situ* within hospital wastewater pipes accumulated more resistance genes and more resistant *E. coli* bacteria compared with the wastewater itself, a pattern not seen in biofilms grown in municipal wastewaters.

In the lab, serial passaging of a wastewater bacterial community in hospital wastewaters selected for increases in *E. coli* and *K. pneumoniae* resistance to multiple antibiotics, with the same selection absent in controls using municipal wastewater. Fluorescence microscopy showed that *E. coli* cells exposed to hospital wastewater displayed cell envelope perturbations consistent with those seen for beta-lactam antibiotic exposure. Finally, conferring cefotaxime and ciprofloxacin resistance genes to sensitive wastewater *E. coli* strain sufficed to restore growth in hospital wastewater.

Together, these data suggest that Swedish hospital wastewaters drive selection for AMR, and that cefotaxime and ciprofloxacin are the primary selective drivers.

Response and recommendation
This manuscript addresses a crucial topic for public health: which environments drive AMR selection, and how? The authors have evidently worked hard to expand upon the scale and realism of previous investigations, using multiple orthogonal lines of evidence to rigorously demonstrate and characterise AMR selection in the wastewaters of Swedish hospitals. This manuscript therefore represents a substantial advance in our understanding of wastewater AMR drivers, and could (as the authors discuss) form the basis of targeted interventions to limit these in hospital settings worldwide.

I have several criticisms (see below), but if these can be resolved I find this manuscript provisionally suitable for publication by *Nature Communications*. (Recommendation: **Resubmission with Minor Revision**.)

Major comments

1. **Enrichment of resistance in biofilms does not demonstrate *in situ* selection**. The enrichment of resistant *E. coli* and ARGs in general in hospital wastewater biofilms is a very interesting effect but I fundamentally do not see how this is definitive evidence for *in situ* selection. This is because the enrichment of resistant strains in hospital wastewater (HWW) biofilms cf. circulating HWW could have alternative explanations not addressed here. For instance: perhaps resistant bacteria are just more likely to form biofilms (and indeed biofilm formation might contribute to

their resistance phenotype). A steady flow of resistant and non-resistant bacteria would then be expected to create resistance-enriched biofilms as observed. This conceptual problem is compounded by a lack of data: only N=1 biofilms were grown at each hospital site (Fig. 2) and so there is no quantification of how variable accumulated resistance is within a given setting.
Can the authors explain clearly why their data points to natural selection rather than alternative, ecological explanations (as above)? If not, I think that the authors could safely drop this claim without damaging their narrative - while they are not *in situ*, the HWW serial passaging experiments are compelling evidence for selection.

2. **Biotic drivers of AMR selection missing**. I understand the logistical reasons for sterile-filtering HWWs prior to lab selection experiments. However, as well as altering HWW sample composition over time (Fig. 6), this will exclude potential biotic drivers of resistance evolution (e.g. microbe-microbe interactions are known to drive resistance selection - see e.g. Koch 2014 and Larsen 2022). I think this limitation, and its potential consequences for the study, should be discussed in the manuscript.

3. **Experimental focus on *E. coli* lacks justification.** Why are the authors focussing on resistance in *E. coli* (and related *K. pneumoniae*) specifically, when antimicrobial resistance is problematic in so many other species? This justification is currently missing but would be easy to add - e.g. does the authors' metagenomic data demonstrate that it is one of the dominant species in HWW?
Connected with this: I feel that the metagenomic data should be more prominent because it is the only dataset shown here that generalises beyond *E. coli / K. pneumoniae* to other bacterial phyla. I recommend that Extended Data Figure 3 be added to / merged with the main figure list.

Minor comments

1. L53: *"...contaminated by use and excreted ABX"*. I couldn't parse this, typo?
2. L66: *"However, this approach relies not only on accurate determination of trace contaminants in often highly complex matrixes, but also the ability to accurately reproduce all relevant biotic and abiotic field conditions, including mixture exposures, in the laboratory experiments."* Is there any evidence that the complexity of the HWW mixtures is actually important? Could the authors please discuss: does Figure 3 corroborate or dispute this motivating statement?
3. L142: How are you identifying **mobile** ARGs (and why only these?) using short read data? Please clarify.
4. L390: *"The generation of local usage data here should also allow for an assessment of risks for selection even at hospitals where there are no chemical analysis data of antibiotics in the wastewater"*. This statement contradicts your finding that the most heavily-used antibiotics (e.g. Pipercillin, L178) are not in fact the most concentrated drugs in HWW nor the most important for *E. coli* selection. Changing your narrative here would reinforce your point about why it is important to study HWW itself - it is difficult to infer selection from hospital usage data.
5. Figs. 1-3: I found it counterintuitive that you report resistance changes in terms of percentage resistance - what then does it mean to have a sample where >100% of bacteria are antibiotic resistant? Using an alternative metric and/or plotting the raw data would make these quantifications more comprehensible.

6. Fig. 3: CTX is listed in the legend but not in the labels of the graphs themselves - where is the CTX treatment here?
7. Fig. 6: how can the ABX concentrations be increasing (beyond 100%) during freeze-thawing - does this point to a methodological flaw? Again it would be good to see raw numbers for context.
8. Extended data Fig. 3: why do you think there were so few ARGs collected in Möldal HWW biofilms, compared with surrounding HWW / other locations? Please comment.
9. Extended data Fig. 3: given your conclusion that ciprofloxacin and cefotaxime are the principal selective agents in HWWs, can you then see the expected enrichment of ARGs providing resistance to these antimicrobials? If not, how does that alter your conclusions?
10. Out of curiosity: do any of your data tell us whether the observed increases in resistance in HWW-cultured communities are due to demographic shifts, or de novo resistance evolution?

Thank you again for your submission, it was a pleasure to read. I look forward to seeing it in print.

Best wishes,

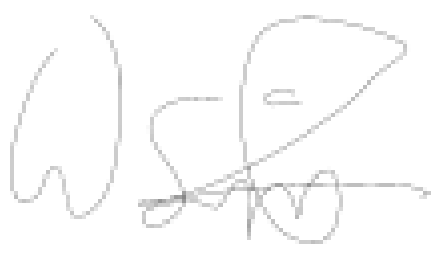

William P J Smith
Division of Evolution, Infection and Genomics | Faculty of Biology, Medicine and Health | University of Manchester
